# Iron(III) Complexes with Substituted Salicylaldehydes: Synthesis, Interaction with DNA and Serum Albumins, and Antioxidant Activity

**DOI:** 10.3390/molecules30112383

**Published:** 2025-05-29

**Authors:** Zisis Papadopoulos, Antonios G. Hatzidimitriou, George Psomas

**Affiliations:** Laboratory of Inorganic Chemistry, Department of Chemistry, Aristotle University of Thessaloniki, GR-54124 Thessaloniki, Greece

**Keywords:** iron(III) complexes, substituted salicylaldehydes, interaction with DNA, cleavage of plasmid DNA, affinity for albumins, antioxidant activity

## Abstract

Metal complexes of endogenous metals, such as iron, copper, and zinc, offer a biocompatible, cost-effective, and eco-friendly alternative to heavy metals for drug design. This study presents the synthesis, structural characterization, and evaluation of the biological activity of eight novel iron(III) complexes with substituted salicylaldehydes as ligands. The characterization of the complexes involved spectroscopic and physicochemical methods. The structures of two complexes were determined using single-crystal X-ray crystallography. The biological studies of the complexes focused on the interaction of calf-thymus DNA, the (photo)cleavage of pBR322 plasmid DNA (pDNA), the affinity for bovine and human serum albumins, and the antioxidant activity. The complexes interacted with calf-thymus DNA via intercalation with high DNA-binding constants. The complexes exhibited high pDNA-cleavage ability, which is significantly enhanced upon exposure to UVA or UVB irradiation. The complexes can bind tightly and reversibly to both serum albumins, and their binding locations were identified. Finally, the complexes showed moderate ability to scavenge 1,1-diphenyl-picrylhydrazyl and 2,2′-azinobis-(3-ethylbenzothiazoline-6-sulfonic acid) radicals with a high ability to reduce hydrogen peroxide.

## 1. Introduction

There are over eight thousand rare diseases worldwide, affecting the lives of over 350 million people. Remarkably, there are approved therapies for only 5% of them [1,2]. Furthermore, drug resistance renders many previously effective drugs inactive, increasing the need for new drugs. Metal complexes are in the spotlight because of their advantages (their variety in structure, different charge possibilities, redox activity, luminescence, magnetic properties, etc.) compared to conventional organic drugs. Until today, there have been many metal complexes that are widely used in therapy and diagnosis, such as cisplatin and other platinum drugs, as well as gadolinium and technetium-99 m complexes [3,4].

The selection of iron complexes over other metals is based on several factors that may lead to a reduction in side-effects caused by platinum drugs. Iron is an endogenous element (the most abundant transition metal) in the human body, and its biological significance has been known since antiquity [5]. Because of its ability to interconvert between oxidation states +2 and +3, iron is crucial for important biological functions, including the transportation of oxygen and electrons, cellular metabolism, respiration, DNA synthesis [6,7], and photosynthesis [8]. Despite its beneficial effects, disorders on iron homeostasis may result in iron deficiency or an iron overload, leading to heart failure [9], brain aging, and neurodegenerative diseases (such as Alzheimer’s disease and Parkinson’s disease), and its excess may result in free radical chain reactions causing tissue damages [10] or even tumors and other cancers [11,12]. It is believed that metal complexes of endogenous metals could have reduced side-effects compared to heavy metals because there is a well-regulated system of uptake, administration, and excretion in organisms. Additionally, such metal complexes suggest economically and environmentally sound options, potentially leading to cost-effective drug production [13]. Within this context, bioinorganic chemists are studying the potential activity of iron compounds, including reports regarding the antimicrobial and cytotoxic activity of iron oxide nanoparticles [14,15], as well as the anticancer [16], antimicrobial [17,18,19], and antioxidant [19,20,21,22] efficacy of iron complexes.

**Figure 1:**
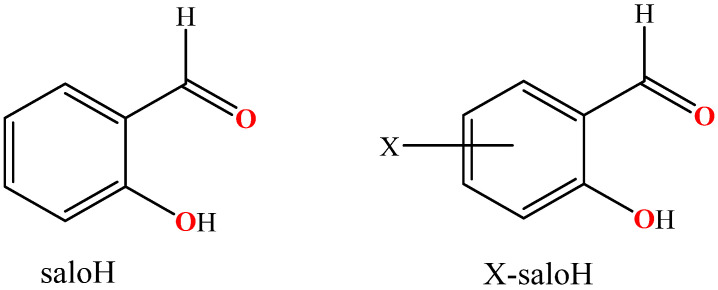
The syntax formula for salicylaldehyde (saloH) and substituted salicylaldehyde (X-saloH).

Substituted salicylaldehydes (X-saloH, Figure 1) are produced from salicylaldehyde (2-hydroxy–benzaldehyde, saloH, Figure 1), which is a natural product found in many metabolic procedures such as metabolites, intermediates, or defensive chemicals [23]. Many of the existing X-saloH compounds exhibit interesting antimicrobial and antioxidant activity, depending on the nature of the substituents [24,25,26]. This interesting biological profile, along with their ability to form stable complexes through strong bidentate coordination via the aldehyde and phenolato-oxygen atoms, has triggered many studies over the past few years. Within this context, different metal ions (such as Mn(II), Fe(III), Co(II), Ni(II), Cu(II), Zn(II), Ru(II), Pd(II), Cd(II), Mo(VI)O_2_, Re(V)O, and Er(III)) have been used for the preparation of complexes with substituted salicylaldehydes [27,28,29,30,31,32,33,34,35,36,37,38,39,40,41,42,43,44,45,46,47,48]. Although the biological properties of most of those complexes have been studied, there are not many reports on the biological activity of iron complexes with this family of ligands [49,50].

**Figure 2:**
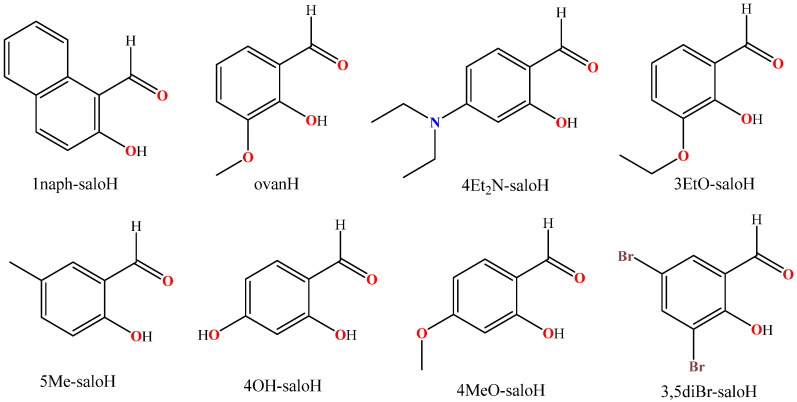
The syntax formulae of the substituted salicylaldehydes used herein (1naph-saloH = 2-hydroxy-1-naphthaldehyde; ovan = 3-methoxy-salicylaldehyde = *o*-vanillin; 4Et_2_N-saloH = 4-diethylamino-salicylaldehyde; 3EtO-saloH = 3-ethoxy-salicylaldehyde; 5Me-saloH = 5-methyl-salicylaldehyde; 4OH-saloH = 4-hydroxy-salicylaldehyde; 4MeO-saloH = 4-methoxy-salicylaldehyde; 3;5diBr-saloH = 3;5-dibromo-salicylaldehyde).

In the context of our continuous research regarding the synthesis, characterization and biological evaluation of metal complexes with substituted salicylaldehydes [27,28,29,30,31,32,33,34,35,36,37,38,39,40], the commercially available substituted salicylaldehydes 2-hydroxy-1-naphthaldehyde (1naph-saloH), 3-methoxy-salicylaldehyde (*o*-vanillin, ovanH), 5-methyl-salicylaldehyde (5Me-saloH), 3-ethoxy-salicylaldehyde (3EtO-saloH), 4-hydroxy-salicylaldehyde (4OH-saloH), 4-methoxy-salicylaldehyde (4MeO-saloH), 4-diethylamino-salicylaldehyde (4Et_2_N-saloH), and 3,5-dibromo-salicylaldehyde (3,5diBr-saloH) were employed for the synthesis of iron(III) complexes (Figure 2). The reaction of these deprotonated X-salo^−^ compounds with Fe(III) yielded eight novel neutral iron(III) complexes, namely [Fe(1naph-salo)_3_] (**1**), {K[Fe(ovan)_3_]_2_}Cl·CH_3_OH (**2**), [Fe(5Me-salo)_3_] (**3**), [Fe(3EtO-salo)_3_] (**4**), [Fe(4OH-salo)_3_] (**5**), [Fe(4MeO-salo)_3_] (**6**), [Fe(4Et_2_N-salo)_3_] (**7**), and [Fe(3,5-diBr-salo)_3_] (**8**), respectively, which were characterized with spectroscopic (IR and UV-vis spectroscopies) and physicochemical techniques. In particular, the crystal structures of complexes **1** and **2** were determined using single-crystal X-ray crystallography.

Regarding the biological activity of complexes **1**–**8**, we focused on their interaction with DNA and serum albumins, as well as their antioxidant activity. As for the interaction of complexes **1**–**8** with calf-thymus (CT) DNA, the calculation of the corresponding binding constants (K_b_) and thermodynamic parameters (ΔH, ΔS, and ΔG) in response to changes in temperature, the assessment of the DNA-interaction mode, and the competition with ethidium bromide (EB) were achieved via the combination of viscosity measurements, and UV-vis and fluorescence emission spectroscopies. The efficacy of complexes **1**–**8** in cleaving supercoiled circular pBR322 plasmid DNA (pDNA) and the effect of UVA, UVB, or visible light on this ability were evaluated using agarose gel electrophoresis. Additionally, the binding affinity and albumin-binding location of complexes **1**–**8** with bovine (BSA) and human serum albumins (HSAs) were monitored with fluorescence emission spectroscopy. Finally, the antioxidant activity of complexes **1**–**8** was assessed by examining the ability to scavenge 1,1-diphenyl-picrylhydrazyl (DPPH) and 2,2′-azinobis-(3-ethylbenzothiazoline-6-sulfonic acid) (ABTS) free radicals and their behavior towards H_2_O_2_.

## 2. Results

### 2.1. Synthesis and Characterization

**Scheme 1:**
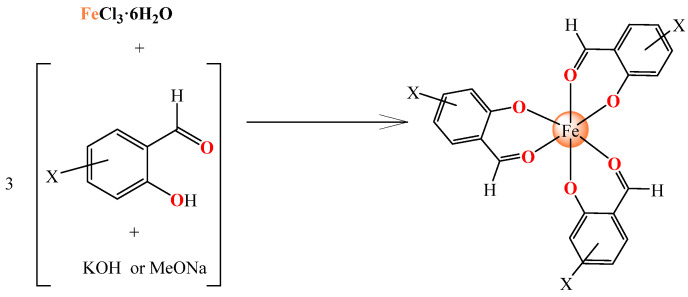
The synthetic procedure for complexes **1**–**8**.

In order to obtain complexes **1**–**8**, the substituted salicylaldehydes, after being in situ deprotonated on the phenolic hydroxyl group with a strong base (CH_3_ONa or KOH), were added in a methanolic solution of FeCl_3_·6H_2_O. This reaction efficiently yielded the final neutral homoleptic Fe(III) complexes (Scheme 1). The characterization of the complexes was achieved through physicochemical and spectroscopic techniques (IR and UV-vis) and, especially for complexes **1** and **2**, single-crystal X-ray crystallography.

All complexes were soluble in DMSO and DMF, partially soluble in methanol, and insoluble in most organic solvents and H_2_O. The molar conductivity of the complexes was measured in a 1 mM DMSO solution, and the Λ_Μ_ values were in the range of 10–16 mho·cm^2^·mol^−1^, indicating that the complexes were non-electrolytes in solution [51] bearing a 1:3 Fe(III):(X-salo) composition. These data were confirmed from elemental analysis data and were in good agreement with the suggested molecular formulae.

The changes observed in the IR spectra of complexes **1**–**8** (Figure S1), when compared to the spectra of the free salicylaldehydes, confirmed the deprotonated and the bidentate coordination of the X-salo ligands. In the spectra of free X-saloH, the stretching vibration attributed to aldehyde C=O, ν(C=O), was found in the range of 1621–1663 cm^−1^. In the spectra of the complexes, a shift in the corresponding ν(C=O) towards lower values (in the range of 1600–1626 cm^−1^ with Δν = 6–58 cm^−1^) was observed, revealing the weakening of the C=O bond as a result of the coordination of the aldehyde oxygen atoms to Fe(III) ion [28,29,30,39]. In addition, the band assigned to the stretching vibration of the phenolic C–O bond, ν(C–O), observed at 1272–1293 cm^−1^ in the spectra of free X-saloH, exhibited a positive shift in Δν = 27–44 cm^−1^ upon coordination to higher wavenumbers (in the range 1306–1324 cm^−1^) revealing the coordination of the phenolato-oxygen atoms to Fe(III) ion [28,29,30,39]. In conclusion, in all complexes **1**–**8**, the substituted salicylaldehyde ligands were coordinated to Fe(III) ions in a bidentate chelating mode through the phenolato- and aldehyde oxygens.

The electronic spectra of the complexes were measured in a DMSO solution and in a solid state. The spectra were similar, suggesting that the complexes retained their structure in solution. In the visible region of the spectrum, a band located at λ_max_ = 460–501 nm (ε = 190–600 M^−1^ cm^−1^) was observed for most complexes which may be assigned to a d–d (^6^A_1g_ → ^5^T_1g_ or ^6^A_1g_ → T_2g_(G)) transition [21,22,52]; however, in some cases, this band overlapped from the neighboring band located in the range 400–427 nm attributed to metal-to-ligand charge-transfer transitions, which is characteristic for distorted octahedral Fe^3+^ complexes with salicylaldehydes and hydroxyphenones [42,53]. In addition, the bands observed in the UV region of the spectra are attributed to intraligand transitions.

### 2.2. Structures the Complexes

Among the eight novel [Fe(X-salo)_3_] complexes studied herein, single-crystals suitable for X-ray crystallography structural determination were obtained only for complexes **1** and **2**. The experimental X-ray crystallography details for these complexes are summarized in Table S1. The structural characterization of complexes **3**–**8** was performed based on derived experimental data in comparison with existing structures.

#### 2.2.1. Crystal Structures of Complexes 1 and 2

**Figure 3:**
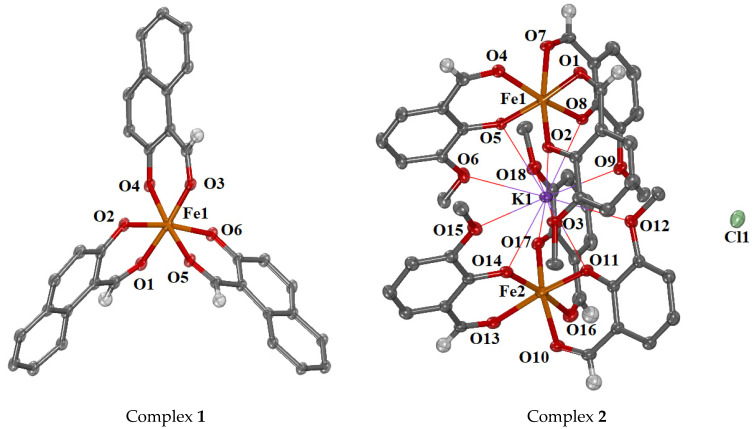
Crystal structures of complexes **1** and **2**. In complex **2**, the interactions of the hosted potassium cation (K1) with the oxygen atoms are given in thin lines. Aromatic hydrogen atoms with methanol solvate molecules and low-occupation counter anions are omitted for clarity.

Complex **1** was crystallized in the monoclinic crystal system and C2/c space group (Table S1). The molecular structure of complex **1** is shown in Figure 3, and selected bond lengths and angles are summarized in Table S2. Complex **1** is a mononuclear iron(III) complex consisting of three deprotonated 1naph-salo^−^ ligands (Figure 3). The 1naph-salo^−^ ligands are coordinated to iron(III) ions in a bidentate chelating mode through their carbonyl oxygen (O1, O3, and O5) and their phenolato (O2, O4, and O6)-oxygen atoms with Fe1–O bond distances in the range 1.965(3)–2.042(3) Å (Table 1). Such bidentate coordination is typical for the 1naph-salo^−^ ligands reported in the structures of diverse metal complexes [54,55,56]. The largest angles in the coordination sphere of Fe(III) are in the range 169.30(14)–175.24(13)° (Table S2), indicating a distorted octahedral geometry.

Complex **2** crystallized in the triclinic crystal system and *P*ī space group. The molecular structure of complex **2** is depicted in Figure 3, and selected bond lengths and angles are summarized in Table S3. The asymmetric unit of the complex contains two crystallographically independent mononuclear Fe(III) moieties of the formula [Fe(ovan)_3_], a potassium ion, a chlorido ion (disordered over two positions), and a methanol solvate molecule (disordered over two positions).

In each mononuclear iron(III) moiety, the deprotonated ovan^−^ ligands are coordinated to Fe(III) ion bidentately through their aldehyde and phenolato-oxygen atoms as previously reported for this type of complex [36,57,58,59]. The Fe–O_phenolato_ bond distances are in the range 1.933(2)–1.9384(17) Å and are shorter than the Fe–O_aldehyde_ bond distances (2.049(2)–2.0755(17) Å). Regarding the structures of iron complexes with *o*-vanillin, two reports were found in the literature [58,59], the mononuclear iron(III) complexes [Fe(ovan)_2_(H_2_O)_2_] [58] and [Fe(ovan)_2_(H_2_O)Cl] [59] which contained two ovan^−^ ligands and the methoxy oxygen did not participate in any kind of binding. Therefore, complex **2** is the first iron complex with a 1:3 Fe:(ovan^−^) ratio. The largest angles in the Fe1 and Fe2 coordination spheres are in the range 166.06(8)–170.14(7)° (Table S3), suggesting a distorted octahedral geometry around each Fe(III) ion. In both Fe(III) moieties, the corresponding oxygen atoms lie at *cis* positions to each other (O_phenolato_-Fe1/2-O_phenolato_ = 90.59 (7)–90.23 (9)°; O_aldehydo_-Fe1/2-O_aldehydo_ = 82.98 (7)–84.38 (8)°).

The relative arrangement of the two [Fe(ovan)_3_] in the lattice is such that it enables the encapsulation of a potassium ion in the interstitial space. K1 interacts electrostatically with the phenolato (O2, O5, O8, O11, O14, and O17) and methoxy (O3, O6, O9, O12, O15, and O18) oxygen atoms of the six surrounding ovan^−^ ligands at distances of 2.8616(19)–2.998(2) Å (for K1⋯O_phenolato_) and 3.054(2)–3.227(2) Å (for K⋯O_methoxy_), respectively (Table 1). The methoxy oxygen atoms of ovan^−^ ligands may participate in the coordination mainly in polynuclear complexes where *o*–vanillin acts as the bridging ligand [60,61,62,63,64,65]. The positive charge in K1 is neutralized by a chlorido anion found in the asymmetric unit.

In the literature, there are reports concerning diverse complexes hosting potassium ions. [66,67,68]. More specifically, in complex {[Ni(hab)]K[(hab)Ni]}·SCN (where H_2_hab is bis(2-hydroxy–3–methoxybenzylidene)–1,2–diaminobenzene, an *o*–vanillin derivative), the K⋯O distances are in the range 2.596 Å–2.725 Å [68]. In reported complexes and crown ethers hosting potassium ions, the K⋯O distances were found in the range 2.67–2.88 Å [66,67,69,70,71]. In complex **2**, the K⋯O distances are longer and may be considered interaction distances rather than classified as weak electrostatic or supramolecular contacts.

#### 2.2.2. Proposed Structures for Complexes **3**–**8**

The structures of complexes **3**–**8** may be proposed based on the experimental data collected from elemental analysis, molar conductivity measurements, and infrared and electronic spectroscopies. In these compounds with the general formula [Fe(X-salo)_3_], the deprotonated X-salo^−^ ligands are bound to Fe(III) ions in a bidentate fashion through the aldehyde and phenolato-oxygen atoms. In complexes **3**–**8** (Scheme 1, Figure S2), the Fe(III) ions are six-coordinated with an FeO_6_ coordination sphere adopting distorted octahedral geometry similar to that found for complex **1**.

### 2.3. Interaction of the Complexes with CT DNA

It is important to study the binding affinity of the compounds with DNA as an initial approach for further biomedical applications. The interaction of metal complexes with DNA takes place via covalent bonding or the development of non-covalent forces (leading to intercalation, electrostatic interactions, and groove-binding) or may induce the cleavage of the DNA double helix [72,73]. As a means to shed light on the nature of the interaction of complexes **1**–**8** with CT DNA, UV-vis spectroscopy, DNA viscosity measurements, and EB displacement studies were employed.

#### 2.3.1. Interaction of the Complexes with CT DNA Studied with UV-Vis Spectroscopy

UV-vis spectroscopy titrations were used to initially evaluate the interaction of complexes **1**–**8** with CT DNA. For this purpose, the UV-vis spectra of the complexes and the changes in the observed bands were monitored in the presence of incrementally increased amounts of CT DNA (Figure S3). Most of these bands showed a slight hypochromism, which was often accompanied by a bathochromic shift (Table 2). In some cases, a hyperchromic shift in the bands was also recorded. These features confirmed the interaction of the complexes with CT DNA, forming a new DNA-complex adduct and leading to a stabilized system [74]. These findings provide initial evidence of the interaction between the complexes and CT DNA. However, it is not safe to suggest a DNA-interaction mode, and for this reason, more studies are necessary.

The DNA-binding constants (K_b_) of the complexes were calculated with the Wolfe–Shimer equation (Equation (S1)) [75] and the plots of [DNA]/(ε_A_ − ε_f_) versus [DNA] (Figure S4). Most complexes **1**–**8** had higher K_b_ values than the corresponding free X-saloH; the K_b_ values were in the range 8.89(±0.14) × 10^4^ – 1.62(±0.01) × 10^7^ Μ^−1^, showing tight interactions with CT DNA (Table 2). Complex **1** exhibited the highest K_b_ value (1.62(±0.01) × 10^7^ M^−1^, Table 2) among the compounds studied herein, which was probably due to the extended aromatic system of the 1naph-salo^−^ ligand. Apart from complex **4**, the K_b_ values of all complexes **1**–**8** were higher than that of the classical intercalator EB (K_b_ = 1.23 × 10^5^ M^−1^) [76]. Compared to previously reported metal complexes with substituted salicylaldehydes [27,28,29,30,31,32,33,34,35,36,37,38,39,40], the synthesized complexes exhibited similar or higher K_b_ values and were ranked as the tightest DNA binder among the metal-(X-salo) complexes.

#### 2.3.2. CT DNA Viscosity Measurements

**Figure 4:**
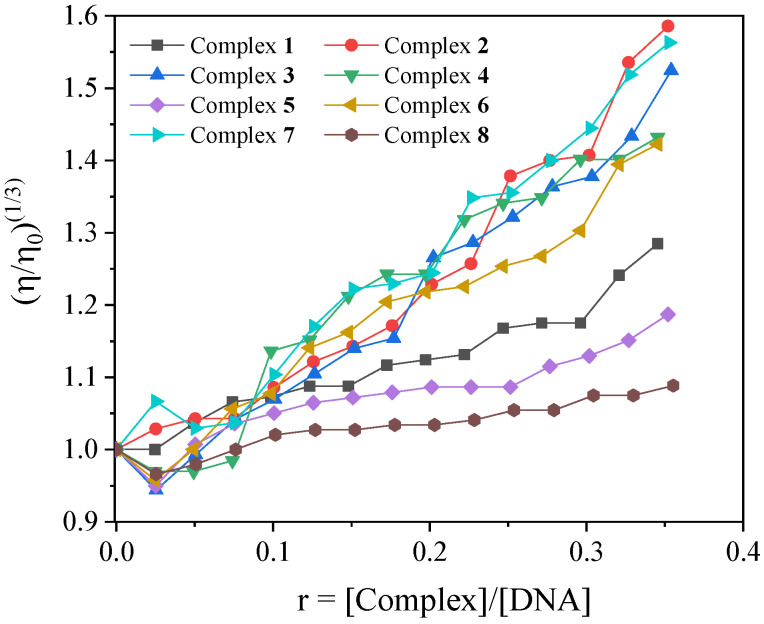
Relative viscosity (η/η_0_)^1/3^ of CT DNA (0.1 mM) in buffer solution (150 mM NaCl and 15 mM trisodium citrate at pH 7.0) in the presence of complexes **1**–**8** at increasing amounts (r = [compound]/[DNA]).

DNA viscosity measurements were performed in order to clarify the interaction mode of the complexes with DNA. Such measurements are useful since the changes in relative DNA viscosity are proportional to changes in relative DNA length. An increase in DNA viscosity is observed in the presence of classic intercalative agents whose insertion in between DNA bases increases the overall DNA-length. On the other hand, in the case of the nonclassical intercalator (i.e., including groove binders or compounds such as electrostatic interactions), practically stable or slightly decreased DNA viscosity is observed [77]. In the case of complexes **1**–**8**, the viscosity of a CT DNA solution (0.1 mM) was measured in the presence of incrementally increasing concentrations of each complex (Figure 4). For all the complexes, an increase in viscosity was noticed, which suggests an intercalative mode of interaction.

#### 2.3.3. Competitive Studies with EB

**Figure 5:**
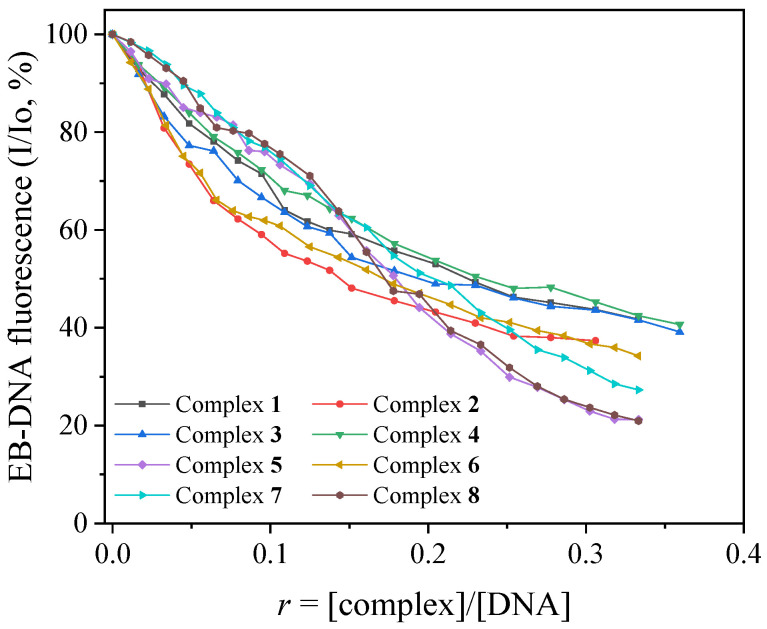
The plot of relative EB-DNA fluorescence emission intensity at λ_emission_ = 593 nm (I/Io, %) versus *r* (*r* = [complex]/[DNA]) in the presence of complexes **1**–**8** (up to 41.7% of the initial EB-DNA fluorescence for complex **1**; 37.4% for complex **2**; 39.1% for complex **3**; 40.7% for complex **4**; 21.2% for complex **5**; 34.2% for complex **6**; 27.3% for complex **7**; and 20.9% for complex **8**).

EB is a DNA-intercalation marker since its intercalation to DNA, which occurs via the insertion of its planar phenanthridine ring in between two adjacent DNA bases, is evident from an intense fluorescence emission band at λ_max_ = 592–594 nm upon excitation at 540 nm [78]. The quenching of this band’s intensity induced by the presence of the complexes will indicate their competition for the same DNA intercalation sites [78]. Therefore, a buffer solution of EB (40 μM) and CT DNA (40 μM) was pretreated for 1 h, and its fluorescence emission spectra were recorded (with λ_excitation_ = 540 nm) in the presence of incrementally increasing concentrations of each complex (Figure S5). An intense quenching of the EB-DNA emission band at λ_max_ = 593 nm was observed for all complexes (up to 79.1% of the initial fluorescence recorded for complex **8**, Figure 5, Table 3).

The Stern-Volmer constants (K_SV_) were calculated with the Stern-Volmer equation (Equation (S2)) [78] and the corresponding plots (Figure S6). All complexes presented relatively high K_SV_ values (Table 3), and complex **4** had the highest constant (K_SV_ = 1.35(±0.02) × 10^5^ M^−1^). For the calculation of the quenching constants (K_q_) for the complexes with Equation (S3), the value of 23 ns was applied as the fluorescence lifetime for the EB-DNA system (τ_0_) [79]. The K_q_ values of all complexes were significantly higher (by two orders of magnitude) than 10^10^ M^−1^ s^−1^. Such high K_q_ values are evidence of a static quenching mechanism, confirming the formation of a new DNA compound adduct as a result of the displacement of EB [78].

#### 2.3.4. Thermodynamic Parameters for the Interaction of Complexes with CT DNA

The non-covalent forces developed between compounds and DNA are hydrophobic forces, electrostatic interactions, van der Waals interactions, and hydrogen bonds [80,81]. According to the literature, the signs of the values calculated for the changes in enthalpy (ΔH) and entropy (ΔS) may serve as evidence of the different interaction modes. More specifically, positive values of both ΔH and ΔS (ΔH > 0 and ΔS > 0) are indicative of the presence of hydrophobic forces, and negative values of both ΔH and ΔS (ΔH < 0 and ΔS < 0) are found upon the development of van der Waals interactions, while the combination of ΔH < 0 and ΔS > 0 is consistent with electrostatic interactions [81,82].

The K_b_ values of complexes **1**–**8** were determined for three different temperatures (298 K, 303 K, and 310 K). For all complexes **1**–**8**, the increase in temperature resulted in higher K_b_ values (Table S4). The corresponding changes in enthalpy (ΔH) and entropy (ΔS) (Table S4) were calculated from the van’t Hoff equation (Equation (S4)), and ΔG was obtained from the Gibb’s-Helmholtz equation (Equation (S5)) and from the plots of ln(K_b_) versus (1/T) complexes **1**–**8** (where –ΔH/R is the slope and ΔS/R is the intercept of the fitting line while R is the universal gas constant, as shown in Figure S7). Both the values of ΔH and ΔS (Table S4) are positive, indicating that hydrophobic forces are developed between the complexes and CT DNA upon their interaction, i.e., π–π stacking interactions, which are consistent with an intercalative interaction mode [83]. Furthermore, the negative ΔG values (Table S4) indicate a spontaneous interaction with CT DNA [81,83,84].

### 2.4. (Photo)Cleavage of pBR322 Plasmid DNA

The interaction of complexes **1**–**8** with plasmid DNA was studied in the presence and absence of irradiation. The complexes (500 µM) were incubated with pBR322 DNA in a tris buffer solution (25 μM, pH 6.8), ensuring that the final concentration of DMSO did not exceed 10% *v*/*v*. The effect of the compounds on pDNA was monitored after incubating the samples at 37 °C in the absence or presence of UV-B (irradiation at 312 nm for 30 min), UV-A (irradiation at 365 nm for 120 min), or visible light (irradiation for 120 min), and the results were analyzed with gel electrophoresis on 1% agarose stained with EB. The supercoiled pDNA appears as Form I in the gel after electrophoresis. The interaction of the compounds with pDNA may induce single-stranded (ss) or double-stranded (ds) damage, resulting in Form II (relaxed pDNA) and Form III (linear pDNA), respectively. In this case, the extent of pDNA damage is assessed by calculating the percentages of ss% and ds% with Equations (S6) and (S7) [85].

The reaction mixtures of pDNA and the compounds were incubated in the dark for 150 min and were then analyzed by 1% agarose gel electrophoresis with EB staining (Figure S8). In the absence of light, all complexes (500 μM) converted supercoiled pDNA into relaxed circular DNA (Form II) by inducing ss breaks with low-to-moderate percentages (up to 43% induced by complex **8**).

The complexes proved to be more active when the mixture of pDNA compounds was exposed to radiation. When exposed to UVB radiation for 30 min, supercoiled pDNA was almost completely degraded due to the ss and ds breaks, and in some lanes, the smearing did not allow the respective percentages to be calculated (Figure S9). When exposed to UVA radiation for 120 min, the complexes were very active at inducing ss breaks to pDNA (Figure S10). In most cases, bands exhibiting delayed electrophoretic mobility compared to Form II of pDNA, which was observed and may be attributed to pDNA fragments of higher molecular weight [86,87]. Finally, the exposure to visible light resulted in the less pronounced activity of the complexes towards pDNA (Figure S11), causing ss and ds (of lower percentage) breaks.

In conclusion, exposure time and irradiation energy affected the photocleavage activity of the compounds. All the complexes became very active after exposure to UVA or UVB radiation at the concentration (of 500 µM) tested, showing their photoreactive potential.

### 2.5. Interaction of the Compounds with Albumins

Serum albumin (SA) is among the most abundant proteins of the circulatory system, with many biological roles, such as the regulation of normal blood volume and osmotic pressure and the reversible binding and transportation of drugs and other bioactive small molecules. Therefore, studying the interaction between albumins (either HSA or its structural analog BSA) with bioactive compounds may contribute to revealing altered new mechanistic pathways or differentiated biological properties of these compounds upon their interaction with SAs [78]. Within this context, the interaction of complexes **1**–**8** with BSA and HSA was studied with fluorescence emission spectroscopy.

**Figure 6:**
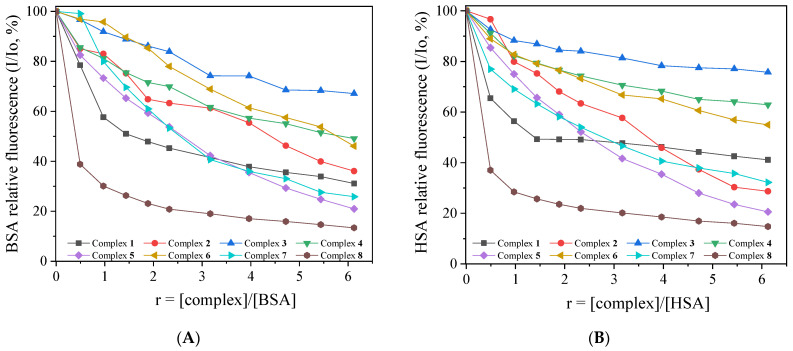
(**A**) Plot of % relative fluorescence intensity of BSA at λ_em_ = 344 nm (I/Io, %) versus *r* (*r* = [complex]/[BSA]) complexes **1**–**8** (up to 31.1% of the initial BSA fluorescence **1**; 36.1% for **2;** 67.1% for **3**; 49.1% for **4**; 20.9% for **5**; 46.1% for **6**; 25.8% for **7**; and 13.4% for **8**). (**B**) Plot of % relative fluorescence intensity of HSA at λ_em_ = 342 nm (I/Io, %) versus *r* (*r* = [complex]/[HSA]) for complexes **1**–**8** (up to 41.1% of the initial HSA fluorescence for **1**; 28.7% for **2;** 75.7% for **3**; 62.9% for **4**; 20.6% for **5**; 54.9% for **6**; 32.2% for **7**; and 14.7% for **8**).

The solutions of BSA and HSA exhibited an intense fluorescence emission band with λ_em,max_ in the range of 340–350 nm, when excited at 295 nm [78] because of the tryptophan residues (Trp-134 and Trp-212 in BSA, and Trp-214 in HSA). The addition of complexes **1**–**8** into an SA solution (3 μM) resulted in a moderate-to-significant quenching of the albumin fluorescence emission bands (Figures S12 and S13). Complex **8** induced the highest quenching for both albumins (Figure 6). The observed quenching was assigned to changes in the tryptophan environment of SA due to changes in its secondary structure resulting from the binding of the compounds to SA [78]. Furthermore, the influence of the inner-filter effect on the measurements was evaluated with Equation (S8) [88], but it was too low to affect the measurements.

Stern-Volmer and Scatchard equations (Equations (S2), (S3) and (S9)) and plots (Figures S14–S17) were used to calculate the corresponding SA-quenching constants (K_q_) and the SA-binding (K) constants, respectively. For the calculation of K_q_, the fluorescence lifetime of tryptophan in SA is τ_ο_ = 10^−8^ s [78]. For all compounds, the calculated K_q_ values (Table 4) are approximately two–three orders higher than the value 10^10^ M^−1^ s^−1^. Therefore, a static quenching mechanism may be suggested [78] to confirm the interaction of the compounds with the albumins. Regarding the K values, they are of the 10^4^–10^6^ M^−1^ order and are in the range found for other metal complexes with substituted salicylaldehydes [27,29,30,32,33,34,36,37,38,39]. All complexes **1**–**8** have K values lower than 10^15^ M^−1^, which is the association constant of avidin with diverse compounds and is considered the highest of known noncovalent interactions. Such values show the reversible binding of SAs with the complexes, which, therefore, can be transferred and released to the desired bio-targets [89].

The identification of the albumin binding site of a compound is often required to understand their interaction. According to crystallography, the key sites for the attachment of drugs and metal ions are Sudlow’s site one (or drug site I) in subdomain IIA and Sudlow’s site two (or drug site II) in subdomain IIIA. In order to study the binding selectivity of the compounds towards these albumin-binding sites, warfarin and ibuprofen are used as site markers, respectively [90]. For this purpose, titration fluorescence quenching studies were performed in the presence of warfarin or ibuprofen (Figures S18–S21), and the corresponding K values for the compounds were calculated (Table 4) with the Scatchard equation (Equation (S9)) and corresponding plots (Figures S22–S25). If the presence of a marker influences the binding of the compound competitively (i.e., both the marker and the compound bind in the same binding site), then a decrease in the corresponding K value will be observed, indicating the selectivity for the same binding site of respective marker [90].

In the case of BSA, complexes **2**, **4**, and **7** present significantly lower K values in the presence of warfarin, which suggests that they have a binding selectivity for drug site I. On the other hand, complex **5** exhibits a lower K value in the presence of the ibuprofen marker, indicating a preference to bind to Sudlow’s site II. For complexes **1**, **3**, **6**, and **8**, a safe conclusion concerning the binding preference for sites I or II is not obvious since the K_(marker)_ values are similar. Regarding HSA, all complexes **1**–**8** present significantly lower K values in the presence of ibuprofen, suggesting their selective attachment to Sudlow’s site II.

### 2.6. Antioxidant Activity of the Complexes

Free radicals are reactive chemical species with unpaired electron(s) and may usually induce inflammations [91]. They are produced as part of mitochondrial metabolism and may attack DNA, proteins, and lipids. On the counterpart, antioxidants are compounds that delay or prevent the oxidation of these substrates [92]. Natural antioxidants are usually organic compounds such as phenolic acids, flavones, and flavonoids. However, a combination of the redox properties of metal ions with various ligands may lead to effective metal-based antioxidant compounds [92]. Within this context, the ability of complexes **1**–**8** to scavenge DPPH and ABTS radicals and to reduce H_2_O_2_ was studied and compared with the well-known antioxidant agents NDGA, BHT, trolox, and L-ascorbic acid, which are widely used standard reference antioxidants [93,94].

The ability of compounds to scavenge DPPH radicals is often related to preventing aging, cancer, and inflammation [95]. The method is based on the discoloration of the violet-colored methanolic solution of DPPH in the presence of the compounds [95]. The DPPH scavenging ability may also evaluate the antioxidant capacity of coordination compounds [92]. Most of the complexes under study are inactive or exhibit low activity towards DPPH radicals, which are found to be time-dependent for complexes **2** and **4** only (Table 5). Complex **2** is the most active compound herein towards DPPH radicals (%DPPH scavenging ability up to 49.03 ± 0.71%) and is among the most active reported metal complexes of substituted salicylaldehydes [27,29,30,32,33,38,39]. All complexes **1**–**8** are less active than the reference compounds NDGA and BHT.

The ability of a compound to scavenge the cationic ABTS radicals (ABTS^+●^) is used as a marker of the total antioxidant activity [95]. The assay is based on the discoloration of a dark green solution with the cationic radical ABTS^•+^, which is induced by the compounds [95]. The ABTS scavenging ability is low-to-moderate. Such differentiation of the ABTS scavenging ability depends on the nature of X-saloH as it was previously reported for Mn(II), Cu(II), Zn(II), Pd(II), and Gd(III) complexes with substituted salicylaldehydes [27,29,30,32,33,38,39]. However, the activity of the most active compounds, namely complexes **2** and **5** (= 85.69 ± 0.67% and 84.72 ± 0.76%, respectively), was close to the activity of the reference compound trolox (Table 5).

The interaction of the compounds with hydrogen peroxide (which produces hydroxyl radicals) may serve as a marker of inhibition of reactive oxygen species, offering protection from oxidative stress [96]. The ability of complexes **1**–**8** to reduce H_2_O_2_ is comparable with that of reference compound L-ascorbic acid (Table 5). Complex **8** presents the highest percentage of H_2_O_2_ reduction (H_2_O_2_% = 85.86 ± 0.89%) and is the most active among the compounds under study. The behavior of the complexes is similar to that reported for a series of Mn(II), Cu(II), Zn(II), Pd(II), and Gd(III) complexes with substituted salicylaldehydes [27,29,30,32,33,38,39].

In total, complexes **1**–**8** were practically inactive towards DPPH radicals except for complex **2**, which presented a moderate DPPH scavenging activity. Regarding ABTS radicals, only complexes **2** and **5** approached the activity of the reference compound trolox. All complexes studied herein exhibited toward hydrogen peroxide comparable activity with the reference compound L-ascorbic acid, with complex **8** being the most active compound.

## 3. Materials and Methods

### 3.1. Materials-Instrumentation-Physical Measurements

All chemicals and solvents were reagent grade and were used as purchased from commercial sources: sodium citrate, NaCl, CT DNA, EB, BSA, HSA, and ABTS were purchased from Sigma-Aldrich Co; BHT, NDGA, K_2_S_2_O_8_, and trolox were purchased from J&K Scientific Co; 1naph-saloH, ovanH, 5Me-saloH, 3EtO-saloH, 4OH-saloH, 4MeO-saloH, 4Et_2_N-saloH, sodium warfarin, ibuprofen, and DPPH were purchased from Tokyo Chemical industry (TCI); 3,5diBr-saloH was purchased from Fluorochem; Tris base, EDTA disodium salt dehydrate, loading buffer, and H_2_O_2_ (30% *w*/*v*) were purchased from PanReac AppliChem ITW Reagents Co; supercoiled circular pBR322 plasmid DNA was purchased from New England Bioline; FeCl_3_∙6H_2_O, CH_3_ONa, KOH, L-ascorbic acid, Na_2_HPO_4_, NaH_2_PO_4_, HCl (35% *v*/*v*) and all solvents were purchased from Chemlab Co.

The DNA stock solution was prepared by the dilution of CT DNA to a buffer solution (containing 150 mM NaCl and 15 mM sodium citrate at pH 7.0) followed by stirring at 4 °C and was kept at 4 °C for no longer than two weeks. The stock solution of CT DNA gave a ratio of UV absorbance at 260 and 280 nm (A_260_/A_280_) in the range of 1.88–1.90, indicating that DNA was sufficiently free of protein contamination [97]. The DNA concentration was determined by the UV absorbance at 260 nm after a 1:20 dilution using ε = 6600 M^−1^ cm^−1^ [98].

Infrared (IR) spectra were obtained on a Thermo Scientific Nicolet iS20 FTIR ATR spectrometer without any treatment of the solid sample in the range of 400–4000 cm^−1^ (abbreviations used included vs. = very strong; s = strong; sm = strong-to-medium; and m = medium). The UV-vis spectra were recorded in the range 200–800 nm as solid and in solution (concentrations in the range of 10 µM–1 mM) on a Jasco V-750 spectrophotometer (abbreviation used included sh = shoulder). The spectra were recorded in DMSO solutions using quartz cells with an optical path of 1 cm sealed tightly with Teflon caps. The fluorescence emission spectra were recorded in solution in quartz cells (1 cm) on a Hitachi F-7000 fluorescence spectrophotometer. C, H, and N elemental analyses were performed on a Perkin Elmer 240B elemental analyzer. The molar conductivity measurements were carried out on a 1 mM DMSO solution of the complexes with a Crison Basic 30 conductometer. The viscosity experiments were conducted using an ALPHA L Fungilab rotational viscometer equipped with an 18 mL LCP spindle.

### 3.2. Synthesis of the Complexes

All complexes were prepared at room temperature according to the following procedure: KOH (0.3 mmol, 300 μL of 1 Μ solution) or CH_3_ONa (0.3 mmol, 16 mg) was added into a methanolic solution (5–10 mL) of the corresponding X-saloH (0.3 mmol) under stirring for 45 min in order to deprotonate the X-saloH. Afterward, the resultant solution was added dropwise to a methanolic solution of FeCl_3_·6H_2_O (0.1 mmol, 27 mg). The final solution was stirred for an additional 45 min, then was filtered and left to evaporate slowly at room temperature. After a few days, the desired product was suspended in a minimal amount of methanol and filtered and washed with water and diethyl ether or dichloromethane.

**[Fe(1naph**-**salo)_3_] (1):** For the synthesis of complex **1**, 1naph-saloH (0.3 mmol, 52 mg) was the corresponding X-saloH and was deprotonated with CH_3_ONa (0.3 mmol, 16 mg). After ten days, red-brown single crystals of [Fe(1naph-salo)_3_] suitable for X-ray structural determination were collected (Yield: 40 mg, 70%). Anal. calcd. for C_33_H_21_FeO_6_ (MW = 569.37): C 69.61, H 3.72; found C 69.42, H 3.61%. FT-IR (ATR), *ν*_max_/cm^−1^: *ν*(C=O)_aldehydo_, 1614 (s); *ν*(C–O)_phenolato_, 1306 (m). UV-vis: solid, λ/nm: 510, 410; in DMSO, λ/nm (ε/M^−1^ cm^−1^): 501 (190), 425 (5900), 410 (6300), 368 (4500), 306 (sh) (7000), 298 (sh) (7900), 269 (12,500). Complex **1** is soluble in DMF and DMSO (Λ_M_ = 11 mho·cm^2^·mol^−1^, 1 mM DMSO) and partially soluble in methanol.

**{K[Fe(ovan)_3_]_2_}Cl·MeOH (2)**: For the synthesis of complex **2**, KOH (0.3 mmol, 300 μL of 1 Μ solution) deprotonated ovanH (0.3 mmol, 46 mg) which was the corresponding X-saloH. Dark-red single crystals of {K[Fe(ovan)_3_]_2_}Cl·MeOH suitable for X-ray structural determination were collected after a week (Yield: 90 mg, 80%). Anal. calcd. for C_49_H_46_ClFe_2_KO_19_ (MW = 1125.14): C 52.31, H 4.12; found C 52.15, H 4.30%. FT-IR (ATR), *ν*_max_/cm^−1^: *ν*(C=O)_aldehydo_, 1626 (m); *ν*(C–O)_phenolato_, 1313 (m). UV-vis: solid, λ/nm: 510, 405; in DMSO, λ/nm (ε/M^−1^ cm^−1^): 475(sh) (300), 385(sh) (3500), 355 (7500), 320 (6500), 278 (6700). Complex **2** is soluble in DMF and DMSO (Λ_M_ = 15 mho·cm^2^·mol^−1^, 1 mM DMSO).

**[Fe(5Me**-**salo)_3_] (3):** For the synthesis of complex **3**, CH_3_ONa (0.3 mmol, 16 mg) was used for the deprotonation of the corresponding X-saloH, namely 5Me-saloH (0.3 mmol, 41 mg). After two weeks, the formation of the dark-red precipitate of [Fe(5Me-salo)_3_] was observed and the product was collected with filtration and washed with water and diethyl ether (Yield: 30 mg, 30%). Anal. calcd. for C_24_H_21_FeO_6_ (MW = 461.27): C 62.49, H 4.59; found: C 62.25, H 4.33%. FT-IR (ATR), *ν*_max_/cm^−1^: *ν*(C=O)_aldehydo_, 1622 (s); *ν*(C–O)_phenolato_, 1312 (sm). UV-vis: solid, λ/nm: 498, 415; in DMSO, λ/nm (ε/M^−1^ cm^−1^): 518 (sh) (200), 410 (400), 335 (5600). Complex **3** is soluble in DMF and DMSO (Λ_M_ = 11 mho·cm^2^·mol^−1^, 1 mM DMSO) and partially soluble in methanol.

**[Fe(3EtO**-**salo)_3_] (4):** For the synthesis of complex **4**, the deprotonation of 3EtO-saloH (0.3 mmol, 50 mg), the corresponding X-saloH, was achieved with CH_3_ONa (0.3 mmol, 16 mg). The dark-red precipitate of [Fe(3EtO-salo)_3_] was collected with filtration and washed with water and diethyl ether (Yield: 40 mg, 72%) after two weeks. Anal. calcd. for C_27_H_27_FeO_9_ (MW = 551.35): C 58.82, H 4.94; found C 58.55, H 5.09%. FT–IR (ATR), *ν*_max_/cm^−1^: *ν*(C=O)_aldehydo_, 1600 (vs); *ν*(C–O)_phenolato_, 1319 (sm). UV-vis: solid, λ/nm: 517; in DMSO, λ/nm (ε/M^−1^ cm^−1^): 519 (250), 405 (2700), 340 (7500), 272 (sh) (16,000). Complex **4** is soluble in DMF and DMSO (Λ_M_ = 15 mho·cm^2^·mol^−1^, 1 mM DMSO) and partially soluble in methanol.

**[Fe(4OH**-**salo)_3_] (5):** For the synthesis of complex **5**, 4OH-saloH (0.3 mmol, 41 mg) was the corresponding X-saloH and was deprotonated with CH_3_ONa (0.3 mmol, 16 mg). The red-brown precipitate of [Fe(4OH-salo)_3_] was collected with filtration and washed with water and diethyl ether after a few days (Yield: 40 mg, 85%). Anal. calcd. for C_21_H_15_FeO_9_ (MW = 467.19): C 53.99, H 3.24; found C 54.15, H 3.45%. FT-IR (ATR), *ν*_max_/cm^−1^: *ν*(C=O)_aldehydo_, 1615 (m); *ν*(C–O)_phenolato_, 1324 (s). UV-vis: solid, λ/nm: 495; in DMSO, λ/nm (ε/M^−1^ cm^−1^): 505(sh) (190), 353(sh) (4500), 316 (15,000), 280 (1800). Complex **5** is soluble in DMF and DMSO (Λ_M_ = 10 mho·cm^2^·mol^−1^, 1 mM DMSO) and partially soluble in methanol.

**[Fe(4MeO-salo)_3_] (6):** For the synthesis of complex **6**, CH_3_ONa (0.3 mmol, 16 mg) was used to deprotonate the corresponding X-saloH, i.e., 4MeO-saloH (0.3 mmol, 46 mg). After a few days, a dark-red product of [Fe(4MeO-salo)_3_] was collected with filtration and washed with water and diethyl ether (Yield: 25 mg, 49%). Anal. calcd. for C_24_H_21_FeO_9_ (MW = 509.27): C 56.60, H 4.16; found C 56.45, H 4.32%. FT-IR (ATR), *ν*_max_/cm^−1^: *ν*(C=O)_aldehydo_, 1625 (m); *ν*(C–O)_phenolato_, 1307 (m). UV-vis: solid, λ/nm: 465; in DMSO, λ/nm (ε/M^−1^ cm^−1^): 485 (350), 403 (1800), 318(sh) (9800), 285 (15,700). Complex **6** is soluble in DMF and DMSO (Λ_M_ = 14 mho·cm^2^·mol^−1^, 1 mM DMSO) and partially soluble in methanol.

**[Fe(4Et_2_N**-**salo)_3_] (7):** For the synthesis of complex **7**, the corresponding X-saloH was 4Et_2_N-saloH (0.3 mmol, 58 mg) and was dissolved in a 1:1 CH_3_OH/CH_2_Cl_2_ mixture (10 mL). 4Et_2_N-saloH was deprotonated with KOH (0.3 mmol, 300 μL of 1 Μ solution). After two weeks, the dark-red precipitate of [Fe(4Et_2_N-salo)_3_] was collected with filtration and washed with water and dichloromethane (Yield: 50 mg, 79%). *Anal.* calcd. for C_33_H_42_FeN_3_O_6_ (MW = 632.56): C 62.66, H 6.69, N 6.64; found C 62.41, H 6.43, N 6.47%. FT-IR (ATR), *ν*_max_/cm^−1^: *ν*(C=O)_aldehydo_, 1615 (s); *ν*(C–O)_phenolato_, 1315 (sm). UV-vis: solid, λ/nm: 495, 403; in DMSO, λ/nm (ε/M^−1^ cm^−1^): 485 (600), 407 (5100), 350 (15,300), 267 (sh) (8900). Complex **7** is soluble in DMF and DMSO (Λ_M_ = 12 mho·cm^2^·mol^−1^, 1 mM DMSO) and partially soluble in methanol.

**[Fe(3,5diBr**-**salo)_3_] (8):** For the synthesis of complex **8**, CH_3_ONa (0.3 mmol, 16 mg) was used for the deprotonation of 3,5diBr-saloH (0.3 mmol, 84 mg) which was used as the corresponding X-saloH. A small quantity (5 mL) of hexane was added for slow vapor diffusion. The orange microcrystalline product of [Fe(3,5diBr-salo)_3_] (**8**) was formed after two weeks, collected with filtration, and washed with water and diethyl ether (Yield: 50 mg, 56%). Anal. calcd. for C_21_H_9_Br_6_FeO_6_ (MW = 892.60): C 28.26, H 1.02; found C 28.52, H 1.18%. FT-IR (ATR), *ν*_max_/cm^−1^: *ν*(C=O)_aldehydo_, 1606 (vs); *ν*(C–O)_phenolato_, 1311 (sm). UV-vis: solid, λ/nm: 498, 405; in DMSO, λ/nm (ε/M^−1^ cm^−1^): 500(sh) (250), 427 (500), 320 (1400), 294 (10,700), 268 (4000). Complex **8** is soluble in DMF and DMSO (Λ_M_ = 16 mho·cm^2^·mol^−1^, 1 mM DMSO) and partially soluble in methanol.

### 3.3. Single-Crystal X-Ray Crystallography

Suitable single crystals of compounds **1** and **2** were mounted on thin glass fibers with the aid of epoxy resin. X-ray diffraction data were recorded on a Bruker Kappa Apex II CCD area-detector diffractometer equipped with a Mo Ka (λ = 0.71073 Å, sealed tube source operating at 50 kV and 30 mA) and a Triumph monochromator at 295 K, using the φ and ω scanning technique. The program Apex2 (Bruker AXS, 2006) was used for data collection and cell refinement. The collected data were integrated with the Bruker SAINT software package [99] using a narrow frame algorithm. Data were corrected for absorption using the numerical method SADABS [100] based on the crystal dimensions. Structures were solved using the SUPERFLIP package [101] and refined with full-matrix least squares on *F^2^* using the Crystals program package version 14.61 build 6236 [102]. Anisotropic displacement parameters were applied for all non-hydrogen, non-disordered atoms.

For the disordered atoms in the case of complex **2**, their occupation factors under fixed isotropic thermal parameters were first detected. Afterward, all were refined with fixed occupation factors isotropically in the case of methanol solvent molecules and anisotropically in the case of the chloride counter anion. Hydrogen atoms were, in general, found and/or positioned geometrically and refined using a riding model with the isotropic displacement parameters Uiso(H) = 1.2Ueq(C) or 1.5Ueq(methyl and –OH hydrogens) at distances of C–H 0.95 Å and O–H 0.82 Å. All methyl and OH hydrogen atoms were allowed to rotate. Hydrogen atoms riding on disordered oxygen atoms of methanol solvent molecules were positioned geometrically to fulfill hydrogen bonding demands. Details of crystal data and structure refinement parameters are shown in Table S1.

CCDC deposition numbers 2447090 and 2447091 contain the supplementary crystallographic data for complexes **1** and **2**. These data can be obtained free of charge via https://www.ccdc.cam.ac.uk/, accessed on 26 May 2025 (or from the Cambridge Crystallographic Data Centre, 12 Union Road, Cambridge CB21EZ, UK; fax: (+44) 1223-336-033; or deposit@ccdc.cam.ac.uk).

### 3.4. Study of the Biological Profile of the Complexes

The in vitro evaluation of the biological activity of the complexes, i.e., their interaction with CT DNA and albumins, was conducted following the dissolution of the complexes in DMSO (1 mM) because of their limited solubility in water. The experiments were conducted in the presence of aqueous buffer solutions, ensuring that the ratio of DMSO in the final solution did not exceed 5% (*v*/*v*). Control experiments were implemented to evaluate the impact of DMSO on the data. Minimal or no alterations were observed in the spectra of albumins or CT DNA, and appropriate adjustments were made when necessary.

All the procedures and relevant equations used in the in vitro study of the biological activity (interactions with CT DNA, pDNA, HSA, and BSA, and antioxidant activity) of the compounds are described in the Supporting Information file (Sections S1–S4).

## 4. Conclusions

Eight novel homoleptic iron(III) complexes with a series of substituted salicylaldehydes as ligands were synthesized and characterized by diverse techniques. For all complexes, the general formula [Fe(X-salo)_3_] was suggested where the X-salo ligands are bound to Fe(III) ion in a bidentate chelating fashion the aldehyde and the phenolato-oxygen atoms. The crystal structures of complexes **1** and **2** were determined by single-crystal X-ray crystallography. In the structure of complex **2**, a potassium cation was hosted in the space generated between two [Fe(ovan)_3_] moieties interacting with the methoxy and oxygen atoms of the *o*-vanillin ligands.

The complexes interacted with CT DNA in an intercalative manner, with complex **1** bearing the highest DNA-binding constant (1.62(±0.01) × 10^7^ M^−1^), which could be attributed to the extended aromatic system of the 1naph-salo ligand. The ability of the complexes to cleave pBR322 plasmid DNA to relaxed circular DNA is moderate at a concentration of 500 μM in the absence of irradiation, but it is highly enhanced upon irradiation, especially with UVA and UVB light.

The complexes may bind tightly and reversibly to bovine and human serum albumin and may become transferred to potential biological targets. Competitive studies with the reference site markers ibuprofen and warfarin showed that in the case of HSA, complexes **1**–**8** seemed to bind selectively at Sudlow’s site II. Regarding BSA, complexes **2**, **4**, and **7** prefer binding at Sudlow’s site I and complex **5** at Sudlow’s site II.

Regarding the antioxidant activity of the complexes, the ability to scavenge DPPH and ABTS radicals and to reduce H_2_O_2_ was examined. Almost all complexes were more inactive towards DPPH radicals except for complex **2**, which presented a moderate and time-dependent DPPH scavenging activity (%DPPH = 33.85 ± 0.17–49.03 ± 0.71%). The complexes showed a low-to-moderate ability to scavenge ABTS radicals, with the most active complexes **2** and **5** approaching the activity of the reference compound trolox. Concerning the activity towards H_2_O_2_, most complexes were found to be more active than the reference compound L-ascorbic acid, with complex **8** being the most active (H_2_O_2_% = 85.86 ± 0.89%).

Such features may be useful and, with the aid of more elaborate biological assays, could reveal alternative pathways regarding the potential use of these types of compounds as pharmaceutical agents.

## Figures and Tables

**Table 1:** Selected lengths of bonds (in Å) for complexes **1** and **2**.

Bond	Length (Å)	Bond	Length (Å)
Complex **1**			
Fe–O_aldehyde_		Fe–O_phenolato_	
Fe1–O1	1.976(4)	Fe1–O2	2.024(3)
Fe1–O3	1.994(4)	Fe1–O4	1.965(3)
Fe1–O5	2.042(3)	Fe1–O6	1.967(3)
Complex **2**			
Fe–O_aldehyde_		Fe–O_phenolato_	
Fe1–O1	2.0673(18)	Fe1–O2	1.9344(16)
Fe1–O4	2.0656(18)	Fe1–O5	1.9357(16)
Fe1–O7	2.0755(17)	Fe1–O8	1.9384(17)
Fe2–O10	2.049(2)	Fe2–O11	1.9346(19)
Fe2–O13	2.070(2)	Fe2–O14	1.933(2)
Fe2–O16	2.080(2)	Fe2–O17	1.942(2)
K⋯O_methoxy_		K⋯O_phenolato_	
K1⋯O3	3.101(2)	K1⋯O2	2.9761(18)
K1⋯O6	3.078 (2)	K1⋯O5	2.9155(17)
K1⋯O9	3.054(2)	K1⋯O8	2.8843(17)
K1⋯O12	3.097(2)	K1⋯O11	2.8776(19)
K1⋯O15	3.136(2)	K1⋯O14	2.8616(19)
K1⋯O18	3.227(2)	K1⋯O17	2.998(2)

**Table 2:** UV-vis spectroscopic data for the interaction of complexes **1**–**8** with CT DNA: UV-band (λ_max_, in nm) (percentage of the observed hyper-/hypo-chromism (ΔA/A_0_, in %) and blue/red shift in the λ_max_ (Δλ, in nm)); DNA-binding constant (K_b_, in M^−1^).

Compound	λ_max_ (nm) (ΔA/Aο (%)) ^a^, Δλ (nm) ^b^)	K_b_ (M^−1^)
[Fe(1naph-salo)_3_], **1**	315(−23 ^a^, −9 ^b^); 356(<− ^c^, +7); 408(>+ ^d^, +1)	1.62(±0.01) × 10^7^
{K[Fe(ovan)_3_]_2_}Cl, **2**	320(−14, +13); 354(−10, +1); 410(>+, +1)	1.65(±0.13) × 10^6^
[Fe(5Me-salo)_3_], **3**	333(−35, +5); 409(>+, +3)	9.12(±0.95) × 10^5^
[Fe(3EtO-salo)_3_], **4**	340(−24, 0); 379(>+, −5)	8.89(±0.14) × 10^4^
[Fe(4OH-salo)_3_], **5**	278(<−, −4); 315(>+, +15)	6.64(±1.06) × 10^5^
[Fe(4MeO-salo)_3_], **6**	282(−17, −9); 316(<–, +2); 386(>+, −2.5)	6.17(±1.13) × 10^5^
[Fe(4Et_2_N-salo)_3_], **7**	349(+12, −1); 407(<−, 0)	5.62(±1.09) × 10^5^
[Fe(3,5diBr-salo)_3_], **8**	319(−30, +3); 426(>+, 0)	1.05(±0.21) × 10^6^
1naph-saloH	315(−22, −9); 356(−62, +9); 409(>+, +2)	1.97(±0.10) × 10^6^
ovanH [37]	340(−28, +3); 400(sh) ^e^ (+>, 0)	9.84(±0.16) × 10^4^
5Me-saloH [37]	335(−10, 0)	1.17(±0.11) × 10^6^
3EtO-saloH	267(−25, 0); 340(−33, +4); 403(>+, +4)	1.16(±0.13) × 10^6^
4OH-saloH	282(−13, −1); 315(+24, +21)	2.38(±0.17) × 10^5^
4MeO-saloH [27]	315(−44, +1)	9.25(±0.12) × 10^5^
4Et_2_N-saloH [30]	349(−1, 0)	5.06(±0.14) × 10^5^
3,5diBr-saloH [39]	337(<−, elim ^f^); 427(>+, 0)	3.71(±0.14) × 10^5^

^a^ “+” denotes hyperchromism; “−” denotes hypochromism. ^b^ “+” denotes red shift; “−” denotes blue shift. ^c^ “<−” denotes intense hypochromism. ^d^ “>+” denotes intense hyperchromism. ^e^ “(sh)” = (shoulder). ^f^ “elim” = eliminated.

**Table 3:** Fluorescence features of the EB displacement studies for complexes **1**–**8**: the percentage of EB-DNA fluorescence emission quenching (ΔI/I_0_, in %), Stern-Volmer (K_SV_, in M^−1^), and quenching constants (K_q_, in M^−1^ s^−1^).

Compound	ΔΙ/Ι_ο_ (%)	K_sv_ (M^−1^)	K_q_ (M^−1^ s^−1^)
[Fe(1naph-salo)_3_], **1**	58.3	7.11(±0.15) × 10^4^	3.09(±0.07) × 10^12^
{K[Fe(ovan)_3_]_2_}Cl, **2**	62.6	1.01(±0.03) × 10^5^	4.38(±0.14) × 10^12^
[Fe(5Me-salo)_3_], **3**	60.9	6.99(±0.22) × 10^4^	3.04(±0.10) × 10^12^
[Fe(3EtO-salo)_3_], **4**	59.3	1.35(±0.02) × 10^5^	5.88(±0.08) × 10^12^
[Fe(4OH-salo)_3_], **5**	78.8	8.58(±0.35) × 10^4^	3.73(±0.15) × 10^12^
[Fe(4MeO-salo)_3_], **6**	65.8	9.17(±0.13) × 10^4^	3.99(±0.06) × 10^12^
[Fe(4Et_2_N-salo)_3_], **7**	72.7	1.17(±0.06) × 10^5^	5.08(±0.25) × 10^12^
[Fe(3,5diBr-salo)_3_], **8**	79.1	3.88(±0.14) × 10^4^	1.69(±0.62) × 10^12^

**Table 4:** The SA-quenching constants (K_q_, in M^−1^ s^−1^) and the SA-binding constants in the absence or presence of the albumin site markers ibuprofen and warfarin (K, in M^−1^) calculated for complexes **1**–**8**.

**Compound**	**K_q(BSA)_ (M^−1^ s^−1^)**	**K_(BSA)_ (M^−1^)**	**K_(BSA,ibuprofen)_ (M^−1^)**	**K_(BSA,warfarin)_ (M^−1^)**
[Fe(1naph-salo)_3_], **1**	8.94(±0.28) × 10^12^	4.42(±0.26) × 10^5^	8.57(±0.24) × 10^4^	8.73(±0.42) × 10^4^
{K[Fe(ovan)_3_]_2_}Cl, **2**	9.28(±0.38) × 10^12^	6.07(±0.34) × 10^4^	1.15(±0.03) × 10^5^	2.57(±0.16) × 10^4^
[Fe(5Me-salo)_3_], **3**	2.78(±0.05) × 10^12^	3.39(±0.25) × 10^4^	5.99(±0.20) × 10^4^	5.38(±0.22) × 10^4^
[Fe(3EtO-salo)_3_], **4**	5.41(±0.17) × 10^12^	1.15(±0.04) × 10^5^	7.10(±0.16) × 10^4^	3.75(±0.19) × 10^4^
[Fe(4OH-salo)_3_], **5**	1.81(±0.10) × 10^13^	8.63(±0.50) × 10^4^	5.20(±0.23) × 10^4^	1.47(±0.11) × 10^5^
[Fe(4MeO-salo)_3_], **6**	6.03(±0.24) × 10^12^	7.02(±0.54) × 10^3^	2.15(±0.11) × 10^4^	2.85(±0.23) × 10^4^
[Fe(4Et_2_N-salo)_3_], **7**	1.66(±0.05) × 10^13^	8.61(±0.44) × 10^4^	9.82(±0.32) × 10^4^	2.48(±0.10) × 10^4^
[Fe(3,5diBr-salo)_3_], **8**	2.54(±0.09) × 10^13^	1.21(±0.04) × 10^6^	1.31(±0.10) × 10^6^	1.33(±0.08) × 10^6^
**Compound**	**K_q(HSA)_ (M^−1^ s^−1^)**	**K_(HSA)_ (M^−1^)**	**K_(HSA,ibuprofen)_ (M^−1^)**	**K_(HSA,warfarin)_ (M^−1^)**
[Fe(1naph-salo)_3_], **1**	4.22(±0.14) × 10^12^	9.99(±0.23) × 10^5^	7.13(±0.23) × 10^4^	1.44(±0.05) × 10^5^
{K[Fe(ovan)_3_]_2_}Cl, **2**	8.23(±0.49) × 10^12^	3.67(±0.25) × 10^4^	5.11(±0.13) × 10^4^	2.17(±0.06) × 10^5^
[Fe(5Me-salo)_3_], **3**	1.22(±0.06) × 10^12^	2.17(±0.12) × 10^5^	4.56(±0.24) × 10^4^	1.98(±0.07) × 10^5^
[Fe(3EtO-salo)_3_], **4**	2.47(±0.12) × 10^12^	1.45(±0.07) × 10^5^	5.11(±0.20) × 10^4^	2.13(±0.07) × 10^5^
[Fe(4OH-salo)_3_], **5**	2.08(±0.13) × 10^13^	8.03(±0.23) × 10^4^	5.46(±0.29) × 10^4^	1.83(±0.06) × 10^5^
[Fe(4MeO-salo)_3_], **6**	4.23(±0.15) × 10^12^	8.48(±0.55) × 10^4^	1.82(±0.02) × 10^4^	2.01(±0.05) × 10^5^
[Fe(4Et_2_N-salo)_3_], **7**	1.09(±0.03) × 10^13^	1.52(±0.07) × 10^5^	5.20(±0.05) × 10^4^	3.29(±0.10) × 10^5^
[Fe(3,5diBr-salo)_3_], **8**	2.01(±0.05) × 10^13^	1.99(±0.09) × 10^6^	1.24(±0.05) × 10^6^	1.71(±0.14) × 10^6^

**Table 5:** %DPPH scavenging ability (DPPH%), %ABTS scavenging activity (ABTS %), and H_2_O_2_-reducing ability (H_2_O_2_%) for the compounds.

Compound	DPPH% (30 min/60 min)	ABTS%	%H_2_O_2_
[Fe(1naph-salo)_3_], **1**	8.60 ± 0.62/6.46 ± 0.89	17.78 ± 0.53	68.69 ± 0.05
{K[Fe(ovan)_3_]_2_}Cl, **2**	33.85 ± 0.17/49.03 ± 0.71	85.69 ± 0.67	54.30 ± 0.48
[Fe(5Me-salo)_3_], **3**	11.91 ± 0.11/11.18 ± 0.67	31.29 ± 0.62	44.04 ± 0.36
[Fe(3EtO-salo)_3_], **4**	23.70 ± 0.42/29.40 ± 0.71	78.62 ± 0.71	71.10 ± 0.06
[Fe(4OH-salo)_3_], **5**	0.86 ± 0.34/2.62 ± 0.45	84.72 ± 0.76	72.28 ± 0.19
[Fe(4MeO-salo)_3_], **6**	4.83 ± 0.93/5.43 ± 0.27	11.05 ± 0.59	47.76 ± 0.61
[Fe(4Et_2_N-salo)_3_], **7**	Not active	57.07 ± 0.67	49.46 ± 0.51
[Fe(3,5diBr-salo)_3_], **8**	Not active	60.39 ± 0.53	85.86 ± 0.89
BHT	70.23 ± 0.95/88.60 ± 0.27	Not tested	Not tested
NDGA	93.51 ± 0.12/93.54 ± 0.11	Not tested	Not tested
Trolox	Not tested	89.25 ± 0.11	Not tested
L-ascorbic acid	Not tested	Not tested	60.51 ± 0.54

## Data Availability

All necessary supplementary data are included in the supplementary files.

## Supplementary Materials

The following supporting information can be downloaded at https://www.mdpi.com/article/10.3390/molecules30112383/s1. Cif and checkcif files for compounds **1** and **2**. Protocols and equations regarding binding studies with CT DNA (S1), pDNA cleavage studies (S2), albumin-binding studies (S3), and antioxidant activity assay (S4) [103,104,105]. Tables S1–S4 and Figures S1–S25 are included in the ESI file.

## Abbreviations

The following abbreviations are used in this manuscript:

1naph-saloH	2-hydroxy-1-naphthaldehyde
3,5diBr-saloH	3,5-dibromo-salicylaldehyde
3EtO-saloH	3-ethoxy-salicylaldehyde
4Et2N-saloH	4-diethylamino-salicylaldehyde
4MeO-saloH	4-methoxy-salicylaldehyde
4OH-saloH	4-hydroxy-salicylaldehyde
5Me-saloH	5-methyl-salicylaldehyde
ABTS	2,2′-azinobis-(3-ethylbenzothiazoline-6-sulfonic acid)
BHT	Butylated hydroxytoluene
BSA	Bovine serum albumin
CT	Calf thymus
DPPH	1,1-diphenyl-picrylhydrazyl
EB	Ethidium bromide
HSA	Human serum albumin
K	SA-binding constant
Kb	DNA-binding constant
Kq	Quenching constant
KSV	Stern-Volmer constant
NDGA	Nordihydroguaiaretic acid
ovan	3-methoxy-salicylaldehyde, o-vanillin
pDNA	pBR322 plasmid DNA
SA	Serum albumin
saloH	Salicylaldehyde
trolox	6-hydroxy-2,5,7,8-tetramethylchroman-2-carboxylic acid
X-saloH	Substituted salicylaldehyde
