# Supplementary material for: Iron(III) Complexes with Substituted Salicylaldehydes: Synthesis, Interaction with DNA and Serum Albumins, and Antioxidant Activity
Source: Molecules. 2025 May 29;30(11):2383. doi: 10.3390/molecules30112383 (PMC12156386; doi:10.3390/molecules30112383)

checkCIF/PLATON report

Structure factors have been supplied for datablock(s) I

THIS REPORT IS FOR GUIDANCE ONLY. IF USED AS PART OF A REVIEW PROCEDURE FOR PUBLICATION, IT SHOULD NOT REPLACE THE EXPERTISE OF AN EXPERIENCED CRYSTALLOGRAPHIC REFEREE.

No syntax errors found.   CIF dictionary   Interpreting this report

Datablock: I

```
Bond precision:   C-C = 0.0085 A           Wavelength=0.71073

Cell:            a=19.439(3)    b=24.178(3)         c=13.7640(19)
                 alpha=90       beta=127.213(3)     gamma=90
Temperature:     295 K
```

	Calculated	Reported
Volume	5151.9(13)	5151.8(12)
Space group	C 2/c	C 1 2/c 1
Hall group	-C 2yc	?
Moiety formula	C33 H21 Fe O6	C33 H21 Fe1 O6
Sum formula	C33 H21 Fe O6	C33 H21 Fe1 O6
Mr	569.35	569.37
Dx,g cm-3	1.468	1.468
Z	8	8
Mu (mm-1)	0.633	0.633
F000	2344.0	2344.0
F000'	2347.84	
h,k,lmax	23,29,16	23,29,16
Nref	4800	4707
Tmin,Tmax	0.920,0.975	0.930,0.970
Tmin'	0.892	

```
Correction method= # Reported T Limits: Tmin=0.930 Tmax=0.970
AbsCorr = NUMERICAL

Data completeness= 0.981            Theta(max)= 25.499

R(reflections)= 0.0564( 3152)       wR2(reflections)= 0.1063( 3152)

S = 1.000                  Npar= 361
```

The following ALERTS were generated. Each ALERT has the format
test-name_ALERT_alert-type_alert-level.
Click on the hyperlinks for more details of the test.

Alert level C

```
PLAT041_ALERT_1_C Calc. and Reported SumFormula    Strings  Differ     Please Check
              Calc: C33 H21 Fe O6
              Rep.: C33 H21 Fe1 O6
PLAT042_ALERT_1_C Calc. and Reported MoietyFormula Strings  Differ     Please Check
              Calc: C33 H21 Fe O6
              Rep.: C33 H21 Fe1 O6
PLAT250_ALERT_2_C Large U3/U1 Ratio for <U(i,j)> Tensor(Resd    1)        2.3 Note
PLAT341_ALERT_3_C Low Bond Precision on  C-C Bonds ..............    0.00847 Ang.
PLAT911_ALERT_3_C Missing FCF Refl Between Thmin & STh/L=    0.600         74 Report
                2  0  0,   3  1  0,   2  2  0,   1  3  0,   0  4  0,   0 26  0,
                1 27  0,   0 28  0,   2 28  0,  -3  1  1,   1  1  1,  -3  3  1,
               -1  3  1,   1  3  1,  -2  4  1,   0  4  1,  -2 26  1,   2 26  1,
               -3 27  1,  -1 27  1,  -4 28  1,  -2 28  1,   0 28  1,  -2  0  2,
                0  0  2,  -3  1  2,  -1  1  2,  -2  2  2,  -1  3  2,  -4 26  2,
               -7 27  2,  -5 27  2,  -3 27  2,  -6 28  2,  -4 28  2,  -2 28  2,
               -6 26  3,  -7 27  3,  -5 27  3,  -6 28  3,  -4 28  3,  -4  0  4,
               -9 25  4,  -8 26  4,  -6 26  4,  -7 27  4,  -5 27  4, -10 22  5,
              -11 23  5, -11 25  5,  -9 25  5,  -8 26  5, -22  6  6, -13 23  6,
              -23  1  8, -12 24  8, -23  3  9, -16 14 10, -18 12 11, -18 14 11,
              -17 15 11, -18 16 11, -17 17 11, -18 10 12, -19 13 12, -17 13 12,
              -18 14 12, -17 15 12, -17 13 13, -18 14 13, -17 15 13, -18  0 16,
              -18  2 16, -16  6 16,
```

Alert level G

```
PLAT128_ALERT_4_G Alternate Setting for Input Space Group     C2/c       I2/a Note
PLAT769_ALERT_4_G CIF Embedded Explicitly Supplied Scattering Data     Please Note
PLAT794_ALERT_5_G Tentative Bond Valency for Fe1       (III)     .      3.18 Info
PLAT808_ALERT_5_G No Parseable SHELXL Style Weighting Scheme Found     Please Check
PLAT882_ALERT_1_G No Datum for _diffrn_reflns_av_unetI/netI ......     Please Do !
PLAT912_ALERT_4_G Missing # of FCF Reflections Above STh/L=  0.600         35 Note
PLAT913_ALERT_3_G Missing # of Very Strong Reflections in FCF ....          2 Note
               -3  1  1,  -4  0  4,
PLAT929_ALERT_5_G No Weight Pars,Obs and Calc R1,wR2,S not Checked          ! Info
PLAT940_ALERT_3_G Fsqd Refinement With I > n * Sigma(I) Only .....     Please Check
PLAT960_ALERT_3_G Number of Intensities with I < - 2*Sigma(I) ....         30 Check
PLAT969_ALERT_5_G The 'Henn et al.' R-Factor-gap value ..........      6.824 Note
              Predicted wR2: Based on SigI**2  1.43 or SHELX Weight 12.11
```

```
   0 ALERT level A = Most likely a serious problem - resolve or explain
   0 ALERT level B = A potentially serious problem, consider carefully
   5 ALERT level C = Check. Ensure it is not caused by an omission or oversight
  11 ALERT level G = General information/check it is not something unexpected

   3 ALERT type 1 CIF construction/syntax error, inconsistent or missing data
   1 ALERT type 2 Indicator that the structure model may be wrong or deficient
   5 ALERT type 3 Indicator that the structure quality may be low
   3 ALERT type 4 Improvement, methodology, query or suggestion
   4 ALERT type 5 Informative message, check
```

It is advisable to attempt to resolve as many as possible of the alerts in all categories. Often the minor alerts point to easily fixed oversights, errors and omissions in your CIF or refinement strategy, so attention to these fine details can be worthwhile. In order to resolve some of the more serious problems it may be necessary to carry out additional measurements or structure refinements. However, the purpose of your study may justify the reported deviations and the more serious of these should normally be commented upon in the discussion or experimental section of a paper or in the "special_details" fields of the CIF. checkCIF was carefully designed to identify outliers and unusual parameters, but every test has its limitations and alerts that are not important in a particular case may appear. Conversely, the absence of alerts does not guarantee there are no aspects of the results needing attention. It is up to the individual to critically assess their own results and, if necessary, seek expert advice.

Publication of your CIF in IUCr journals

A basic structural check has been run on your CIF. These basic checks will be run on all CIFs submitted for publication in IUCr journals (*Acta Crystallographica*, *Journal of Applied Crystallography*, *Journal of Synchrotron Radiation*); however, if you intend to submit to *Acta Crystallographica Section C* or *E* or *IUCrData*, you should make sure that full publication checks are run on the final version of your CIF prior to submission.

Publication of your CIF in other journals

Please refer to the *Notes for Authors* of the relevant journal for any special instructions relating to CIF submission.

PLATON version of 02/02/2025; check.def file version of 02/02/2025

Datablock I - ellipsoid plot

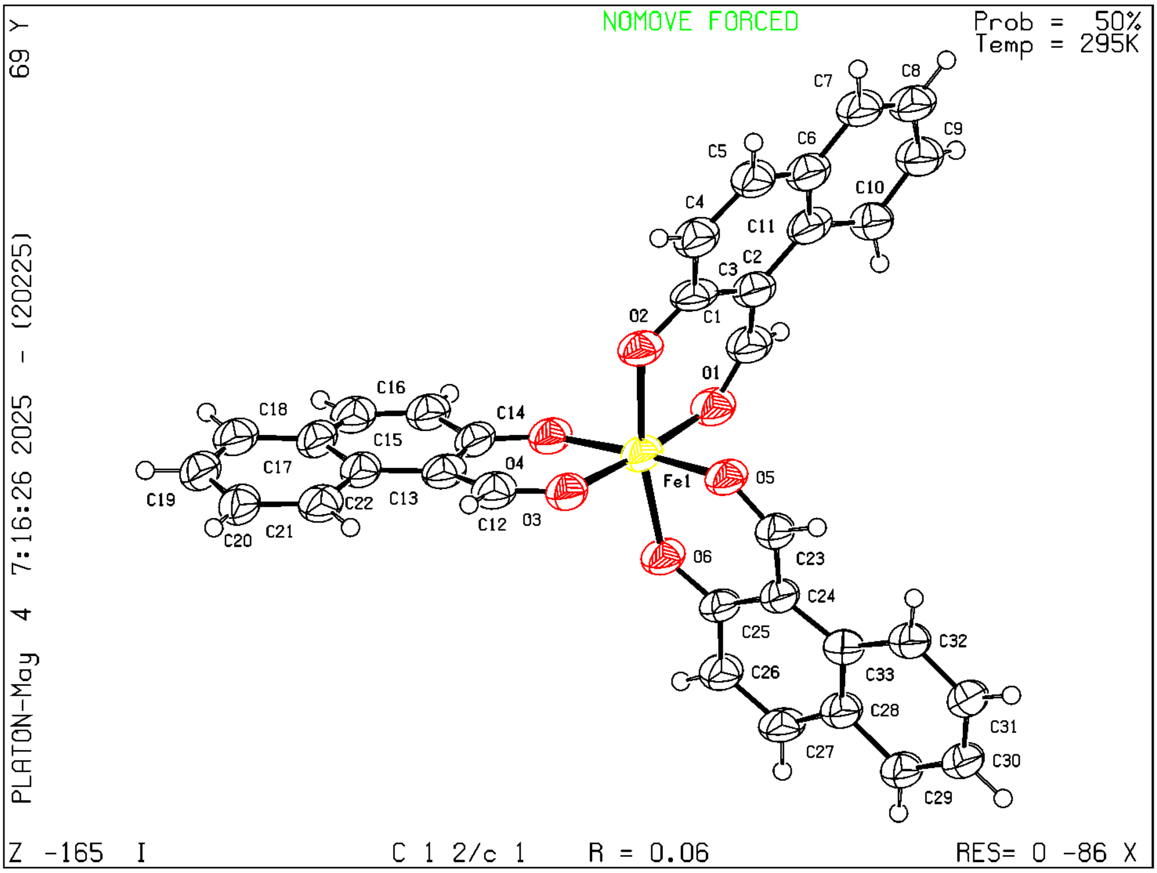

Supplement: Supplementary file 1 [file molecules-30-02383-s001.zip › Checkcif of complex 1.pdf]
