# Supplementary material for: Iron(III) Complexes with Substituted Salicylaldehydes: Synthesis, Interaction with DNA and Serum Albumins, and Antioxidant Activity
Source: Molecules. 2025 May 29;30(11):2383. doi: 10.3390/molecules30112383 (PMC12156386; doi:10.3390/molecules30112383)

checkCIF/PLATON report

Structure factors have been supplied for datablock(s) I

THIS REPORT IS FOR GUIDANCE ONLY. IF USED AS PART OF A REVIEW PROCEDURE FOR PUBLICATION, IT SHOULD NOT REPLACE THE EXPERTISE OF AN EXPERIENCED CRYSTALLOGRAPHIC REFEREE.

No syntax errors found. CIF dictionary Interpreting this report

Datablock: I

Bond precision: C-C = 0.0046 A Wavelength=0.71073

Cell:	a=12.9316(9)	b=13.1757(10)	c=15.9281(11)
	alpha=106.397(2)	beta=92.980(2)	gamma=94.284(2)
Temperature:	295 K		

	Calculated	Reported
Volume	2588.6(3)	2588.6(3)
Space group	P -1	P -1
Hall group	-P 1	?
Moiety formula	C48 H42 Fe2 K O18, C H4 O, Cl	C49 H46 Cl1 Fe2 K1 O19
Sum formula	C49 H46 Cl Fe2 K O19	C49 H46 Cl1 Fe2 K1 O19
Mr	1125.11	1125.14
Dx,g cm-3	1.444	1.443
Z	2	2
Mu (mm-1)	0.766	0.766
F000	1160.0	1160.0
F000'	1162.67	
h,k,lmax	15,16,19	15,16,19
Nref	9944	9773
Tmin,Tmax	0.817,0.933	0.840,0.930
Tmin'	0.813	

Correction method= # Reported T Limits: Tmin=0.840 Tmax=0.930
AbsCorr = NUMERICAL

Data completeness= 0.983 Theta(max)= 25.772

R(reflections)= 0.0464(8106) wR2(reflections)= 0.0824(8106)

S = 1.000 Npar= 656

The following ALERTS were generated. Each ALERT has the format
test-name_ALERT_alert-type_alert-level.
Click on the hyperlinks for more details of the test.

Alert level C

```
PLAT041_ALERT_1_C Calc. and Reported SumFormula    Strings  Differ     Please Check
              Calc: C49 H46 Cl Fe2 K O19
              Rep.: C49 H46 Cl1 Fe2 K1 O19
PLAT042_ALERT_1_C Calc. and Reported MoietyFormula Strings  Differ     Please Check
              Calc: C48 H42 Fe2 K O18, C H4 O, Cl
              Rep.: C49 H46 Cl1 Fe2 K1 O19
PLAT202_ALERT_3_C Isotropic non-H Atoms in Anion/Solvent .........          2 Check
              O19     C49
PLAT911_ALERT_3_C Missing FCF Refl Between Thmin & STh/L=    0.600         44 Report
               -1  1  0,  -1  2  0,   0  2  0,   0 10  0,   0 -2  1,  -1 -1  1,
                0 -1  1,   1 -1  1,  -2  0  1,  -1  0  1,   1  0  1,  -1  1  1,
                0  1  1,   1  1  1,  -1  3  1, -13  9  1,  -4-11  2,  -2 -1  2,
               -1 -1  2,   0 -1  2,   1 -1  2,  -1  0  2,   0  0  2,   1  0  2,
               -1  2  2,  -6-10  3,  -2 -3  3,   1 -1  3,  -6-10  4,  -6 -8  4,
              -15 -2  4,  -7 -7  5, -15 -1  5,   1 12  6,   1 11  7,   2 11  7,
                1 12  7,   2 12  7,   1 11  8,   2 11  8,  -3 11  9,  -2 11  9,
                1 11  9,   2  2 17,
PLAT913_ALERT_3_C Missing # of Very Strong Reflections in FCF ....          4 Note
               -1  3  1,   0  0  2,  -1  2  2,  -2 -3  3,
```

Alert level G

```
PLAT002_ALERT_2_G Number of Distance or Angle Restraints on AtSite          4 Note
PLAT007_ALERT_5_G Number of Unrefined Donor-H Atoms .............           2 Report
              H191  H201
PLAT154_ALERT_1_G The s.u.'s on the Cell Angles are Equal ..(Note)      0.002 Degree
PLAT300_ALERT_4_G Atom Site Occupancy of O19        Constrained at       0.75 Check
PLAT300_ALERT_4_G Atom Site Occupancy of C49        Constrained at       0.75 Check
PLAT300_ALERT_4_G Atom Site Occupancy of H191       Constrained at       0.75 Check
PLAT300_ALERT_4_G Atom Site Occupancy of H491       Constrained at       0.75 Check
PLAT300_ALERT_4_G Atom Site Occupancy of H492       Constrained at       0.75 Check
PLAT300_ALERT_4_G Atom Site Occupancy of H493       Constrained at       0.75 Check
PLAT300_ALERT_4_G Atom Site Occupancy of O20        Constrained at       0.25 Check
PLAT300_ALERT_4_G Atom Site Occupancy of C50        Constrained at       0.25 Check
PLAT300_ALERT_4_G Atom Site Occupancy of H201       Constrained at       0.25 Check
PLAT300_ALERT_4_G Atom Site Occupancy of H501       Constrained at       0.25 Check
PLAT300_ALERT_4_G Atom Site Occupancy of H502       Constrained at       0.25 Check
PLAT300_ALERT_4_G Atom Site Occupancy of H503       Constrained at       0.25 Check
PLAT300_ALERT_4_G Atom Site Occupancy of Cl1        Constrained at       0.75 Check
PLAT300_ALERT_4_G Atom Site Occupancy of Cl2        Constrained at       0.25 Check
PLAT302_ALERT_4_G Anion/Solvent/Minor-Residue Disorder (Resd    2)       100% Note
PLAT302_ALERT_4_G Anion/Solvent/Minor-Residue Disorder (Resd    3)       100% Note
PLAT302_ALERT_4_G Anion/Solvent/Minor-Residue Disorder (Resd    4)       100% Note
PLAT302_ALERT_4_G Anion/Solvent/Minor-Residue Disorder (Resd    5)       100% Note
PLAT304_ALERT_4_G Non-Integer Number of Atoms in ..... (Resd    2)       4.50 Check
PLAT304_ALERT_4_G Non-Integer Number of Atoms in ..... (Resd    3)       1.50 Check
PLAT304_ALERT_4_G Non-Integer Number of Atoms in ..... (Resd    4)       0.75 Check
PLAT304_ALERT_4_G Non-Integer Number of Atoms in ..... (Resd    5)       0.25 Check
PLAT413_ALERT_2_G Short Inter XH3 .. XHn     H71      ..H501     .       2.04 Ang.
                                                  1-x,1-y,-z  =      2_665 Check
```

```
PLAT432_ALERT_2_G Short Inter X...Y Contact  C12      ..C50      .       2.83 Ang.
                                                  x,1+y,z  =     1_565 Check
PLAT432_ALERT_2_G Short Inter X...Y Contact  C40      ..C50      .       3.14 Ang.
                                                    x,y,z  =     1_555 Check
PLAT769_ALERT_4_G CIF Embedded Explicitly Supplied Scattering Data     Please Note
PLAT794_ALERT_5_G Tentative Bond Valency for Fe1       (III)     .       3.15 Info
PLAT794_ALERT_5_G Tentative Bond Valency for Fe2       (III)     .       3.16 Info
PLAT808_ALERT_5_G No Parseable SHELXL Style Weighting Scheme Found     Please Check
PLAT860_ALERT_3_G Number of Least-Squares Restraints ............          2 Note
PLAT882_ALERT_1_G No Datum for _diffrn_reflns_av_unetI/netI ......     Please Do !
PLAT910_ALERT_3_G Missing # of FCF Reflection(s) Below Theta(Min).          1 Note
                0  0  1,
PLAT912_ALERT_4_G Missing # of FCF Reflections Above STh/L=  0.600        134 Note
PLAT929_ALERT_5_G No Weight Pars,Obs and Calc R1,wR2,S not Checked          ! Info
PLAT960_ALERT_3_G Number of Intensities with I < - 2*Sigma(I) ....        306 Check
PLAT969_ALERT_5_G The 'Henn et al.' R-Factor-gap value ..........      7.956 Note
              Predicted wR2: Based on SigI**2  0.94 or SHELX Weight  8.28
PLAT992_ALERT_5_G Repd & Actual _reflns_number_gt Values Differ by          3 Check
```

---

0 **ALERT level A** = Most likely a serious problem - resolve or explain
0 **ALERT level B** = A potentially serious problem, consider carefully
5 **ALERT level C** = Check. Ensure it is not caused by an omission or oversight
40 **ALERT level G** = General information/check it is not something unexpected

4 ALERT type 1 CIF construction/syntax error, inconsistent or missing data
4 ALERT type 2 Indicator that the structure model may be wrong or deficient
6 ALERT type 3 Indicator that the structure quality may be low
24 ALERT type 4 Improvement, methodology, query or suggestion
7 ALERT type 5 Informative message, check

It is advisable to attempt to resolve as many as possible of the alerts in all categories. Often the minor alerts point to easily fixed oversights, errors and omissions in your CIF or refinement strategy, so attention to these fine details can be worthwhile. In order to resolve some of the more serious problems it may be necessary to carry out additional measurements or structure refinements. However, the purpose of your study may justify the reported deviations and the more serious of these should normally be commented upon in the discussion or experimental section of a paper or in the "special_details" fields of the CIF. checkCIF was carefully designed to identify outliers and unusual parameters, but every test has its limitations and alerts that are not important in a particular case may appear. Conversely, the absence of alerts does not guarantee there are no aspects of the results needing attention. It is up to the individual to critically assess their own results and, if necessary, seek expert advice.

Publication of your CIF in IUCr journals

A basic structural check has been run on your CIF. These basic checks will be run on all CIFs submitted for publication in IUCr journals (*Acta Crystallographica*, *Journal of Applied Crystallography*, *Journal of Synchrotron Radiation*); however, if you intend to submit to *Acta Crystallographica Section C* or *E* or *IUCrData*, you should make sure that full publication checks are run on the final version of your CIF prior to submission.

Publication of your CIF in other journals

Please refer to the *Notes for Authors* of the relevant journal for any special instructions relating to CIF submission.

PLATON version of 02/02/2025; check.def file version of 02/02/2025

Datablock I - ellipsoid plot

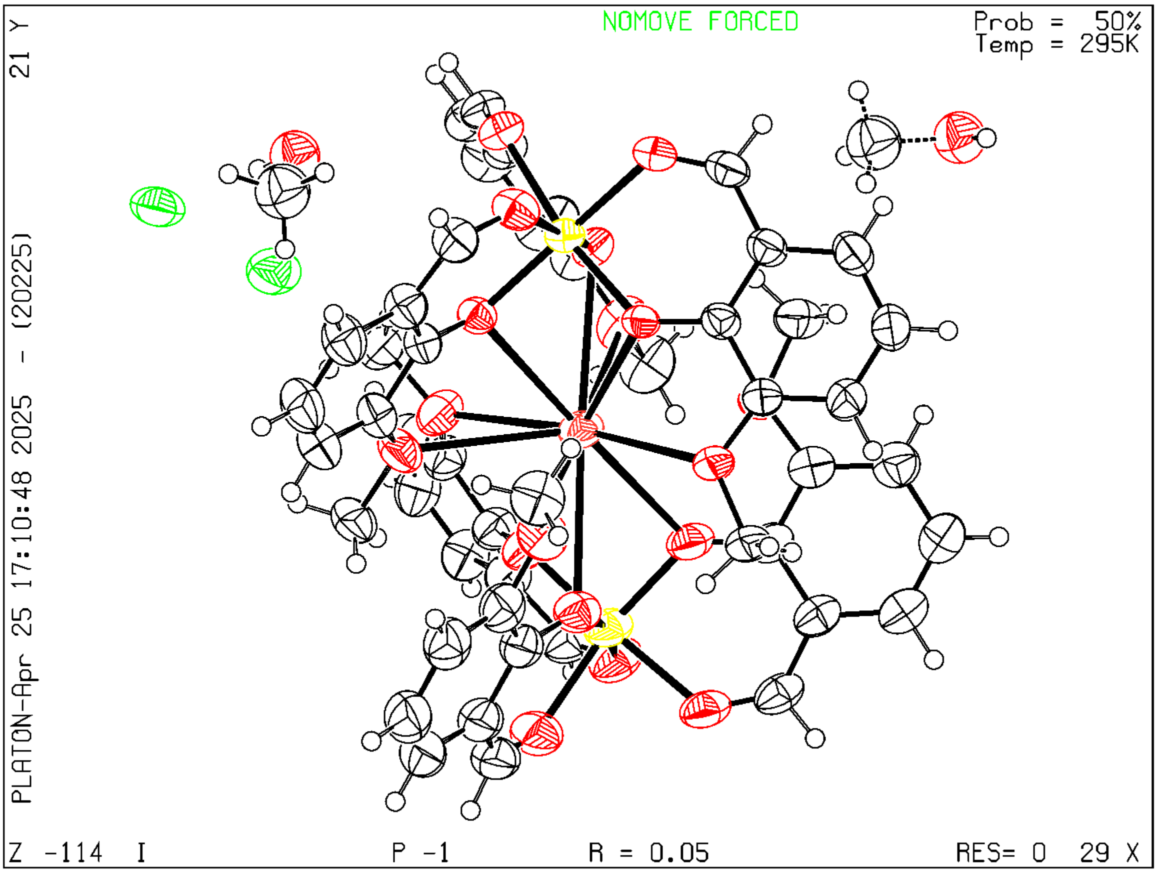

Supplement: Supplementary file 1 [file molecules-30-02383-s001.zip › Checkcif of complex 2.pdf]
