# Supplementary material for: Iron(III) Complexes with Substituted Salicylaldehydes: Synthesis, Interaction with DNA and Serum Albumins, and Antioxidant Activity
Source: Molecules. 2025 May 29;30(11):2383. doi: 10.3390/molecules30112383 (PMC12156386; doi:10.3390/molecules30112383)
Supplement: Supplementary file 1 [file molecules-30-02383-s001.zip › molecules-3658998, ESI revised.pdf]

Iron(III) complexes with substituted salicylaldehydes: Synthesis, interaction with DNA and serum albumins, and antioxidant activity

Zisis Papadopoulos, Antonios G. Hatzidimitriou, George Psomas*

Laboratory of Inorganic Chemistry, Department of Chemistry, Aristotle University of Thessaloniki, GR-54124 Thessaloniki, Greece

SUPPLEMENTARY MATERIAL

* Corresponding author e–mail: gepsomas@chem.auth.gr (G. Psomas)

Content

EXPERIMENTAL PROTOCOLS 4

S1 Binding studies with CT DNA 4

S1.1 Binding study with CT DNA by UV−vis spectroscopy 4

S1.2 CT DNA−binding studies by viscosity measurements 4

S1.3 EB−displacement studies 4

S1.4 Determination of the thermodynamic parameters 5

S2 Plasmid DNA cleavage experiments 5

S3 Albumin−binding studies 6

S3.1 Interaction with albumins 6

S3.2 Competitive albumin−fluorescence studies with warfarin and ibuprofen 6

S4 Antioxidant activity assay 7

S4.1 Determination of the reducing activity of the radical DPPH 7

S4.2 Assay of radical cation ABTS−scavenging activity 7

S4.3 Reduction of hydrogen peroxide 7

S5 References 8

TABLES 9

Table S1. Experimental crystallographic details for complexes **1** and **2**. 9

Table S2. Selected bonds lengths (in Å) and angles (in °) for complex **1**. 10

Table S3. Selected bonds lengths (in Å) and angles (in °) for complex **2**. 11

Table S4. Thermodynamic parameters of the compounds for the interaction with CT DNA at different temperatures (295 K, 303 K and 310 K). 13

FIGURES 14

Figure S1. IR spectra (ATR) of the complexes. 14

Figure S2: Proposed structures for complexes **3-8.** 16

Figure S3. UV−vis spectra of a DMSO solution of complexes **1-8**, in the presence of increasing amounts of CT DNA 17

Figure S4. Plots of $[\text{DNA}](\varepsilon A - \varepsilon f)$ *versus* [DNA] for complexes **1-8**. 18

Figure S5. Fluorescence emission spectra for EB-DNA in buffer solution in the absence and presence of increasing amounts of the compounds. 19

Figure S6. Stern−Volmer plots of the EB−DNA quenching experiments upon addition of the compounds. 20

Figure S7. van't Hoff plots for the interaction of CT DNA with the compounds. 21

Figure S8: Agarose gel electrophoretic pattern of EB-stained plasmid DNA (pBR322 plasmid DNA) after incubation with the compounds, in dark. 22

Figure S9: Agarose gel electrophoretic pattern of EB-stained plasmid DNA (pBR322 plasmid DNA) with the compounds, after irradiation at 312 nm (UV-B) for 30 min. 23

Figure S10: Agarose gel electrophoretic pattern of EB-stained plasmid DNA (pBR322 plasmid DNA) with the compounds, after irradiation at 365 nm (UV-A) for 30 min. 24

Figure S11: Agarose gel electrophoretic pattern of EB-stained plasmid DNA (pBR322 plasmid DNA) with the compounds, after irradiation under visible light for 2 h....................................... 25

Figure S12. Fluorescence emission spectra of BSA in buffer solution in the presence of increasing amounts of the compounds. 26

Figure S13. Fluorescence emission spectra of HSA in buffer solution in the presence of increasing amounts of the compounds. 27

Figure S14. Stern−Volmer plots of the BSA-quenching experiments upon addition of the compounds. 28

Figure S15. Stern−Volmer plots of the HSA-quenching experiments upon addition of the compounds. 29

Figure S16. Scatchard plots of the BSA-quenching experiments upon addition of the compounds. 30

Figure S17. Scatchard plots of the HSA-quenching experiments upon addition of the compounds. 31

Figure S18. Fluorescence emission spectra of BSA in buffer solution in the presence of warfarin upon addition of increasing amounts of the compounds. 32

Figure S19. Fluorescence emission spectra of BSA in buffer solution in the presence of ibuprofen upon addition of increasing amounts of the compounds. 33

Figure S20. Fluorescence emission spectra of HSA in buffer solution in the presence of warfarin upon addition of increasing amounts of the compounds. 34

Figure S21. Fluorescence emission spectra of HSA in buffer solution in the presence of ibuprofen upon addition of increasing amounts of the compounds. 35

Figure S22. Scatchard plots of the BSA quenching experiments in the presence of warfarin upon addition of the compounds.. 36

Figure S23. Scatchard plots of the BSA quenching experiments in the presence of ibuprofen upon addition of the compounds. 37

Figure S24. Scatchard plots of the HSA quenching experiments in the presence of warfarin upon addition of the compounds.. 38

Figure S25. Scatchard plots of the HSA quenching experiments in the presence of ibuprofen upon addition of the compounds. 39

EXPERIMENTAL PROTOCOLS

S1 Binding studies with CT DNA

In order to study the interaction of the compounds with DNA, the compound was initially dissolved in DMSO (1 mM). Mixing of such solutions with the aqueous buffer solutions of DNA used in the studies never exceeded 5% DMSO (v/v) in the final solution, which was needed due to low aqueous solubility of most compounds. In all experiments, the effect of DMSO on the data has been taken into consideration and the appropriate corrections have been performed. The interaction of the compounds with CT DNA was monitored by UV−vis spectroscopy, and viscosity measurements, and *via* competitive studies with EB by fluorescence emission spectroscopy.

S1.1 Binding study with CT DNA by UV−vis spectroscopy

The interaction of the compounds with CT DNA has been studied by UV−vis spectroscopy in order to investigate the possible binding mode to CT DNA and to calculate the DNA−binding constant (K_b). The K_b constant (in M^{-1}) of the compounds was determined with the Wolfe−Shimer equation (equation S1) [1] and the plots [DNA]/(ε_A−ε_f) *versus* [DNA] using the UV−vis spectra of the compounds (40-100 μM) recorded for a constant concentration with increasing amounts of CT DNA for diverse [compound]/[DNA] mixing ratios (= *r*). According to the Wolfe−Shimer equation:

$$\frac{[\mathrm{DNA}]}{(\varepsilon_A-\varepsilon_f)}=\frac{[\mathrm{DNA}]}{(\varepsilon_b-\varepsilon_f)}+\frac{1}{K_b(\varepsilon_b-\varepsilon_f)} \qquad \text{(equation S1)}$$

where [DNA] is the concentration of DNA in base pairs, $\varepsilon_A = A_{obsd}$/[compound], ε_f = the extinction coefficient for the free compound and ε_b = the extinction coefficient for the compound in the fully bound form. K_b is given by the ratio of slope to the y intercept in plots [DNA]/(ε_A−ε_f) *versus* [DNA].

S1.2 CT DNA−binding studies by viscosity measurements

The interaction of compounds with DNA was evaluated *via* the study of the CT DNA viscosity ([DNA] = 0.1 mM) in a buffer solution (150 mM NaCl and 15 mM trisodium citrate at pH 7.0) in the presence of increasing amounts of the compounds (up to the value of *r* = 0.32). The obtained data are presented as $(\eta/\eta_0)^{1/3}$ *versus r*, where η is the viscosity of DNA in the presence of the compound, and η_0 is the viscosity of DNA alone in buffer solution.

S1.3 EB−displacement studies

The competition of the compounds with EB was investigated by fluorescence emission spectroscopy to examine whether the compounds can displace EB from its DNA−EB adduct. The CT DNA−EB complex was formed by pre−treating 40 μM EB and 46 μM CT DNA in buffer (150 mM NaCl and 15 mM trisodium citrate at pH 7.0). The possible displacement of EB by the compound sand subsequently the intercalating effect was studied by the stepwise addition of a certain amount of the solution of each compound into the solution of the CT DNA−EB adduct. The solutions were excited at 540 nm and the emission was monitored from 550−700 nm with λ_{max} = 592−594 nm and the effect of the addition of the compounds to the CT−DNA EB solution was recorded. The compounds did not display any fluorescence emission bands at room temperature in solution or in the presence of CT DNA or EB under the same experimental conditions ($\lambda_{excitation}$ = 540 nm); therefore, the observed quenching of the EB−DNA solution may be attributed to the displacement of EB from its EB−DNA adduct.

The Stern−Volmer constants (K_{SV}, in M^{-1}) were calculated according to the linear Stern−Volmer equation (equation S2) [2] and the respective plots Io/I *versus* [compound]:

$$\frac{Io}{I} = 1 + K_q\tau_0[Q] = 1 + K_{SV}[Q] \qquad \text{(equation S2)}$$

where Io and I are the emission intensities of the EB−DNA solution in the absence and the presence of the compound, respectively, τ_o = the average lifetime of the emitting system without the quencher and k_q = the quenching constant. Taking τ_o = 23 ns as the fluorescence lifetime of the EB−DNA adduct [3], the quenching constant of the compound (K_q, in $M^{-1}s^{-1}$) was calculated according to equation [2]:

$$K_{SV} = K_q \times \tau_o \qquad \text{(equation S3)}$$

S1.4 Determination of the thermodynamic parameters

In order to determine the thermodynamics parameters enthalpy change (ΔH), entropy change (ΔS) and ΔG, the DNA-binding constants of the compounds were determined for three different temperatures (295 K, 303 K and 310 K) with equation S1 and the corresponding plots. The enthalpy change (ΔH) and the entropy change (ΔS) were calculated with the van't Hoff equation:

$$\ln(K_b) = -\frac{\Delta H}{RT} + \frac{\Delta S}{R} \qquad \text{(equation S4)}$$

where ΔH and ΔS can be determined from the plot of ln(K_b) *versus* (1/T), where – ΔH/R is the slope of the fitting line and ΔS/R is the intercept (R is the universal gas constant). In addition, ΔG was obtained from the Gibb's-Helmholtz equation:

$$\Delta G = \Delta H - T\cdot\Delta S \qquad \text{(equation S5)}$$

S2 Plasmid DNA cleavage experiments

The reaction mixtures (20 μL) containing supercoiled circular pBR322 plasmid DNA stock solution (Form I, 50 μM/base pair, ~500 ng), compounds, and Tris buffer (25 μM, pH 6.8) in Eppendorf PCR tubes were incubated for 30 min at 37 °C and centrifuged under aerobic conditions at room temperature. Afterwards, in the experiments that irradiation was used, the reaction mixtures contained in the Eppendorf PCR tubes, where irradiated at room temperature with UVB light (312 nm) for 30 min, at a distance of 15 cm, and in the case of UVA light (365 nm) and visible light (400 nm) for 2 h, at a distance of 10 cm.

After addition of the gel−loading buffer [6x Orange DNA Loading Dye 10 mM Tris−HCl (pH 7.6), 0.15% orange G, 0.03% xylene cyanol FF, 60% glycerol, and 60 mM EDTA, by Fermentas], the reaction mixtures were loaded on a 1% agarose gel with EB staining. The electrophoresis tank was attached to a power supply at a constant current (75 V for 30 min). The gel was visualized by the Mupid−ONE LED Illuminator and photographed by a Nikon Digital Camera D3400. Quantification of DNA−cleaving activities was performed by integration of the optical density as a function of the band area using the program "Image J" available at the site http://rsb.info.nih.gov/ij/download.html.

The ss% and ds% damages were calculated according to the equations S6 and S7:

$$ss\% = \frac{Form\ II}{(Form\ I + Form\ II + Form\ III)} \times 100 \qquad \text{(equation S6)}$$

$$ds\% = \frac{Form\ III}{(Form\ I + Form\ II + Form\ III)} \times 100 \qquad \text{(equation S7)}$$

where, as Form II we consider Form II of each series minus Form II of the irradiated control DNA and as Form I, we consider Form I of each series. The amount of supercoiled DNA was multiplied by factor of 1.43 to account for reduced EB intercalation into supercoiled DNA [4].

S3 Albumin−binding studies

S3.1 Interaction with albumins

In order to study the interaction of the compounds with the albumins, the compound was initially dissolved in DMSO (1 mM). Mixing of such solutions with the aqueous buffer solutions of the albumins used in the studies never exceeded 5% DMSO (v/v) in the final solution, which was needed due to low aqueous solubility of most compounds.

With the purpose to investigate if the compound can bind to carrier protein like serum albumins, albumin binding studies were carried out by tryptophan fluorescence quenching experiments using BSA or HSA (3 μM) in buffer (containing 15 mM trisodium citrate and 150 mM NaCl at pH 7.0). The quenching of the emission intensity of tryptophan residues of BSA at 344 nm or HSA at 342 nm was monitored using the compound as quenchers with increasing concentration [2]. The fluorescence emission spectra of the compound were also recorded with $\lambda_{excitation}$ = 295 nm; in case that an additional emission band appeared the albumin−fluorescence emission spectra were corrected by subtracting the spectra of the compound. The influence of the inner−filter effect on the measurements was evaluated by equation [5] :

$$I_{corr} = I_{meas} \times 10^{\frac{\varepsilon(\lambda_{exc})cd}{2}} \times 10^{\frac{\varepsilon(\lambda_{em})cd}{2}} \quad \text{(equation S8)}$$

where I_{corr} = corrected intensity, I_{meas} = the measured intensity, c = the concentration of the quencher, d = the cuvette (1 cm), $\varepsilon_{(\lambda exc)}$ and $\varepsilon_{(\lambda em)}$ = the ε of the quencher at the excitation and the emission wavelength, respectively, as calculated from the UV−vis spectra of the compound [5].

The Stern−Volmer and Scatchard graphs are used to study the interaction of the compound with the albumins. According to the Stern−Volmer quenching equation (equation S2), where Io = initial tryptophan fluorescence intensity of albumin, I = tryptophan fluorescence intensity of BSA after the addition of the quencher, K_q = quenching constant, K_{SV} = Stern−Volmer constant, τ_o = average lifetime of albumin without the quencher, and, taking as fluorescence lifetime (τ_o) of tryptophan in albumin at around 10^{-8} s [2], K_{SV} (in M^{-1}) can be obtained by the slope of the diagram Io/I *versus* [compound] (Stern−Volmer plots), and subsequently the quenching constant (K_q, in $M^{-1}s^{-1}$) may be calculated from equation S3.

From the Scatchard equation:

$$\frac{\Delta I/\mathrm{Io}}{[Q]} = \mathrm{nK} - K\frac{\Delta \mathrm{I}}{\mathrm{Io}} \quad \text{(equation S9)}$$

where n is the number of binding sites per albumin and K is the albumin−binding constant (K, in M^{-1}) is calculated from the slope in plots (ΔI/Io)/[compound] *versus* (ΔI/Io) and n is given by the ratio of y intercept to the slope [6].

S3.2 Competitive albumin−fluorescence studies with warfarin and ibuprofen

The competitive studies with warfarin or ibuprofen (as site−markers) [7] were performed by tryptophan fluorescence quenching experiments using a fixed concentration of the albumin and site markers (3 μM) in buffer (containing 15 mM trisodium citrate and 150 mM NaCl at pH 7.0). The

fluorescence emission spectra were recorded in the presence of increasing amounts of the compounds as quenchers with an excitation wavelength of 295 nm. The Scatchard equation (equation S9) [6] and plots were applied on the corrected albumin−fluorescence emission spectra to determine the albumin−binding constant of the compounds in the presence of warfarin or ibuprofen.

S4 Antioxidant activity assay

The antioxidant activity of the compound was evaluated *via* the ability to scavenge *in vitro* free radicals such as DPPH and ABTS and to reduce H_2O_2. All the experiments were carried out at least in triplicate and the standard deviation of absorbance was less than 10% of the mean.

S4.1 Determination of the reducing activity of the radical DPPH

To an ethanolic solution of DPPH (0.1 mM) an equal volume solution of the compounds (0.1 mM) in ethanol was added. Absolute ethanol was also used as control solution. The absorbance at 517 nm was recorded at room temperature after 30 and 60 min in order to examine the possible existence of a potential time−dependence of the DPPH radical scavenging activity.[8] The DPPH−scavenging activity of the compounds was expressed as the percentage reduction of the absorbance values of the initial DPPH solution (DPPH%). NDGA and BHT were used as reference compounds.

S4.2 Assay of radical cation ABTS−scavenging activity

The ABTS assay was performed to determine the activity of the compounds to scavenge the radical cation ABTS. Initially, a water solution of ABTS was prepared (2 mM). ABTS radical cation ($ABTS^{+\cdot}$) was produced by the reaction of ABTS stock solution with potassium persulfate (0.17 mM) and the mixture was stored in the dark at room temperature for 12−16 h before its use. The ABTS was oxidized incompletely because the stoichiometric reaction ratio of ABTS and potassium persulfate is 1:0.5. The absorbance became maximal and stable only after more than 6 h of reaction although the oxidation of the ABTS started immediately. The radical was stable in this form for more than 2 days when allowed to stand in the dark at room temperature. Afterwards, the $ABTS^{+\cdot}$ solution was diluted in ethanol to an absorbance of 0.70 at 734 nm and 10 μL of diluted compounds or standards (0.1 mM) in DMSO were added. The absorbance was recorded out exactly 1 min after initial mixing [8]. The ABTS-radical scavenging activity of the compounds was expressed as the percentage inhibition of the absorbance of the initial ABTS solution (ABTS%). Trolox was used as an appropriate standard.

S4.3 Reduction of hydrogen peroxide

The ability of the compounds to reduce hydrogen peroxide (H_2O_2) was estimated according to the method described in the literature [9]. The reaction mixture contained 20 μL of each of the tested compounds (0.1 mM) and 5 μL H_2O_2 solution (40 mM) in phosphate buffer (50 mM, pH 7.4). The absorbance was measured at 230 nm after 10 min. The antioxidant activity (reduction of H_2O_2) of the compounds was expressed as the percentage decrease of the initial H_2O_2 solution (H_2O_2%). L−ascorbic acid (or vitamin C) was used as a standard.

S5 References

[1] A. Wolfe, G.H. Shimer, T. Meehan, Polycyclic Aromatic Hydrocarbons Physically Intercalate into Duplex Regions of Denatured DNA, Biochemistry 26 (1987) 6392–6396. https://doi.org/10.1021/bi00394a013.

[2] J.R. Lakowicz, Principles of fluorescence spectroscopy, Springer, 2006. https://doi.org/10.1007/978-0-387-46312-4/COVER.

[3] D.P. Heller, C.L. Greenstock, Fluorescence lifetime analysis of DNA intercalated ethidium bromide and quenching by free dye, Biophys Chem 50 (1994) 305–312. https://doi.org/10.1016/0301-4622(93)E0101-A.

[4] A. Papastergiou, S. Perontsis, P. Gritzapis, A.E. Koumbis, M. Koffa, G. Psomas, K.C. Fylaktakidou, Evaluation of O-alkyl and aryl sulfonyl aromatic and heteroaromatic amidoximes as novel potent DNA photo-cleavers, Photochemical and Photobiological Sciences 15 (2016) 351–360. https://doi.org/10.1039/c5pp00439j.

[5] L. Stella, A.L. Capodilupo, M. Bietti, A reassessment of the association between azulene and [60]fullerene. Possible pitfalls in the determination of binding constants through fluorescence spectroscopy, Chemical Communications (2008) 4744–4746. https://doi.org/10.1039/b808357f.

[6] Y.-Q. Wang, H.-M. Zhang, G.-C. Zhang, W.-H. Tao, S.-H. Tang, Interaction of the flavonoid hesperidin with bovine serum albumin: A fluorescence quenching study, J Lumin 126 (2007) 211–218. https://doi.org/10.1016/J.JLUMIN.2006.06.013.

[7] M. Lazou, A. Tarushi, P. Gritzapis, G. Psomas, Transition metal complexes with a novel guanine-based (E)-2-(2-(pyridin-2-ylmethylene)hydrazinyl)quinazolin-4(3H)-one: Synthesis, characterization, interaction with DNA and albumins and antioxidant activity, J Inorg Biochem 206 (2020) 111019. https://doi.org/10.1016/j.jinorgbio.2020.111019.

[8] C. Kontogiorgis, D. Hadjipavlou-Litina, Biological evaluation of several coumarin derivatives designed as possible anti-inflammatory/antioxidant agents, J Enzyme Inhib Med Chem 18 (2003) 63–69. https://doi.org/10.1080/1475636031000069291.

[9] R.J. Ruch, S. Cheng, J.E. Klaunig, Prevention of cytotoxicity and inhibition of intercellular communication by antioxidant catechins isolated from Chinese green tea, Carcinogenesis 10 (1989) 1003–1008. https://doi.org/10.1093/CARCIN/10.6.1003.

[10] J. de Meulenaer, H. Tompa, The absorption correction in crystal structure analysis, Acta Crystallogr 19 (1965) 1014–1018. https://doi.org/10.1107/S0365110X65004802.

TABLES

Table S1. Experimental crystallographic details for complexes **1** and **2**.

	**Complex 1**	**Complex 2**
Crystal data		
Chemical formula	$C_{33}H_{21}FeO_6$	$C_{49}H_{46}ClFe_2KO_{19}$
M_r	569.37	1125.14
Crystal system	Monoclinic	Triclinic
Space group	*C*2/*c*	*P*-1
Temperature (K)	295	295
a (Å)	19.439 (3)	12.9316 (9)
b (Å)	24.178 (3)	13.1757 (10)
c (Å)	13.7640 (19)	15.9281 (11)
α (°)	90	106.397 (2)
β (°)	127.213 (3)	92.980 (2)
γ (°)	90	94.284 (2)
V ($Å^3$)	5151.8 (12)	2588.6 (3)
Z	8	2
Radiation type	Mo *K*α	Mo *K*α
μ (mm^{-1})	0.63	0.77
Crystal size (mm)	0.18 × 0.11 × 0.04	0.27 × 0.22 × 0.09
Data collection		
Diffractometer	Bruker Kappa Apex2	
Absorption correction	Numerical<br>Analytical Absorption [10]	
T_{min}, T_{max}	0.93, 0.97	0.84, 0.93
No. of measured reflections	27806	41392
No. of independent reflections	4707	9773
No. of observed [$I > 2.0\sigma(I)$] reflections	3152	8106
R_{int}	0.038	0.023
$(\sin \theta/\lambda)_{max}$ ($Å^{-1}$)	0.606	0.612
Refinement		
$R[F^2 > 2\sigma(F^2)]$, $wR(F^2)$, S	0.056, 0.097, 1.00	0.046, 0.075, 1.00
No. of reflections	3152	8106
No. of parameters	361	656
No. of restraints		2
H-atom treatment	H-atom parameters constrained	
$\Delta\rho_{max}$, $\Delta\rho_{min}$ (e $Å^{-3}$)	0.67, −0.34	0.77, −0.42

Table S2. Selected bonds lengths (in Å) and angles (in °) for complex **1**.

**Bond**	**Length (Å)**	**Bond**	**Length (Å)**
Fe1—O1	1.976 (4)	Fe1—O4	1.965 (3)
Fe1—O2	2.024 (3)	Fe1—O5	2.042 (3)
Fe1—O3	1.994 (4)	Fe1—O6	1.967 (3)
**Bond**	**Angle (°)**	**Bond**	**Angle (°)**
O1—Fe1—O2	86.58 (14)	O2—Fe1—O3	88.95 (14)
O1—Fe1—O3	175.24 (13)	O2—Fe1—O4	96.51 (13)
O1—Fe1—O4	92.91 (15)	O2—Fe1—O5	83.41 (13)
O1—Fe1—O5	94.80 (14)	O2—Fe1—O6	169.30 (14)
O1—Fe1—O6	89.35 (15)	O4—Fe1—O5	172.27 (16)
O3—Fe1—O4	85.98 (15)	O4—Fe1—O6	93.57 (13)
O3—Fe1—O5	86.29 (15)	O5—Fe1—O6	87.08 (13)
O3—Fe1—O6	95.33 (15)		

Table S3. Selected bonds lengths (in Å) and angles (in °) for complex **2**.

**Bond**	**Length (Å)**	**Bond**	**Length (Å)**
Fe1—O1	2.0673 (18)	Fe2—O10	2.049 (2)
Fe1—O2	1.9344 (16)	Fe2—O11	1.9346 (19)
Fe1—O4	2.0656 (18)	Fe2—O13	2.070 (2)
Fe1—O5	1.9357 (16)	Fe2—O14	1.933 (2)
Fe1—O7	2.0755 (17)	Fe2—O16	2.080 (2)
Fe1—O8	1.9384 (17)	Fe2—O17	1.942 (2)
K1…O2	2.9761 (18)	K1…O11	2.8776 (19)
K1…O3	3.101 (2)	K1…O12	3.097 (2)
K1…O5	2.9155 (17)	K1…O14	2.8616 (19)
K1…O6	3.078 (2)	K1…O15	3.136 (2)
K1…O8	2.8843 (17)	K1…O17	2.998 (2)
K1…O9	3.054 (2)	K1…O18	3.227 (2)
**Bond**	**Angle (°)**	**Bond**	**Angle (°)**
O1—Fe1—O2	86.51 (7)	O10—Fe2—O11	86.55 (8)
O1—Fe1—O4	84.65 (7)	O10—Fe2—O13	84.38 (8)
O2—Fe1—O4	100.31 (8)	O11—Fe2—O13	169.44 (9)
O1—Fe1—O5	170.14 (7)	O10—Fe2—O14	100.95 (9)
O2—Fe1—O5	91.69 (7)	O11—Fe2—O14	90.23 (9)
O4—Fe1—O5	86.14 (7)	O13—Fe2—O14	86.20 (8)
O1—Fe1—O7	83.41 (8)	O10—Fe2—O16	82.35 (10)
O2—Fe1—O7	169.06 (8)	O11—Fe2—O16	100.29 (9)
O4—Fe1—O7	82.98 (7)	O13—Fe2—O16	83.87 (9)
O5—Fe1—O7	98.97 (8)	O14—Fe2—O16	169.18 (9)
O1—Fe1—O8	99.13 (7)	O10—Fe2—O17	166.36 (9)
O2—Fe1—O8	90.95 (7)	O11—Fe2—O17	90.06 (8)
O4—Fe1—O8	168.35 (7)	O13—Fe2—O17	100.00 (8)
O5—Fe1—O8	90.59 (7)	O14—Fe2—O17	92.26 (8)
O7—Fe1—O8	86.49 (7)	O16—Fe2—O17	85.27 (9)
O2...K1...O5	56.22 (5)	O8...K1...O17	123.19 (6)
O2...K1...O6	101.10 (5)	O9...K1...O17	80.49 (6)
O5...K1...O6	51.09 (5)	O2...K1...O3	50.36 (5)
O2...K1...O8	56.19 (5)	O5...K1...O3	99.99 (6)
O5...K1...O8	56.68 (5)	O6...K1...O3	119.98 (6)
O6...K1...O8	100.55 (5)	O8...K1...O3	99.64 (5)
O2...K1...O9	101.16 (5)	O9...K1...O3	117.86 (6)
O5...K1...O9	102.21 (5)	O2...K1...O18	127.81 (5)
O6...K1...O9	119.15 (6)	O5...K1...O18	77.63 (5)
O8...K1...O9	52.09 (5)	O6...K1...O18	58.04 (5)
O2...K1...O11	128.73 (6)	O8...K1...O18	79.61 (5)

O5...K1...O11	174.99 (6)	O9...K1...O18	63.53 (6)
O6...K1...O11	124.33 (5)	O11...K1...O12	51.69 (5)
O8...K1...O11	125.68 (6)	O11...K1...O14	57.04 (6)
O9...K1...O11	78.11 (6)	O12...K1...O14	102.17 (6)
O2...K1...O12	84.40 (5)	O11...K1...O15	100.32 (6)
O5...K1...O12	132.67 (5)	O12...K1...O15	118.03 (6)
O6...K1...O12	174.13 (6)	O14...K1...O15	51.17 (5)
O8...K1...O12	80.72 (5)	O11...K1...O17	55.61 (5)
O9...K1...O12	57.16 (6)	O12...K1...O17	99.95 (6)
O2...K1...O14	123.46 (6)	O14...K1...O17	56.89 (6)
O5...K1...O14	120.51 (6)	O15...K1...O17	102.74 (6)
O6...K1...O14	76.61 (6)	O11...K1...O3	84.14 (6)
O8...K1...O14	177.10 (6)	O12...K1...O3	65.15 (6)
O9...K1...O14	129.79 (6)	O14...K1...O3	81.41 (6)
O2...K1...O15	75.93 (5)	O15...K1...O3	57.02 (6)
O5...K1...O15	79.79 (5)	O17...K1...O3	132.54 (6)
O6...K1...O15	65.80 (6)	O11...K1...O18	98.21 (6)
O8...K1...O15	127.21 (5)	O12...K1...O18	116.90 (6)
O9...K1...O15	174.87 (6)	O14...K1...O18	99.24 (6)
O2...K1...O17	175.52 (5)	O15...K1...O18	121.60 (6)
O5...K1...O17	119.43 (5)	O17...K1...O18	49.16 (6)
O6...K1...O17	74.52 (5)	O3...K1...O18	177.53 (6)

Table S4. Thermodynamic parameters of the compounds for the interaction with CT DNA at different temperatures (295 K, 303 K and 310 K).

Compound	T (K)	K_b (M^{-1})	ΔG (kJ mol^{-1})	ΔH (KJ mol^{-1})	ΔS (J mol^{-1} K^{-1})
Complex **1**	298	1.62×10^{7}	-4.27	+1.47	+19.26
	303	1.86×10^{7}	-4.47		
	310	2.03×10^{7}	-4.50		
Complex **2**	298	1.65×10^{6}	-3.68	+3.94	+25.56
	303	1.98×10^{6}	-3.79		
	310	2.96×10^{6}	-3.98		
Complex **3**	298	9.12×10^{5}	-3.53	+2.30	+19.56
	303	1.12×10^{6}	-3.64		
	310	1.30×10^{6}	-3.76		
Complex **4**	298	8.89×10^{4}	-2.93	+4.85	+26.14
	303	1.38×10^{5}	-3.09		
	310	1.87×10^{5}	-3.25		
Complex **5**	298	6.64×10^{5}	-3.45	+1.05	+15.09
	303	6.80×10^{5}	-3.51		
	310	7.75×10^{5}	-3.63		
Complex **6**	298	6.17×10^{5}	-3.43	+2.89	+21.20
	303	7.09×10^{5}	-3.52		
	310	9.51×10^{5}	-3.68		
Complex **7**	298	9.06×10^{5}	-3.53	+2.15	+19.09
	303	1.12×10^{6}	-3.64		
	310	1.26×10^{6}	-3.76		
Complex **8**	298	1.17×10^{6}	-3.59	+1.53	+17.17
	303	1.22×10^{6}	-3.66		
	310	1.47×10^{6}	-3.80		

FIGURES

Figure S1. IR spectra (ATR) of the complexes.

Complex **1**

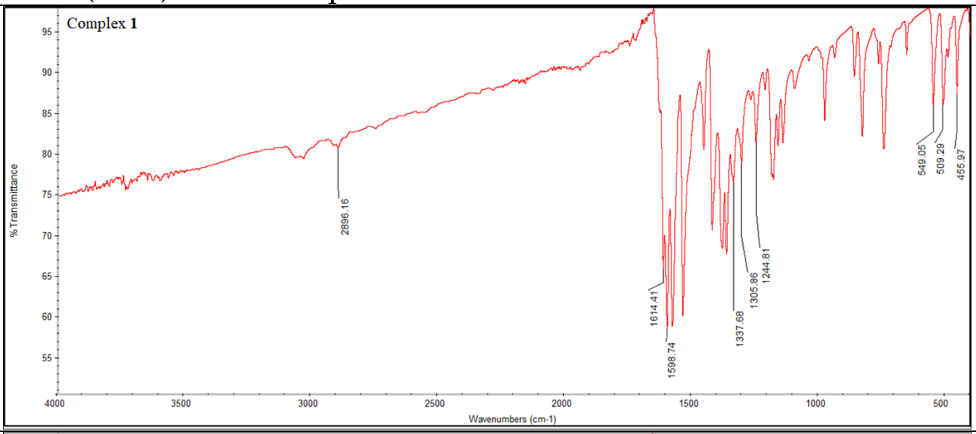

Complex **2**

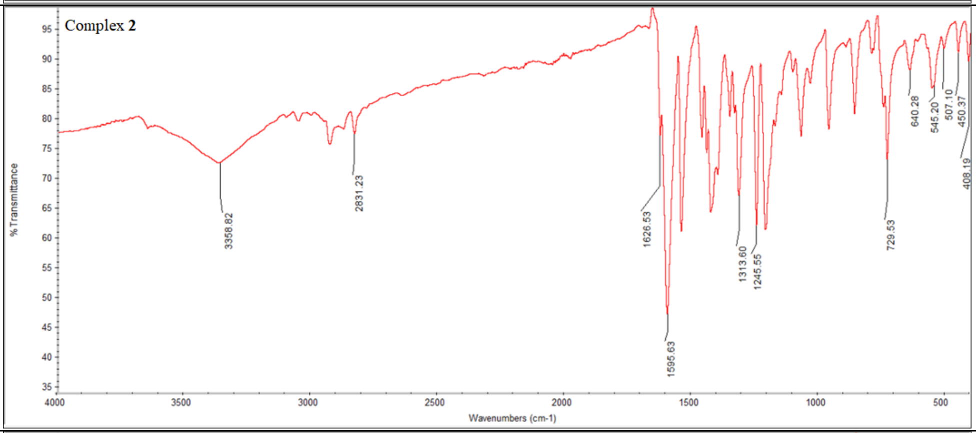

Complex **3**

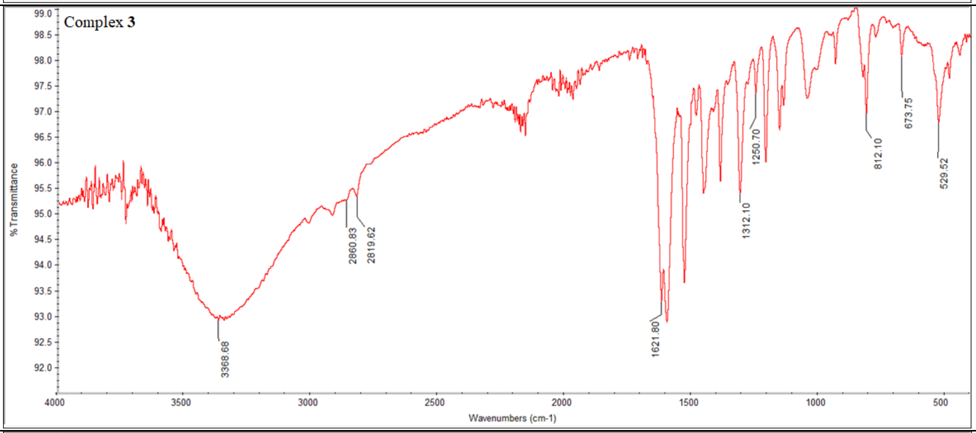

Complex **4**

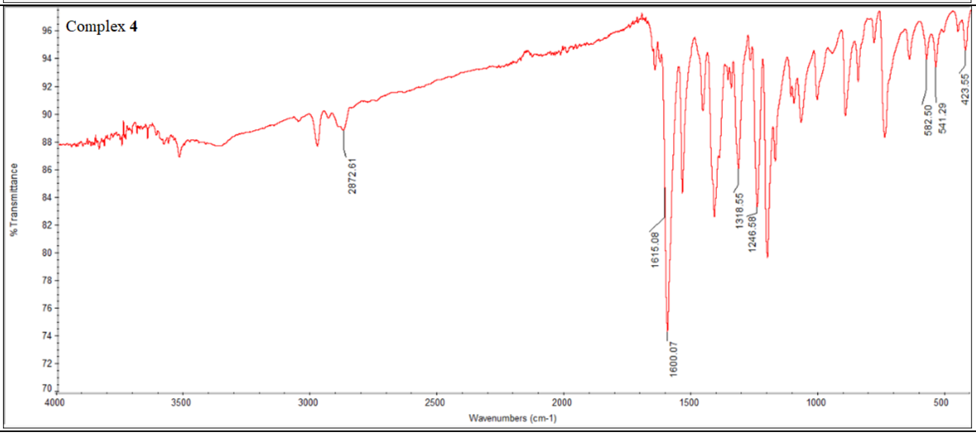

Complex **5**

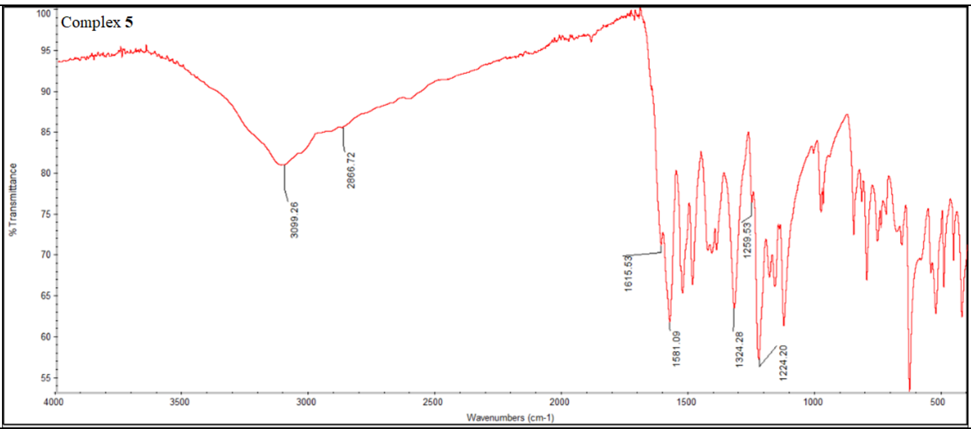

Complex **6**

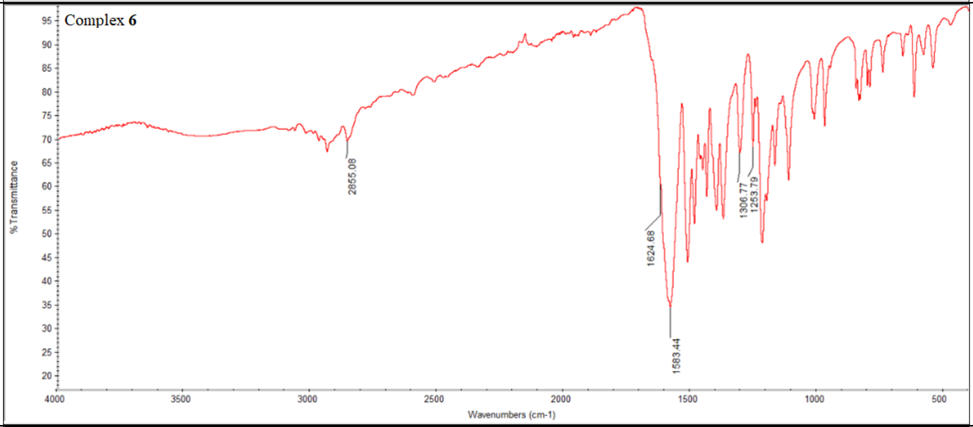

Complex **7**

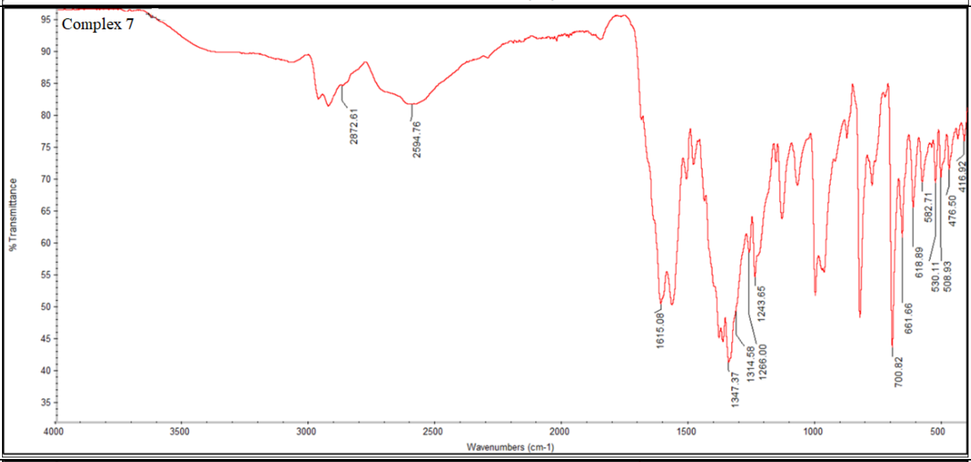

Complex **8**

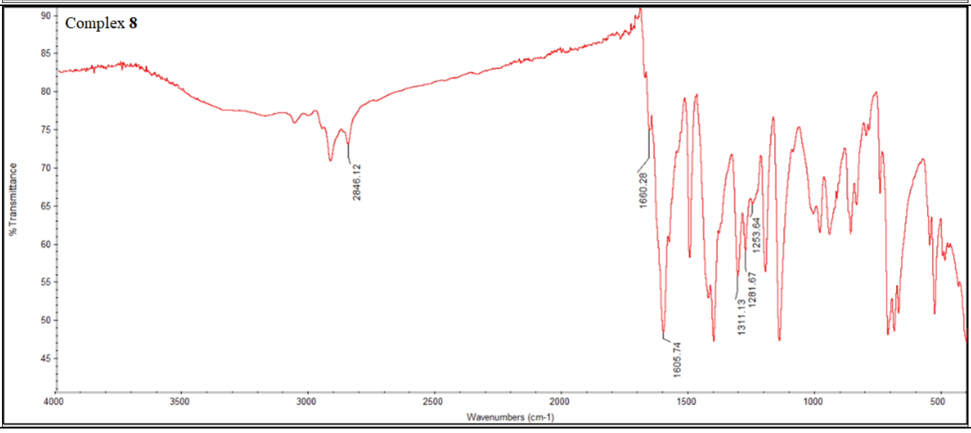

Figure S2: Proposed structures for complexes **3-8.**

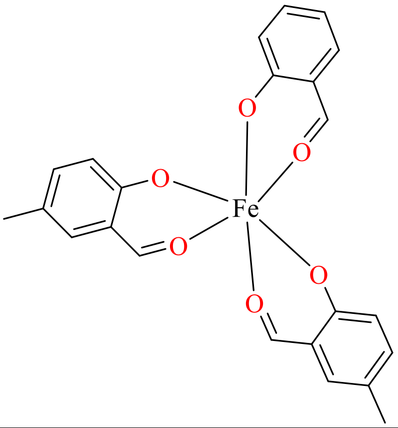	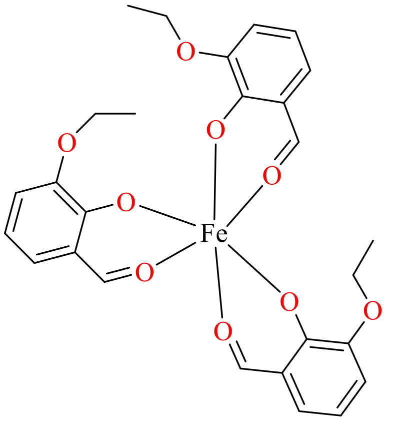
Complex **3**	Complex **4**
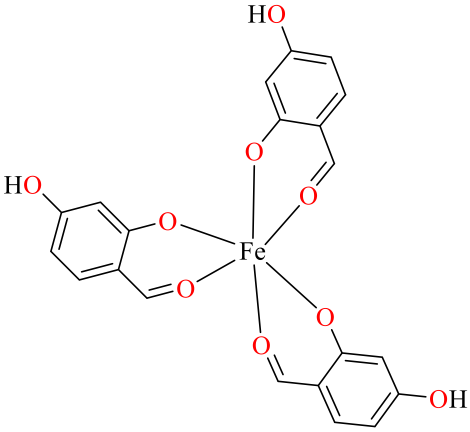	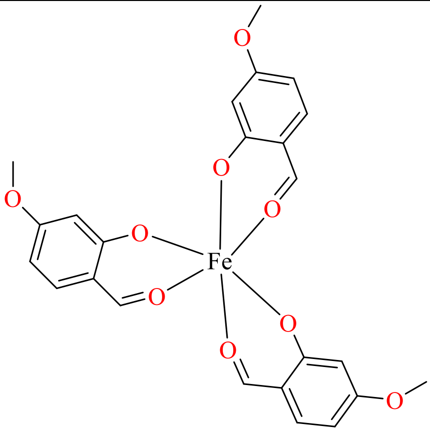
Complex **5**	Complex **6**
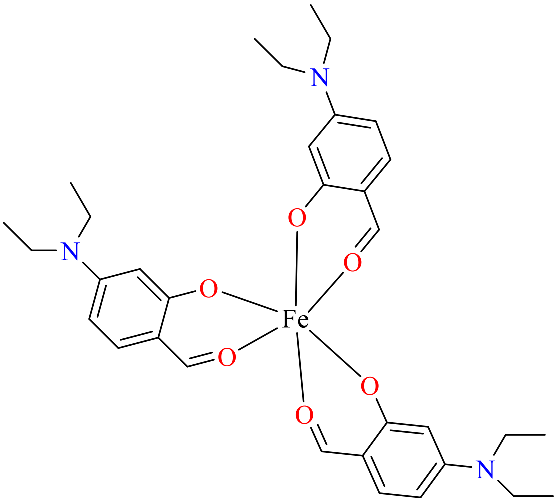	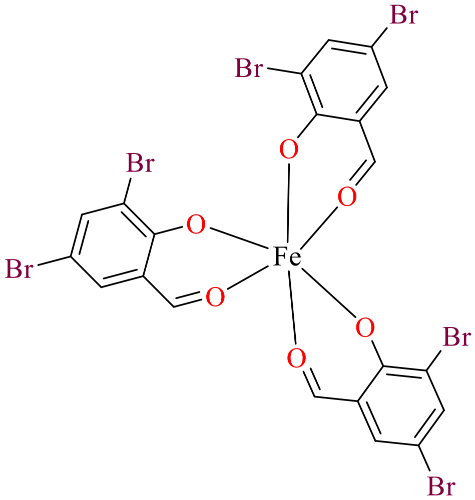
Complex **7**	Complex **8**

Figure S3. UV−vis spectra of a DMSO solution of complexes **1-8**, in the presence of increasing amounts of CT DNA.

The concentrations of the solution of the compounds are given in parentheses. The arrows show the changes upon increasing amounts of CT DNA.

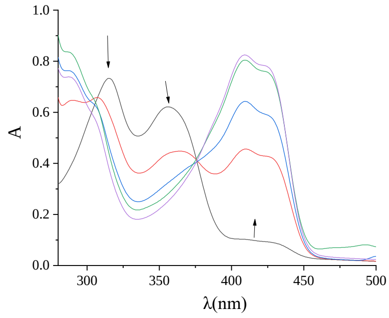

Complex **1** (100 µM)

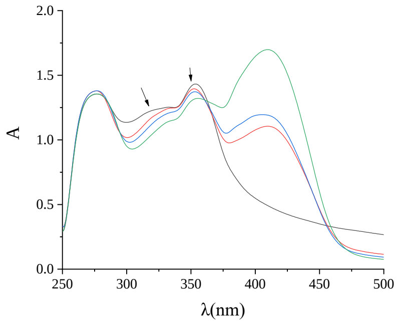

Complex **2** (100 µM)

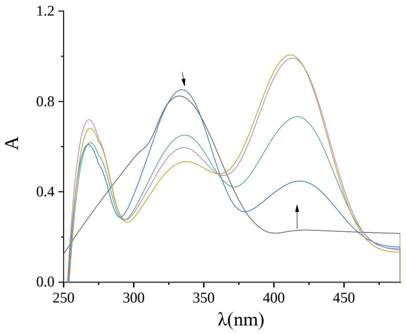

Complex **3** (100 µM)

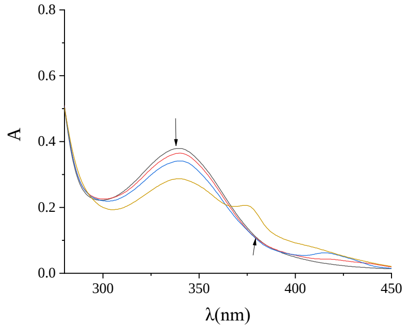

Complex **4** (40 µM)

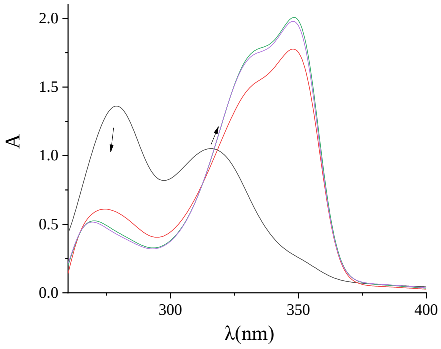

Complex **5** (50 µM)

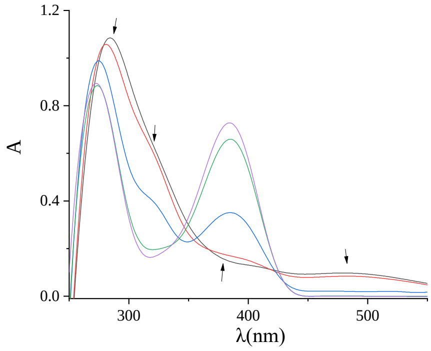

Complex **6** (75 µM)

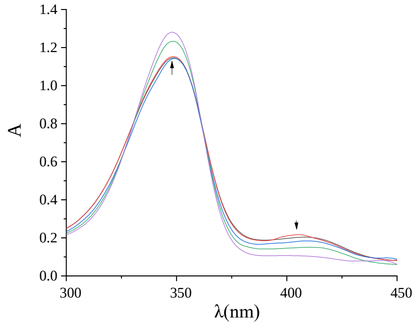

Complex **7** (40 µM)

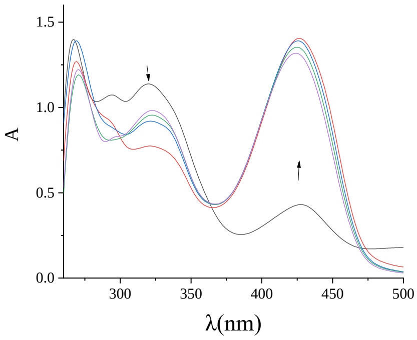

Complex **8** (100 µM)

Figure S4. Plots of $\frac{[\text{DNA}]}{(\varepsilon_A-\varepsilon_f)}$ *versus* [DNA] for complexes **1-8**.

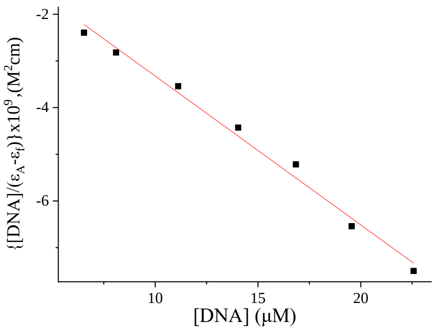

Complex **1**

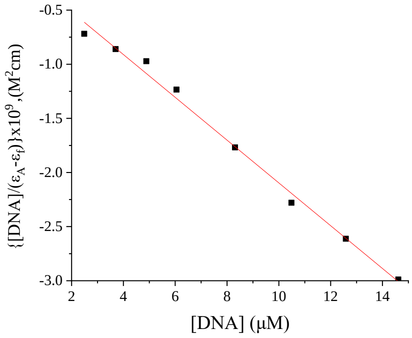

Complex **2**

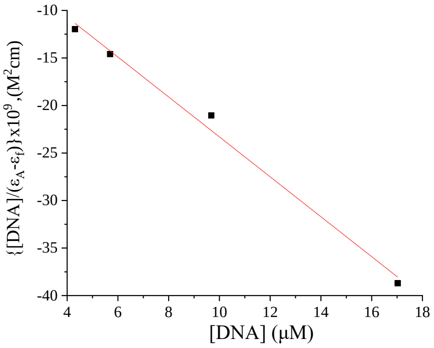

Complex **3**

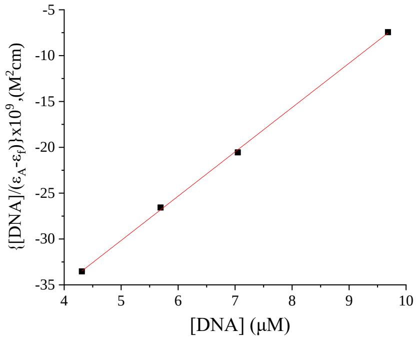

Complex **4**

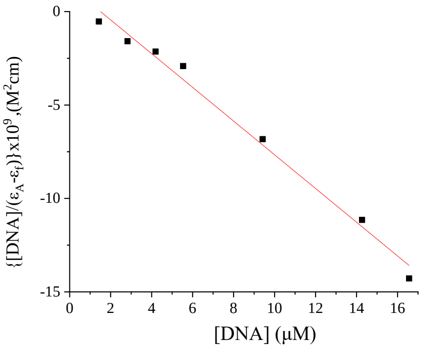

Complex **5**

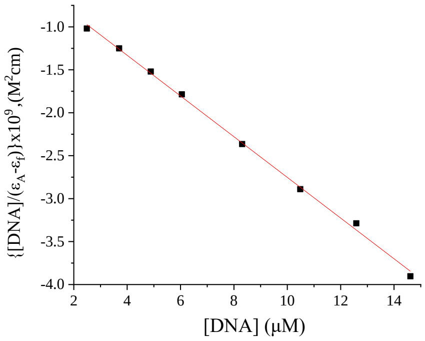

Complex **6**

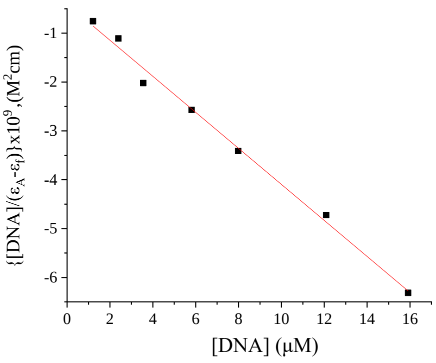

Complex **7**

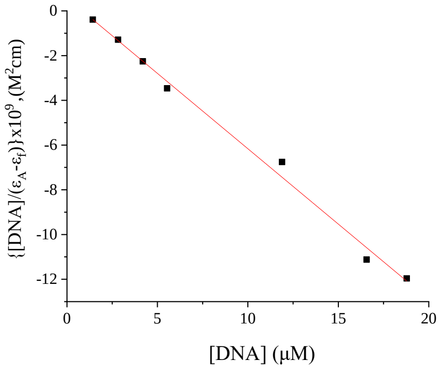

Complex **8**

Figure S5. Fluorescence emission spectra for EB-DNA in buffer solution in the absence and presence of increasing amounts of the compounds.
Conditions: $\lambda_{excitation}$ = 540 nm. [EB] = 40 μM. [DNA] = 45 μM. Buffer solution: 150 mM NaCl and 15 mM trisodium citrate at pH 7.0. The arrow shows the changes of intensity upon increasing amounts of the compound.

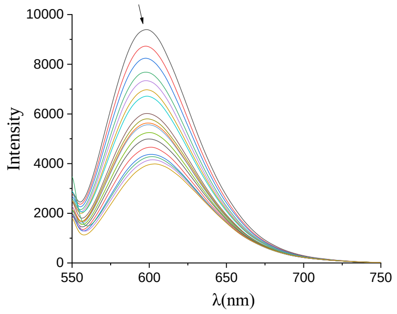

Complex **1**

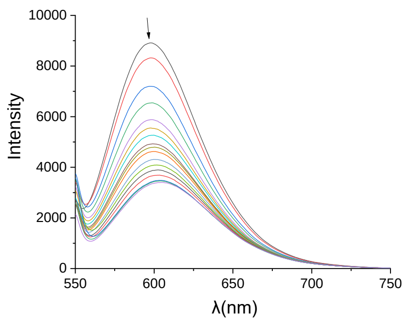

Complex **2**

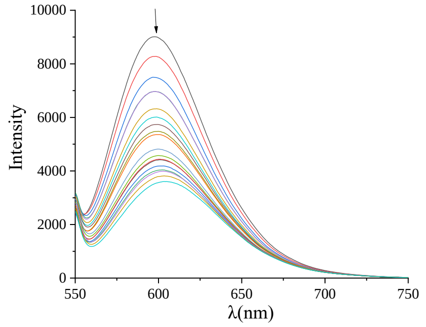

Complex **3**

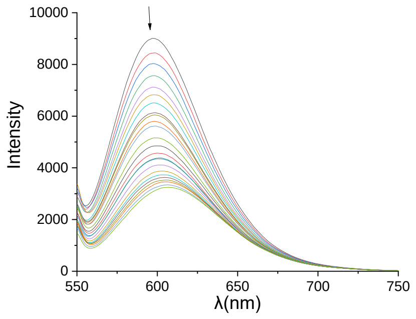

Complex **4**

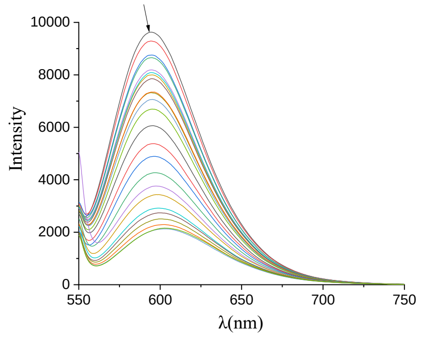

Complex **5**

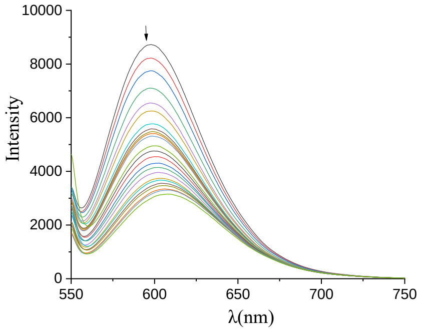

Complex **6**

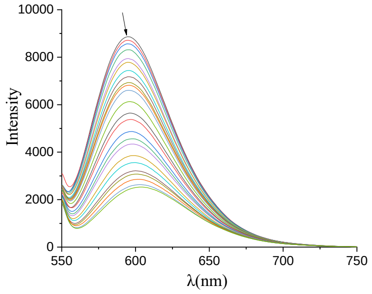

Complex **7**

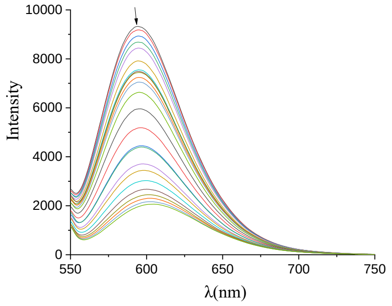

Complex **8**

Figure S6. Stern−Volmer plots of the EB−DNA quenching experiments upon addition of the compounds.

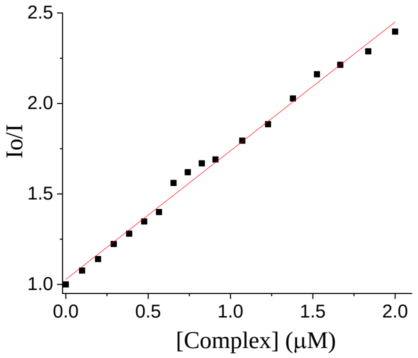	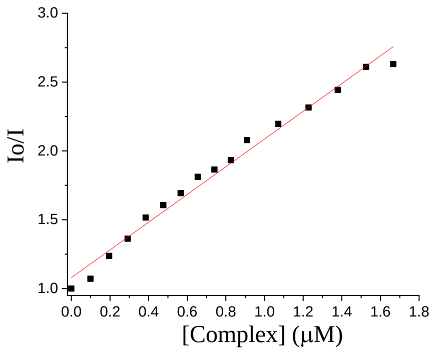
Complex **1**	Complex **2**
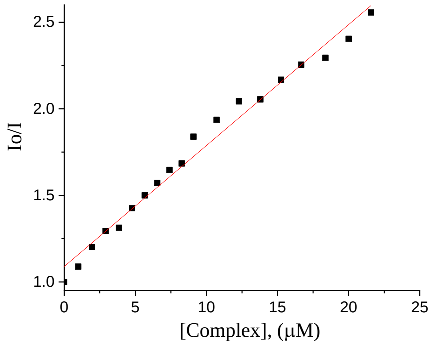	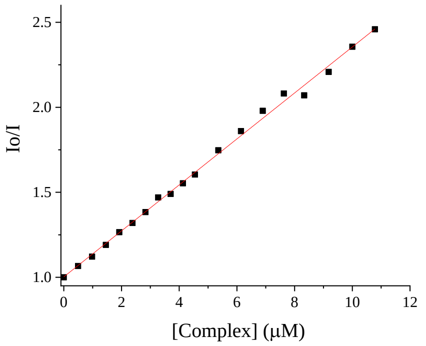
Complex **3**	Complex **4**
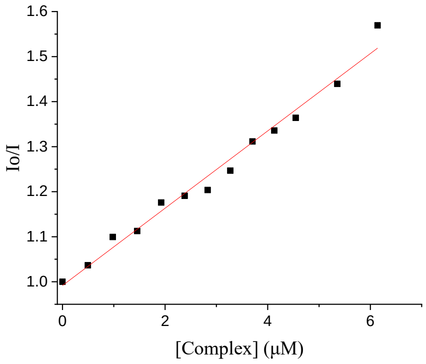	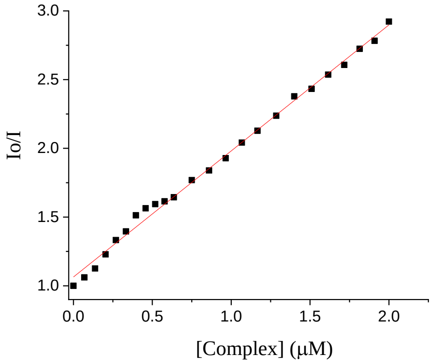
Complex **5**	Complex **6**
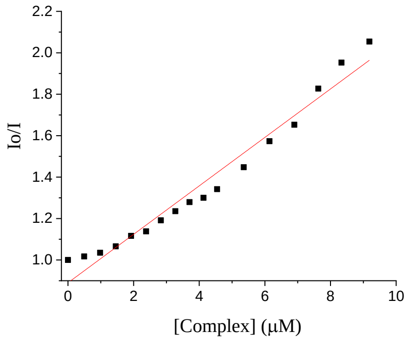	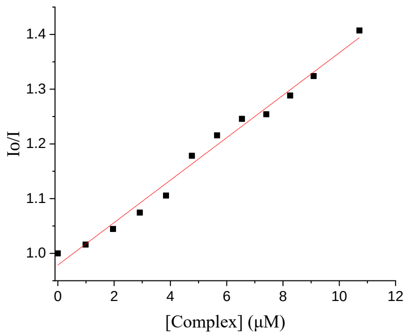
Complex **7**	Complex **8**

Figure S7. van't Hoff plots for the interaction of CT DNA with the compounds.

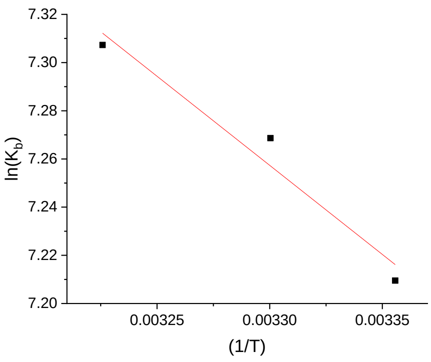

Complex **1**

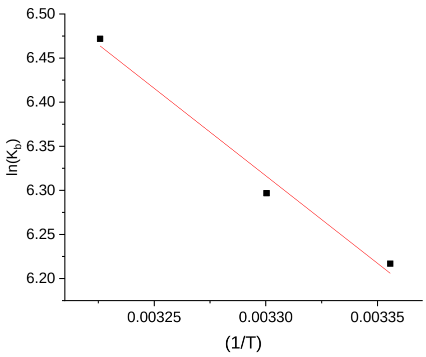

Complex **2**

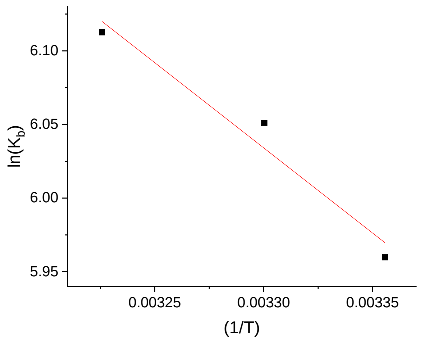

Complex **3**

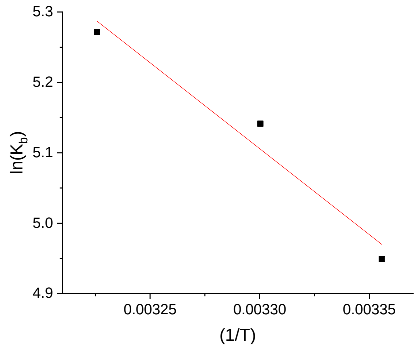

Complex **4**

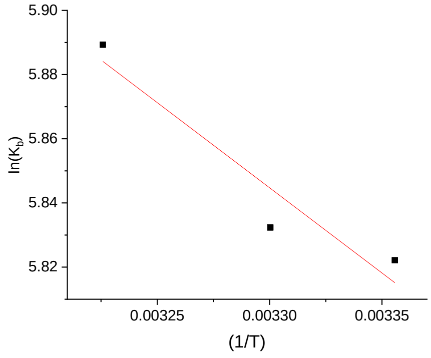

Complex **5**

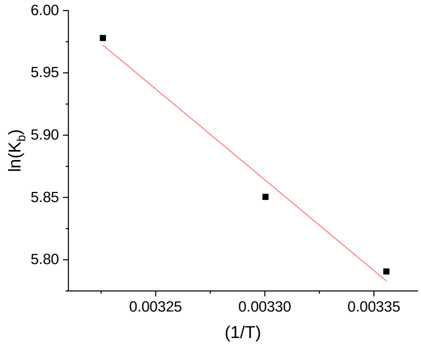

Complex **6**

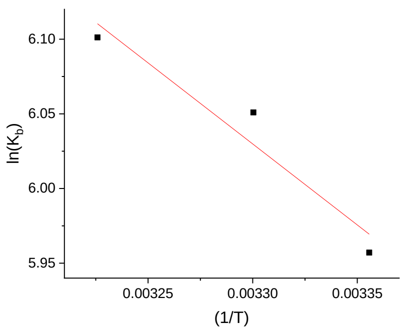

Complex **7**

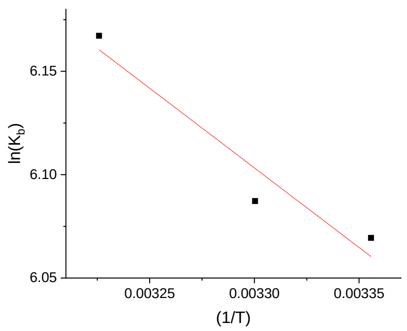

Complex **8**

Figure S8: Agarose gel electrophoretic pattern of EB-stained plasmid DNA (pBR322 plasmid DNA) after incubation with the compounds, in dark.
Conditions: [pDNA] = 50 μM/base pair. [compound] = 500 μM. Power supply: 65 V for 1 h.
Top: gel electrophoresis pictures: lane 1: pDNA; lane 2: complex **1** + pDNA; lane 3: complex **2** + pDNA; lane 4: complex **3** + pDNA; lane 5: complex **4** + pDNA; lane 6: complex **5** + pDNA; lane 7: complex **6** + pDNA; lane 8: complex **7** + pDNA; lane 9: complex **8** + pDNA
Bottom: calculation of the % conversion to ss and ds damage. DNA forms: form I = supercoiled pDNA, form II = relaxed pDNA, and form III = linear pDNA.

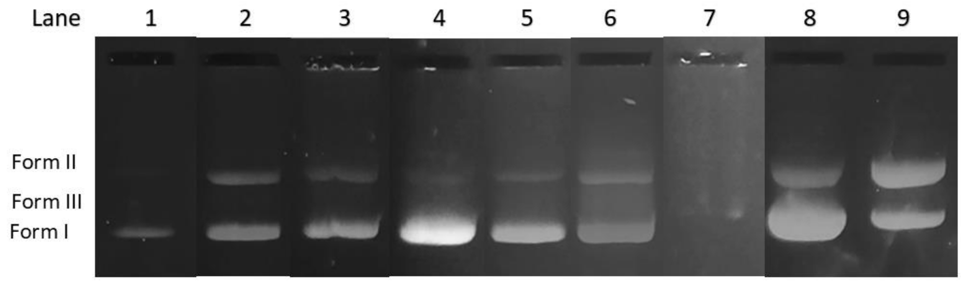

	Lane 1	Lane 2	Lane 3	Lane 4	Lane 5	Lane 6	Lane 7	Lane 8	Lane 9
Form II		18	21	14	23	29	10	24	43
Form III		0	0	0	0	0	0	0	0

Figure S9: Agarose gel electrophoretic pattern of EB-stained plasmid DNA (pBR322 plasmid DNA) with the compounds, after irradiation at 312 nm (UV-B) for 30 min.
Conditions: [pDNA] = 50 μM/base pair; [compound] = 500 μM. Power supply: 65 V for 1 h.
Top: gel electrophoresis pictures: lane 1: pDNA + irradiation; lane 2: complex **1** + pDNA+ irradiation; lane 3: complex **2** + pDNA+ irradiation; lane 4: complex **3** + pDNA+ irradiation; lane 4: complex **4** + pDNA+ irradiation; lane 6: complex **5** + pDNA+ irradiation; lane 7: complex **6** + pDNA+ irradiation; lane 8: complex **7** + pDNA+ irradiation; lane 9: complex **8** + pDNA+ irradiation.
Bottom: calculation of the % conversion to ss and ds damage. DNA forms: form I = supercoiled pDNA, form II = relaxed pDNA, and form III = linear pDNA. *: it was not possible to calculate.

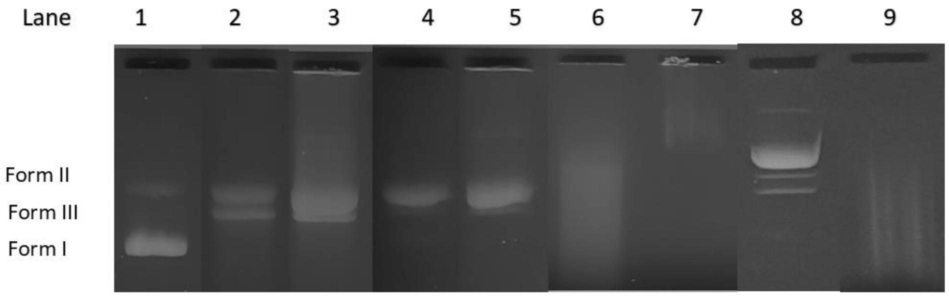

Form II		50	60	78	65	*	*	65	*
Form III		48	35	20	32	*	*	30	*

Figure S10: Agarose gel electrophoretic pattern of EB-stained plasmid DNA (pBR322 plasmid DNA) with the compounds, after irradiation at 365 nm (UV-A) for 30 min.
Conditions: [pDNA] = 50 μM/base pair; [compound] = 500 μM. Power supply: 65 V for 1 h.
Top: gel electrophoresis pictures: lane 1: pDNA + irradiation; lane 2: complex **1** + pDNA+ irradiation; lane 3: complex **2** + pDNA+ irradiation; lane 4: complex **3** + pDNA+ irradiation; lane 5: complex **4** + pDNA+ irradiation; lane 6: complex **5** + pDNA+ irradiation; lane 7: complex **6** + pDNA+ irradiation; lane 8: complex **7** + pDNA+ irradiation; lane 9: complex **8** + pDNA+ irradiation.
Bottom: calculation of the % conversion to ss and ds damage. DNA forms: form I = supercoiled pDNA, form II = relaxed pDNA, and form III = linear pDNA. *: it was not possible to calculate.

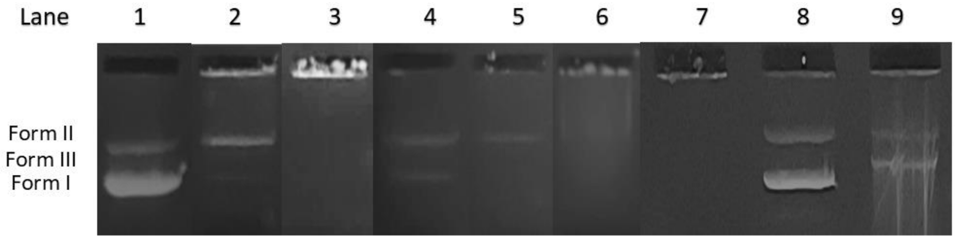

	1	2	3	4	5	6	7	8	9
Form II		57	*	70	85	*	*	60	35
Form III		0	*	0	0	*	*	0	0

Figure S11: Agarose gel electrophoretic pattern of EB-stained plasmid DNA (pBR322 plasmid DNA) with the compounds, after irradiation under visible light for 2 h.
Conditions: [pDNA] = 50 μM/base pair; [compound] = 500 μM. Power supply: 65 V for 1 h.
Top: gel electrophoresis pictures: lane 1: pDNA + irradiation; lane 2: complex **1** + pDNA+ irradiation; lane 3: complex **2** + pDNA+ irradiation; lane 4: complex **3** + pDNA+ irradiation; lane 5: complex **4** + pDNA+ irradiation; lane 6: complex **5** + pDNA+ irradiation; lane 9: complex **6** + pDNA+ irradiation; lane 8: complex **7** + pDNA+ irradiation; lane 9: complex **8** + pDNA+ irradiation.
Bottom: calculation of the % conversion to ss and ds damage. DNA forms: form I = supercoiled pDNA, form II = relaxed pDNA, and form III = linear pDNA. *: it was not possible to calculate.

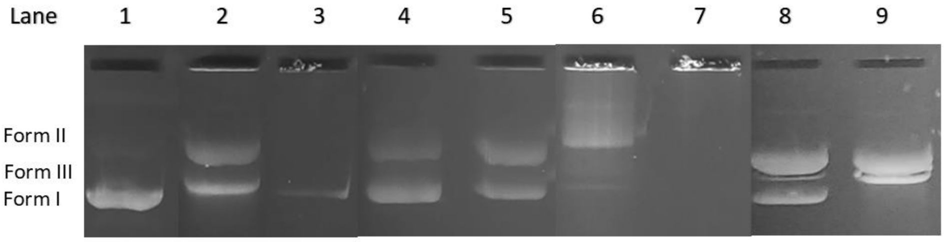

	1	2	3	4	5	6	7	8	9
Form II		45	*	25	51	62	*	29	72
Form III		0	*	0	0	5	*	8	15

Figure S12. Fluorescence emission spectra of BSA in buffer solution in the presence of increasing amounts of the compounds.

Conditions: $\lambda_{excitation}$ = 295 nm. [BSA] = 3 μM. Buffer solution: 150 mM NaCl and 15 mM trisodium citrate at pH 7.0. The arrow shows the changes of intensity upon increasing amounts of the compound.

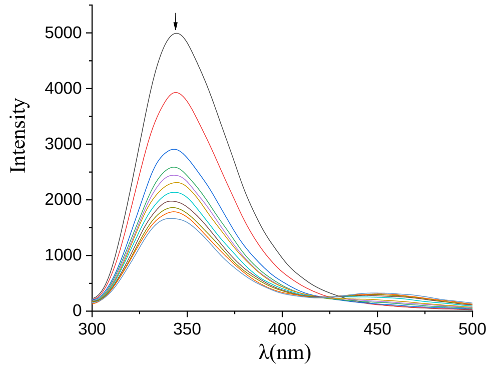

Complex **1**

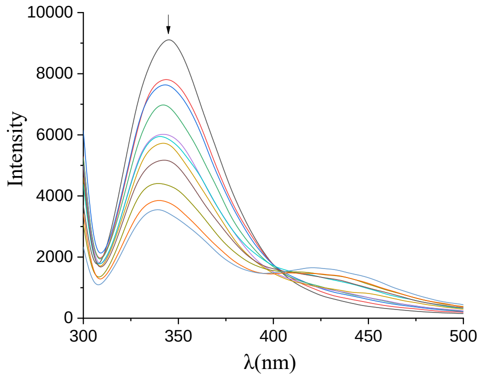

Complex **2**

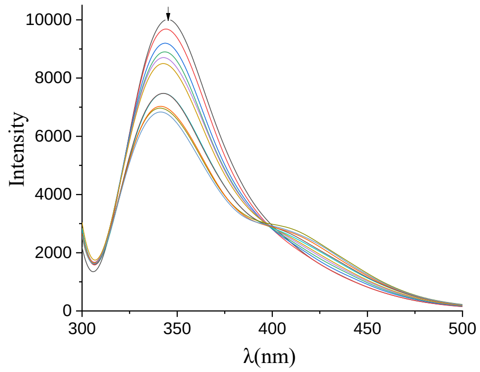

Complex **3**

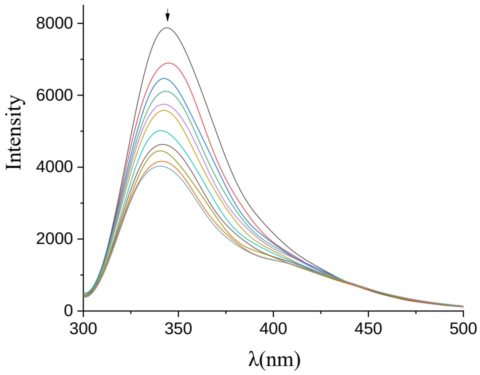

Complex **4**

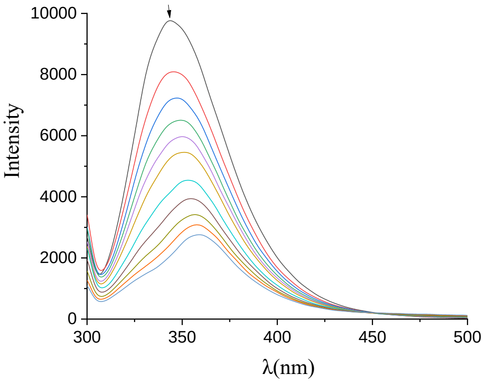

Complex **5**

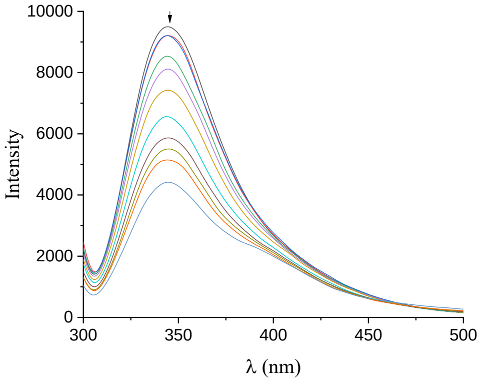

Complex **6**

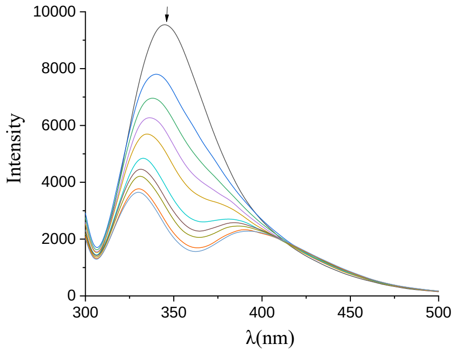

Complex **7**

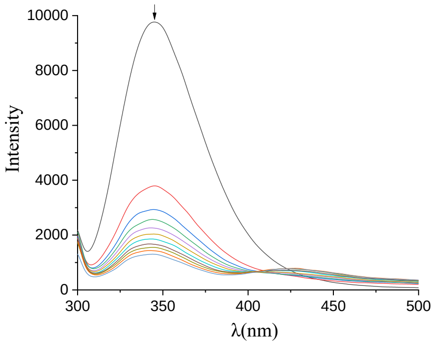

Complex **8**

Figure S13. Fluorescence emission spectra of HSA in buffer solution in the presence of increasing amounts of the compounds.

Conditions: $\lambda_{excitation}$= 295 nm. [HSA] = 3 μM. Buffer solution: 150 mM NaCl and 15 mM trisodium citrate at pH 7.0. The arrow shows the changes of intensity upon increasing amounts of the compound.

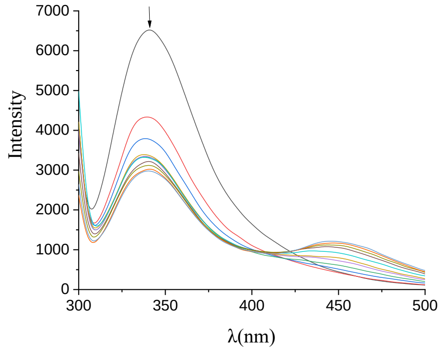

Complex **1**

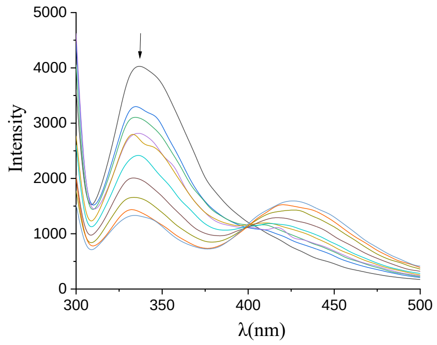

Complex **2**

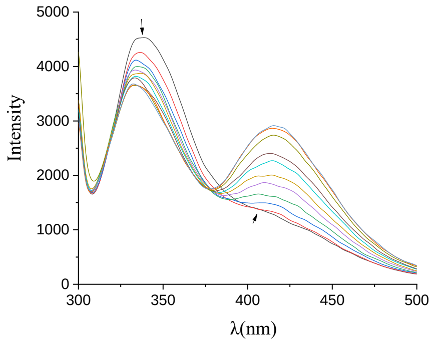

Complex **3**

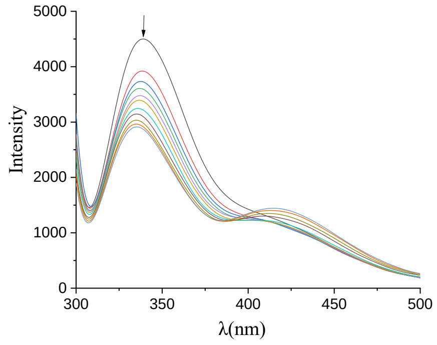

Complex **4**

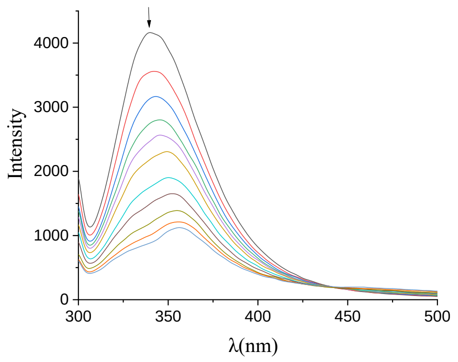

Complex **5**

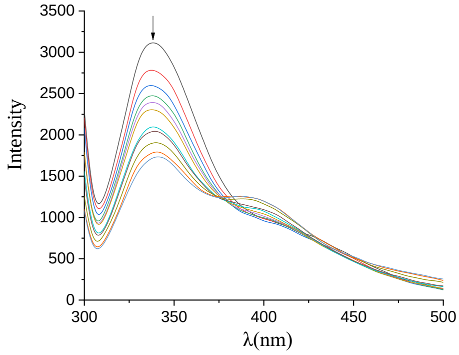

Complex **6**

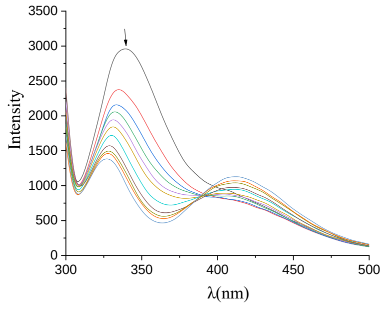

Complex **7**

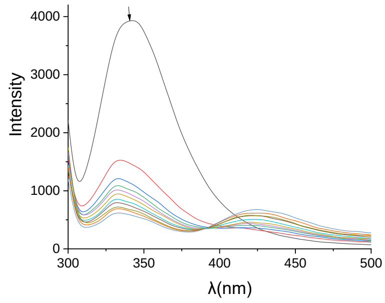

Complex **8**

Figure S14. Stern–Volmer plots of the BSA-quenching experiments upon addition of the compounds.

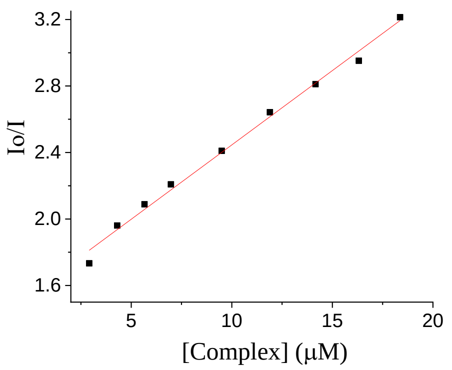

Complex **1**

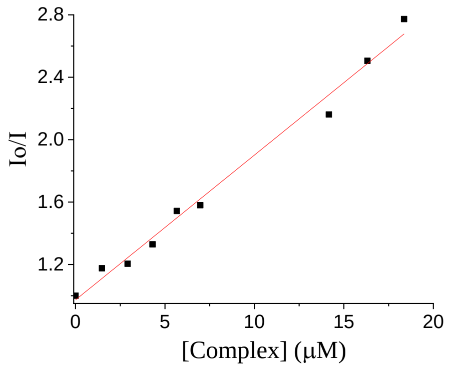

Complex **2**

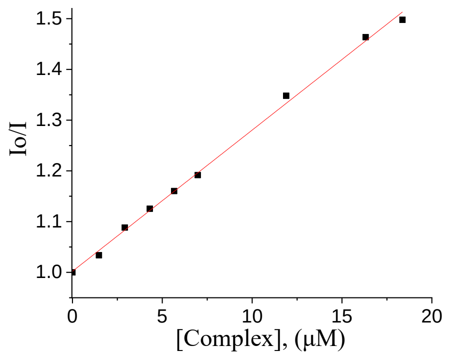

Complex **3**

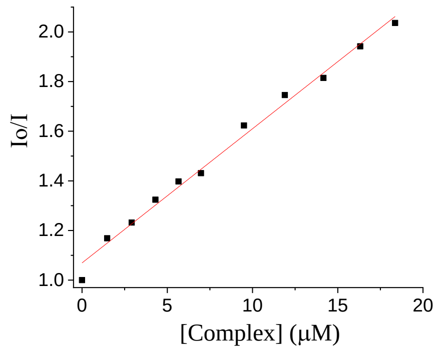

Complex **4**

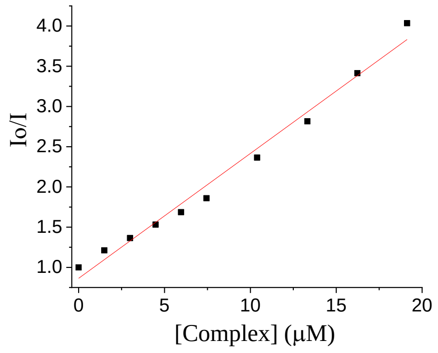

Complex **5**

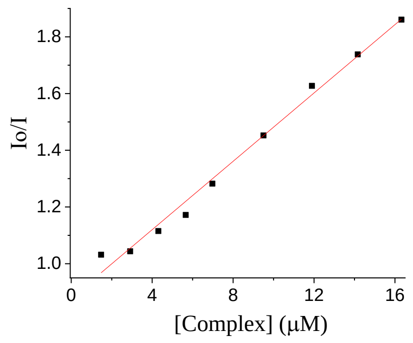

Complex **6**

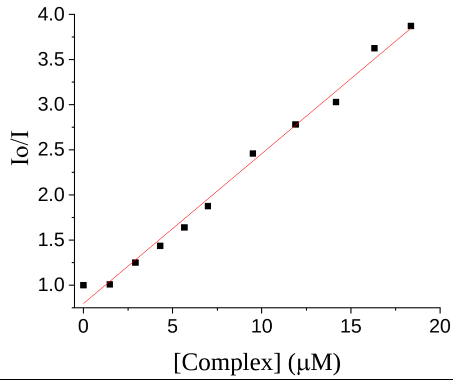

Complex **7**

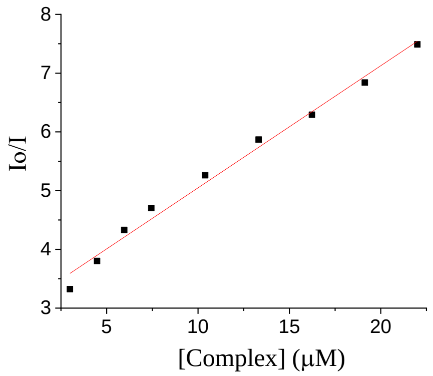

Complex **8**

Figure S15. Stern−Volmer plots of the HSA-quenching experiments upon addition of the compounds.

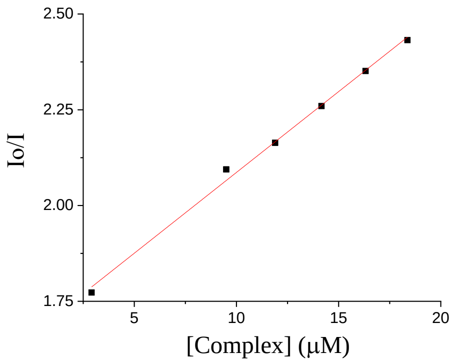

Complex **1**

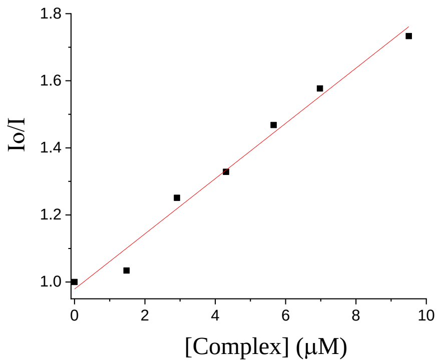

Complex **2**

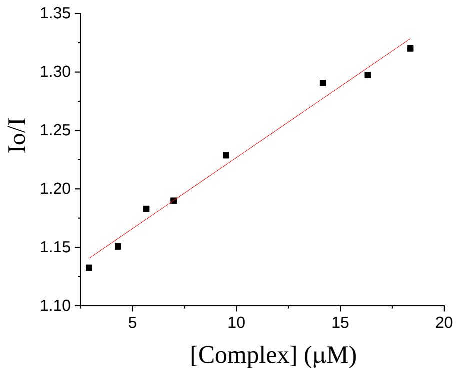

Complex **3**

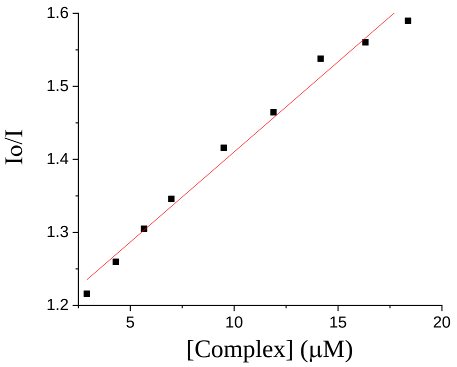

Complex **4**

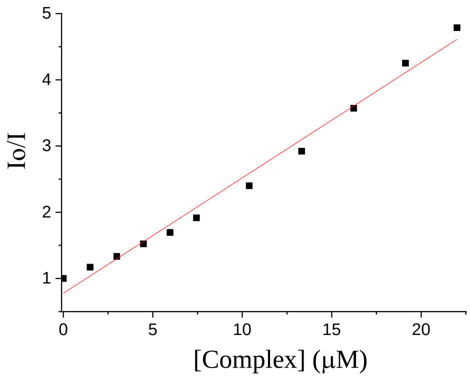

Complex **5**

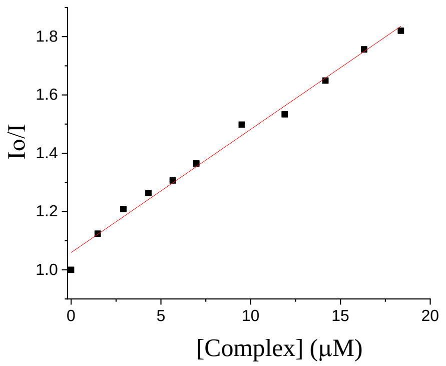

Complex **6**

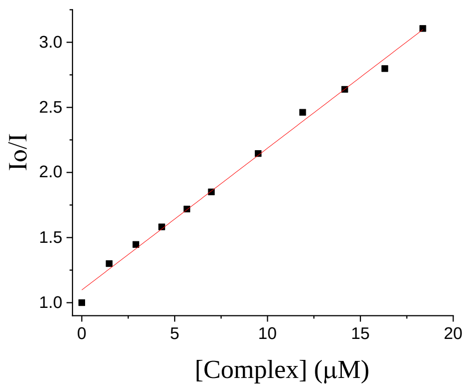

Complex **7**

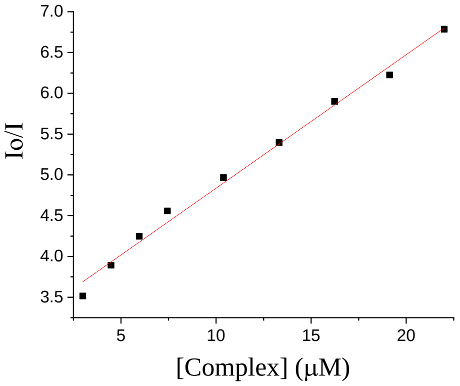

Complex **8**

Figure S16. Scatchard plots of the BSA-quenching experiments upon addition of the compounds.

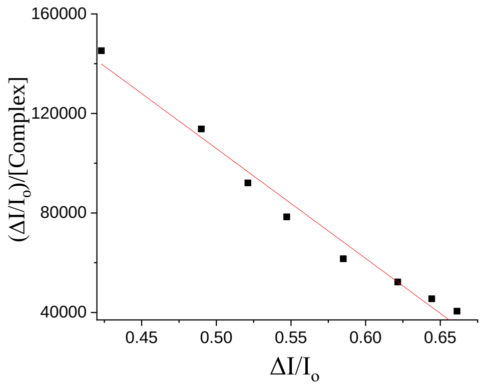	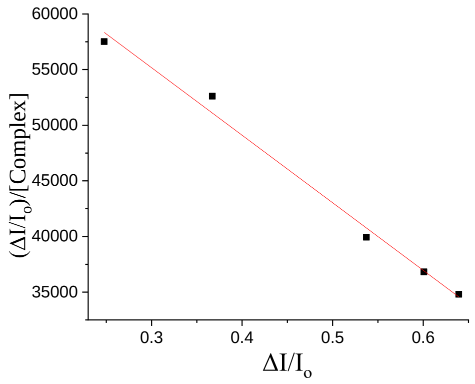
Complex **1**	Complex **2**
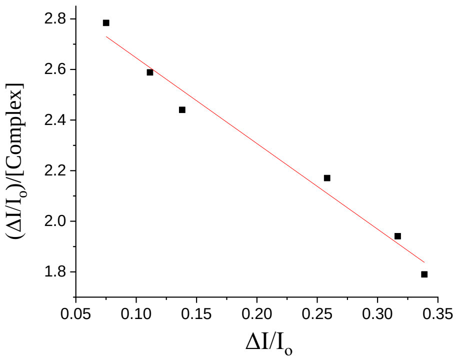	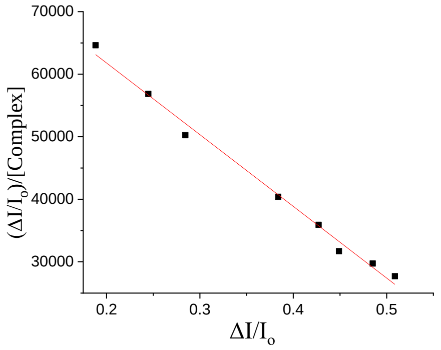
Complex **3**	Complex **4**
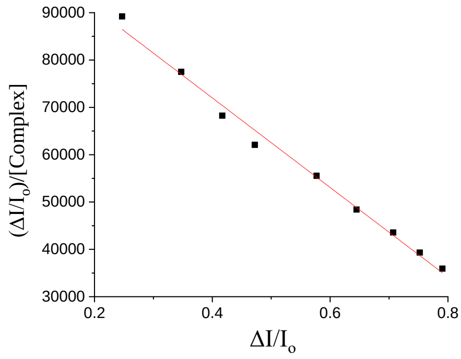	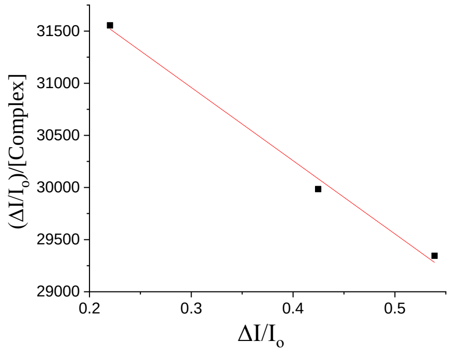
Complex **5**	Complex **6**
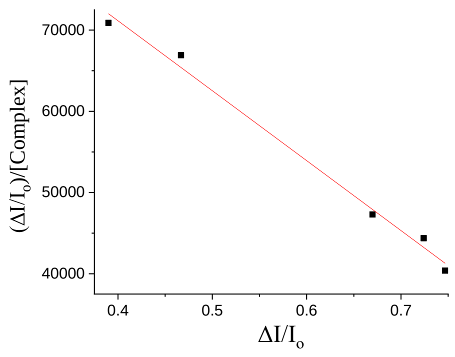	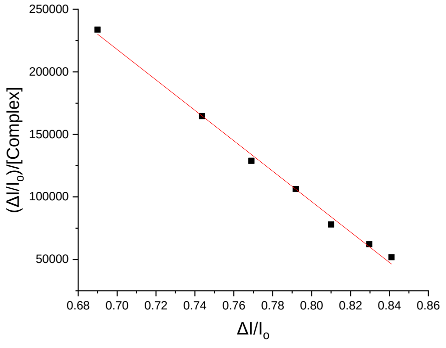
Complex **7**	Complex **8**

Figure S17. Scatchard plots of the HSA-quenching experiments upon addition of the compounds.

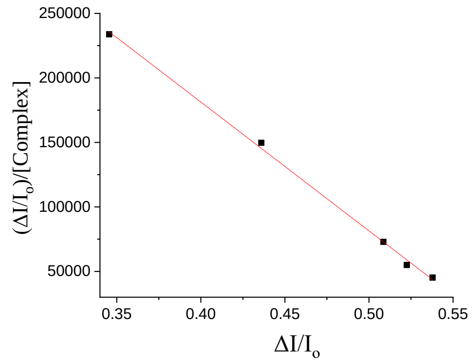

Complex **1**

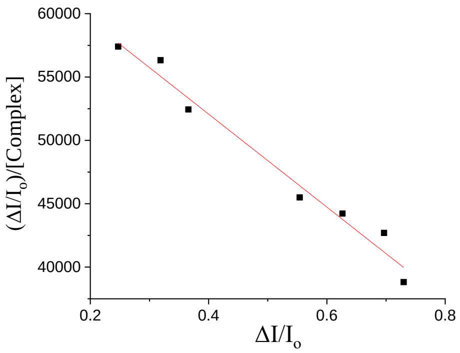

Complex **2**

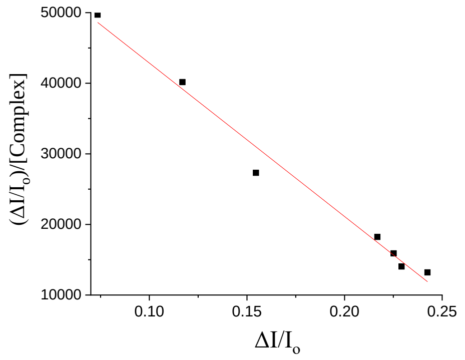

Complex **3**

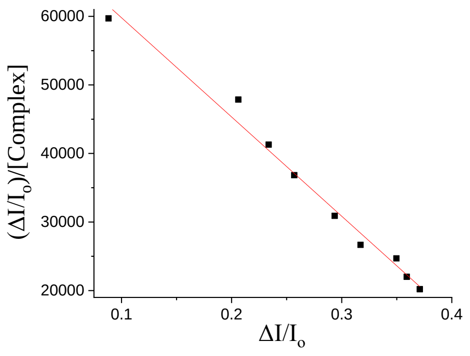

Complex **4**

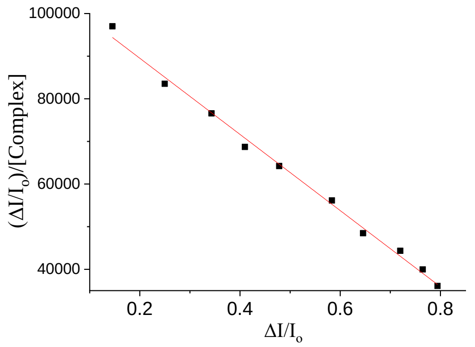

Complex **5**

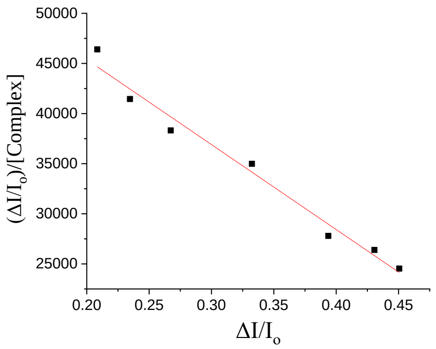

Complex **6**

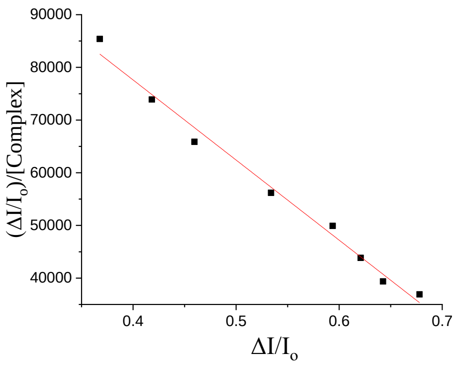

Complex **7**

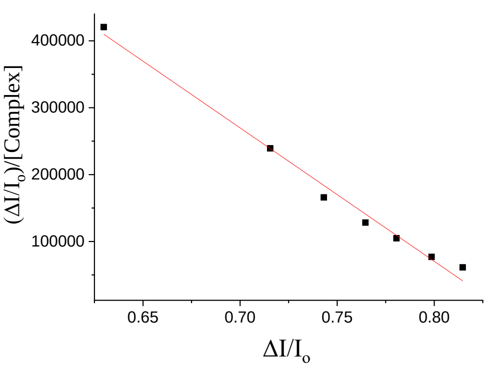

Complex **8**

Figure S18. Fluorescence emission spectra of BSA in buffer solution in the presence of warfarin upon addition of increasing amounts of the compounds.

Conditions: $\lambda_{excitation}$= 295 nm. [BSA] = 3 μM. Buffer solution: 150 mM NaCl and 15 mM trisodium citrate at pH 7.0. [Warfarin] = 3 μM. The arrow shows the changes of intensity upon increasing amounts of the compound.

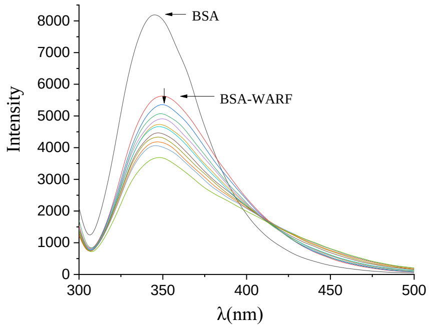

Complex **1**

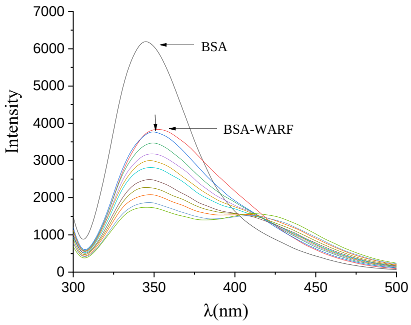

Complex **2**

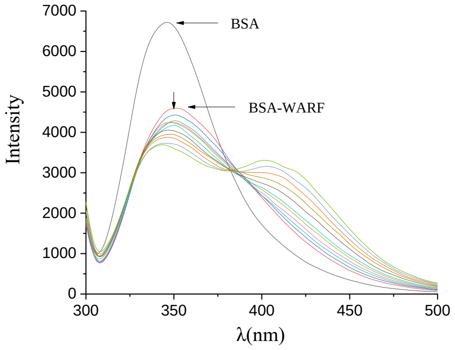

Complex **3**

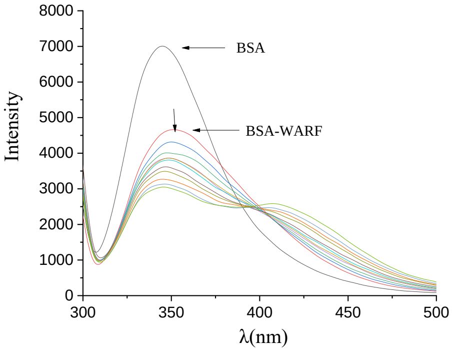

Complex **4**

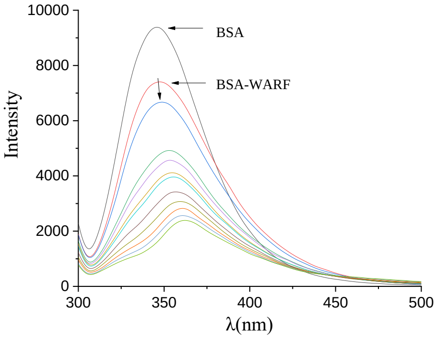

Complex **5**

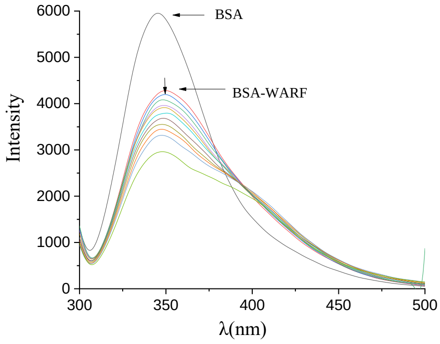

Complex **6**

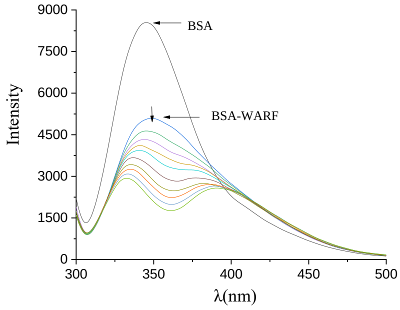

Complex **7**

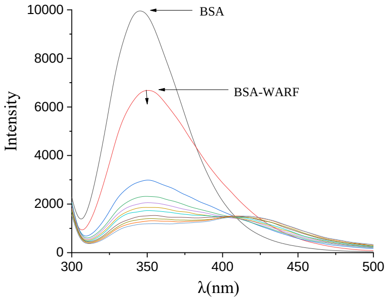

Complex **8**

Figure S19. Fluorescence emission spectra of BSA in buffer solution in the presence of ibuprofen upon addition of increasing amounts of the compounds.
Conditions: $\lambda_{excitation}$ = 295 nm. [BSA] = 3 μM. Buffer solution: 150 mM NaCl and 15 mM trisodium citrate at pH 7.0. [Ibuprofen] = 3 μM. The arrow shows the changes of intensity upon increasing amounts of the compound.

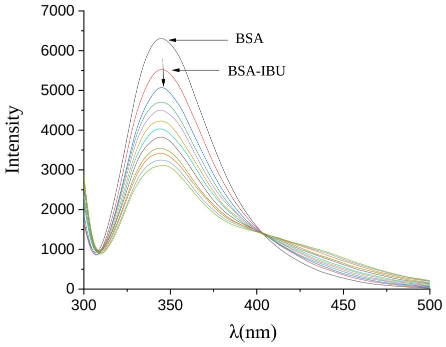

Complex **1**

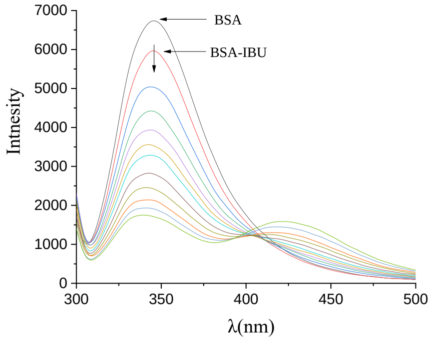

Complex **2**

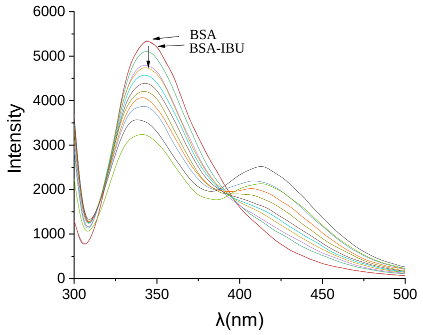

Complex **3**

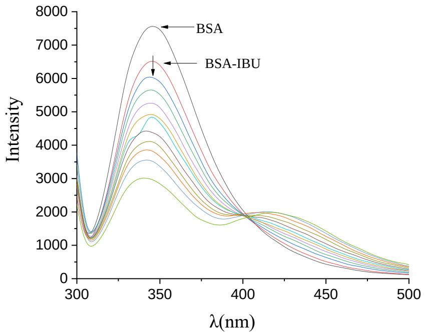

Complex **4**

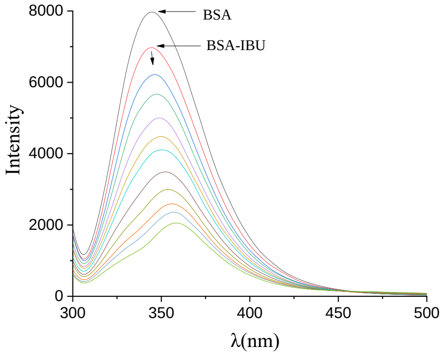

Complex **5**

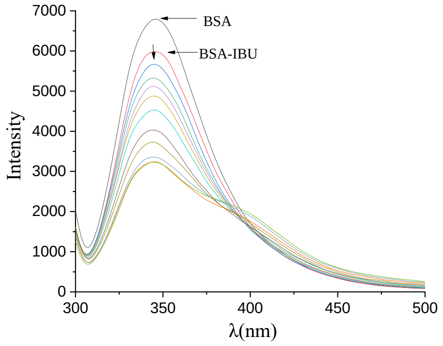

Complex **6**

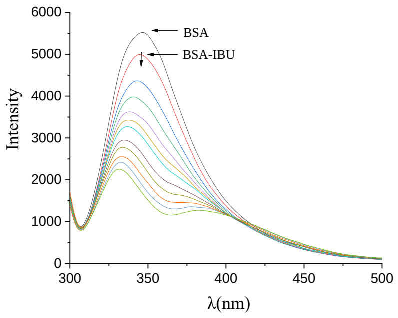

Complex **7**

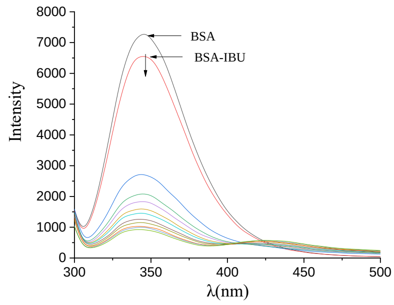

Complex **8**

Figure S20. Fluorescence emission spectra of HSA in buffer solution in the presence of warfarin upon addition of increasing amounts of the compounds.

Conditions: $\lambda_{excitation}$ = 295 nm. [HSA] = 3 μM. Buffer solution: 150 mM NaCl and 15 mM trisodium citrate at pH 7.0. [Warfarin] = 3 μM. The arrow shows the changes of intensity upon increasing amounts of the compound.

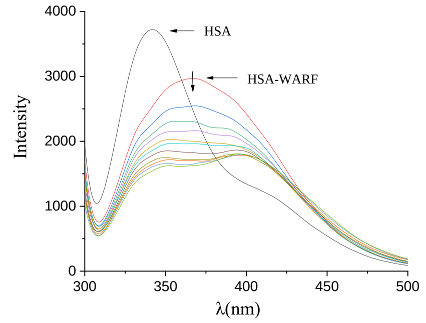

Complex **1**

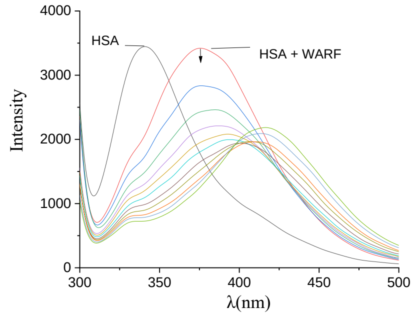

Complex **2**

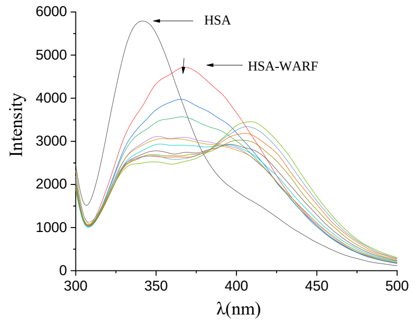

Complex **3**

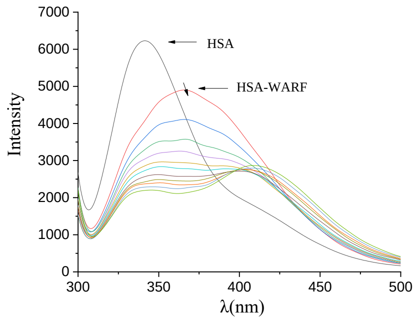

Complex **4**

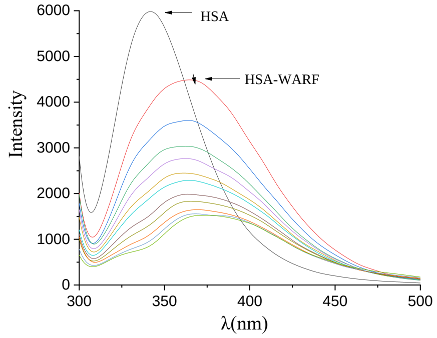

Complex **5**

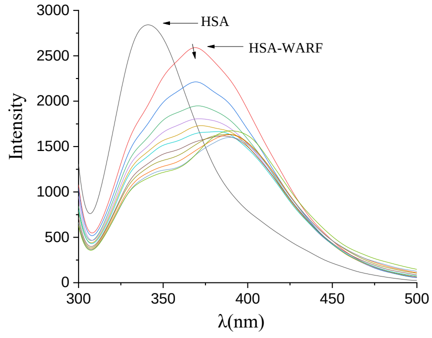

Complex **6**

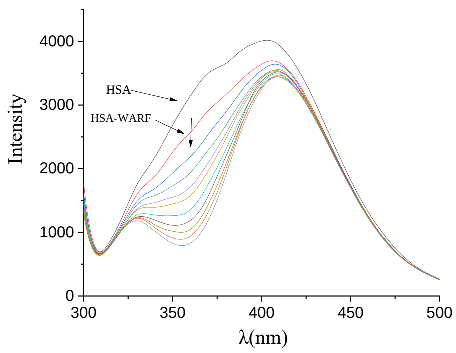

Complex **7**

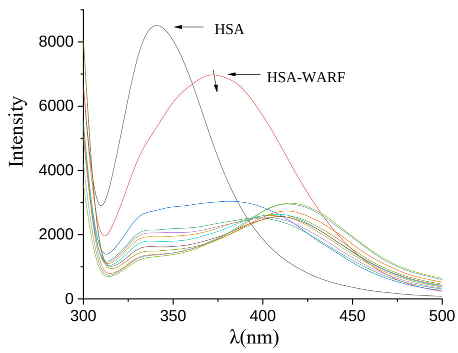

Complex **8**

Figure S21. Fluorescence emission spectra of HSA in buffer solution in the presence of ibuprofen upon addition of increasing amounts of the compounds.
Conditions: $\lambda_{excitation}$ = 295 nm. [HSA] = 3 μM. Buffer solution: 150 mM NaCl and 15 mM trisodium citrate at pH 7.0. [Ibuprofen] = 3 μM. The arrow shows the changes of intensity upon increasing amounts of the compound.

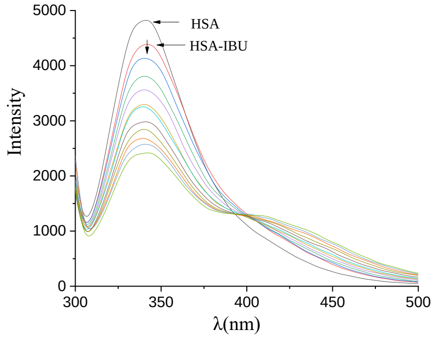

Complex **1**

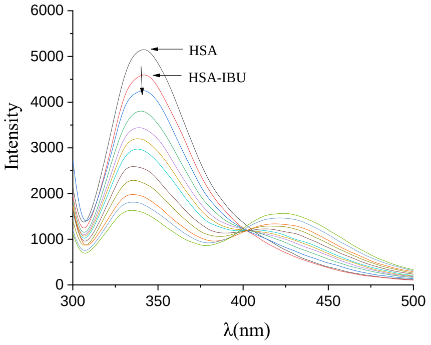

Complex **2**

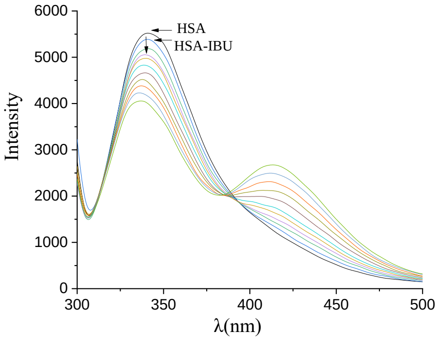

Complex **3**

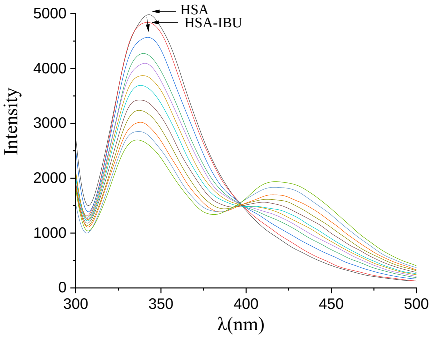

Complex **4**

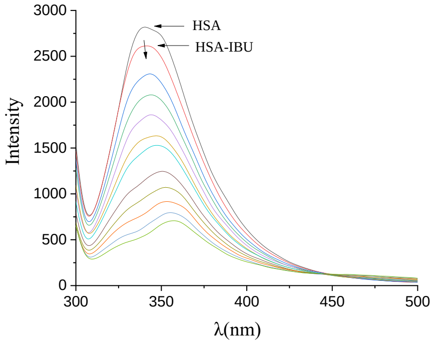

Complex **5**

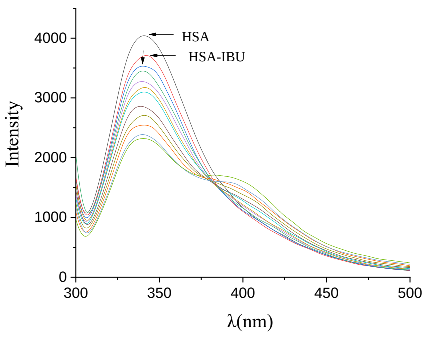

Complex **6**

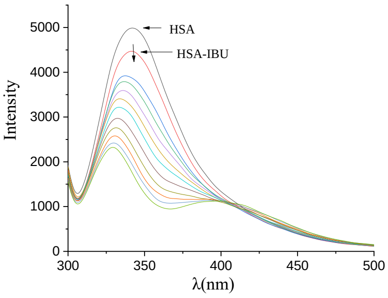

Complex **7**

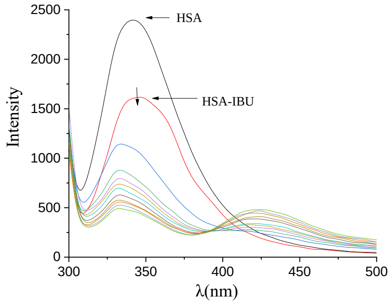

Complex **8**

Figure S22. Scatchard plots of the BSA quenching experiments in the presence of warfarin upon addition of the compounds.

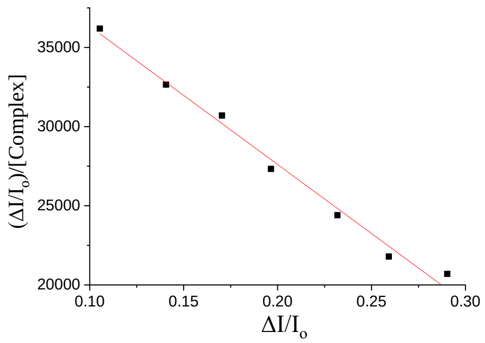	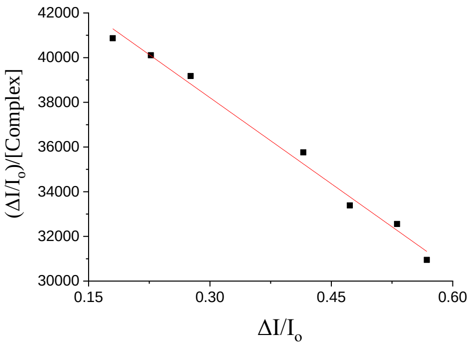
Complex **1**	Complex **2**
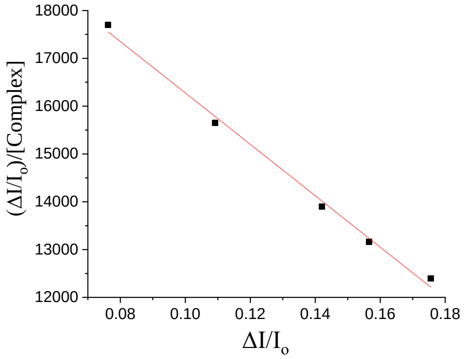	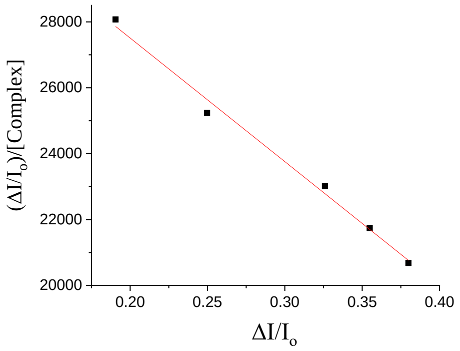
Complex **3**	Complex **4**
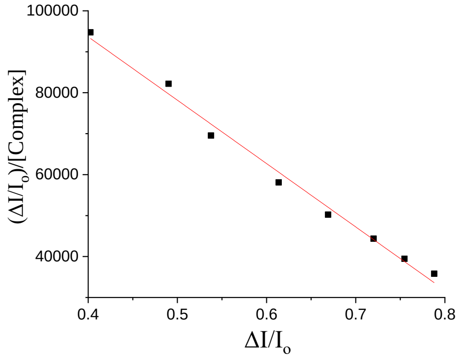	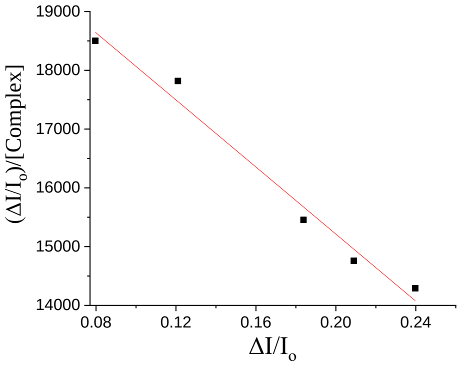
Complex **5**	Complex **6**
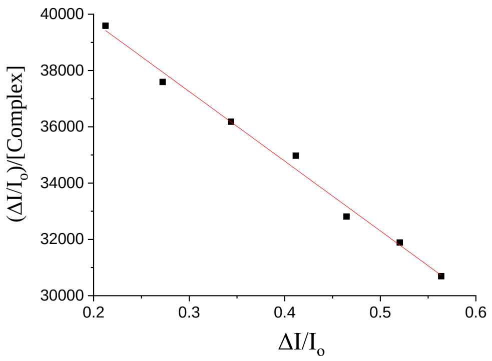	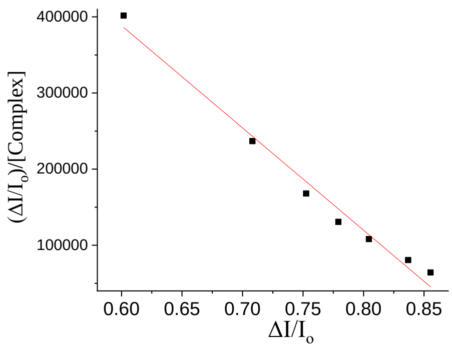
Complex **7**	Complex **8**

Figure S23. Scatchard plots of the BSA quenching experiments in the presence of ibuprofen upon addition of the compounds.

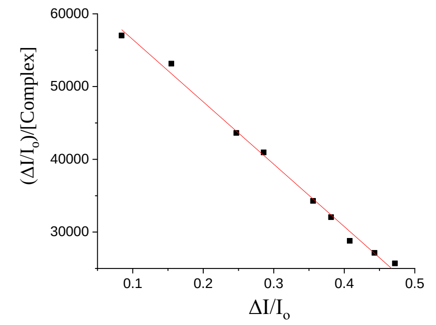	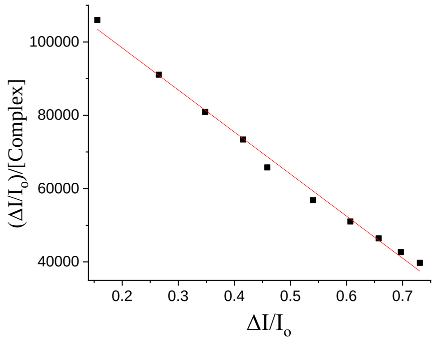
Complex **1**	Complex **2**
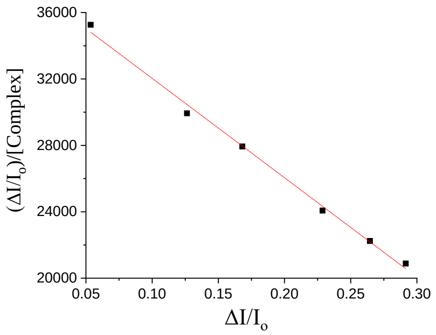	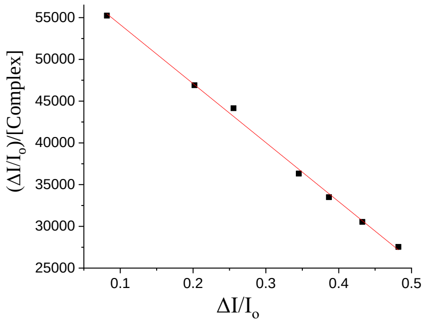
Complex **3**	Complex **4**
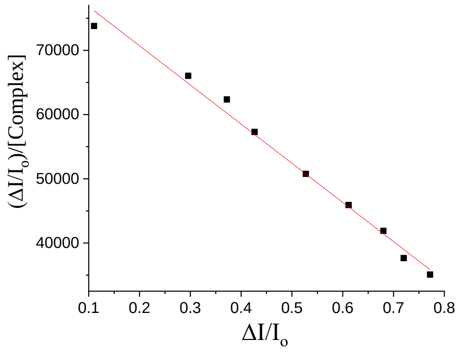	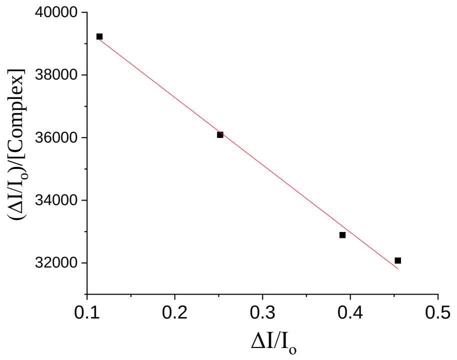
Complex **5**	Complex **6**
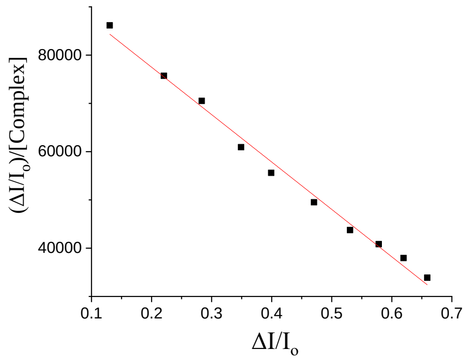	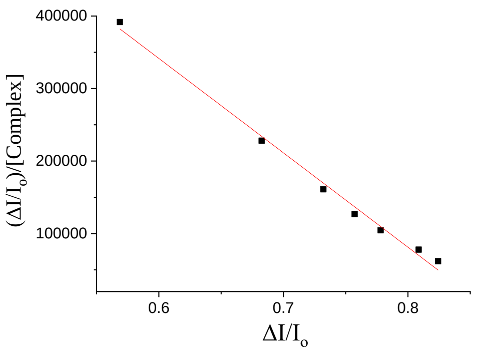
Complex **7**	Complex **8**

Figure S24. Scatchard plots of the HSA quenching experiments in the presence of warfarin upon addition of the compounds.

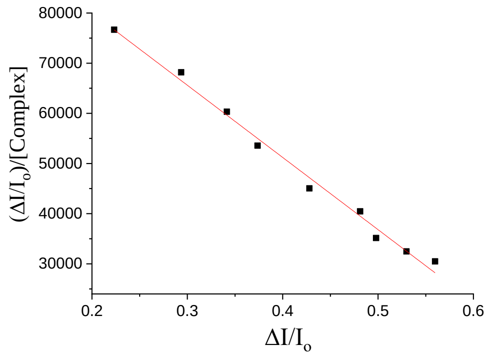

Complex **1**

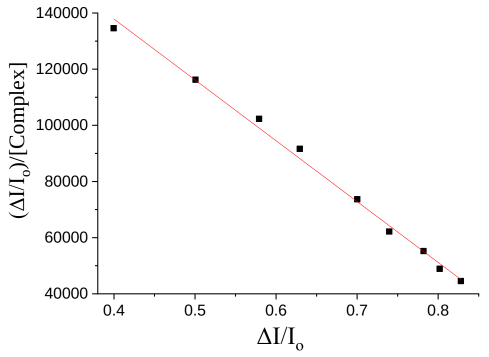

Complex **2**

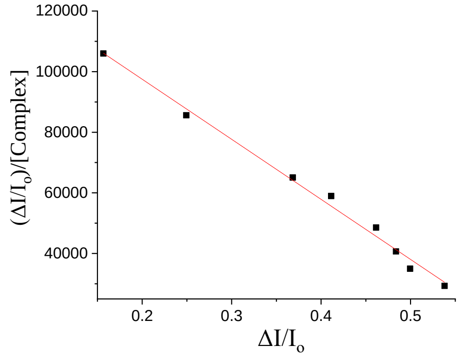

Complex **3**

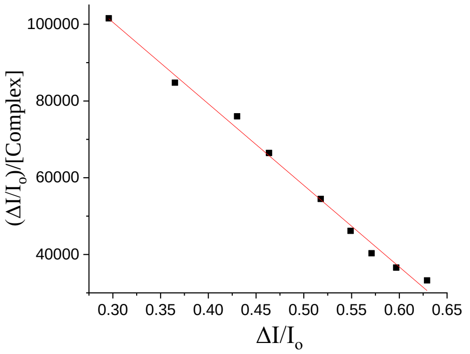

Complex **4**

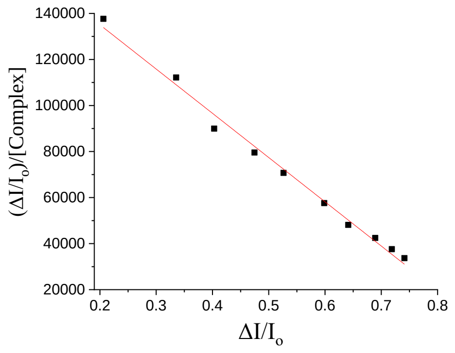

Complex **5**

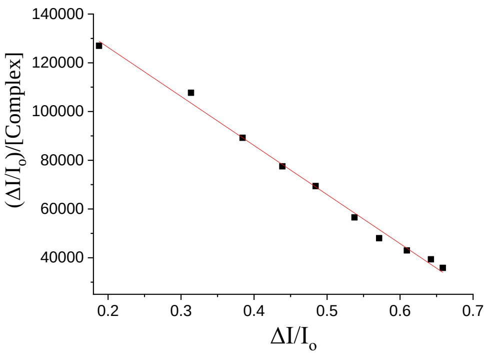

Complex **6**

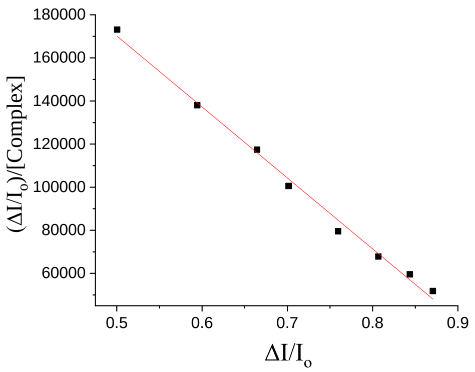

Complex **7**

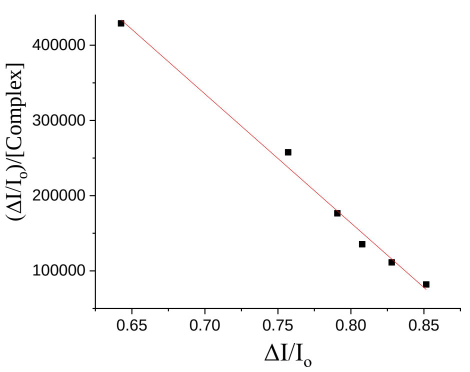

Complex **8**

Figure S25. Scatchard plots of the HSA quenching experiments in the presence of ibuprofen upon addition of the compounds.

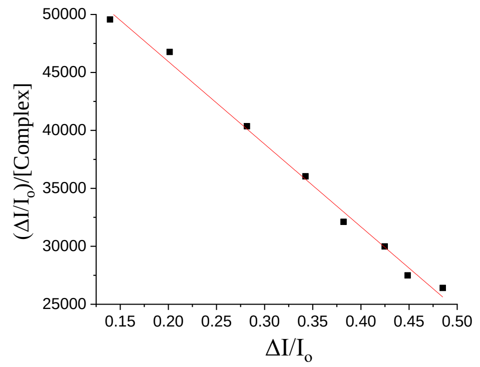

Complex **1**

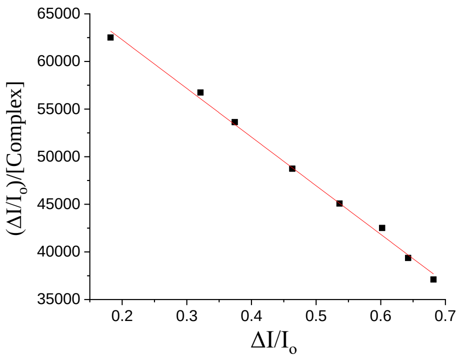

Complex **2**

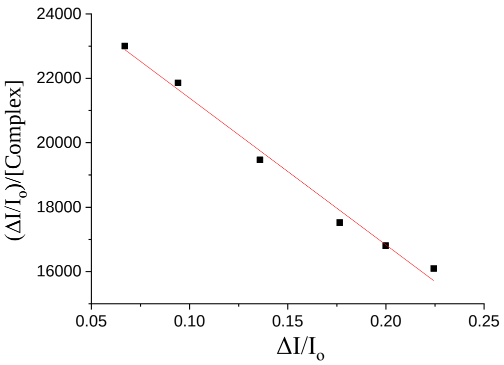

Complex **3**

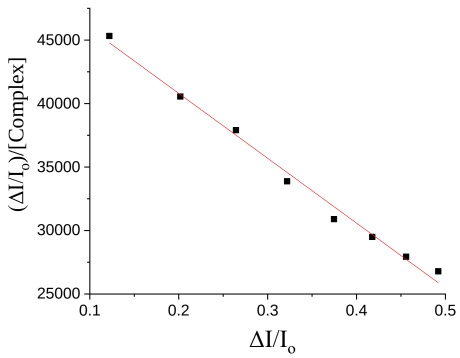

Complex **4**

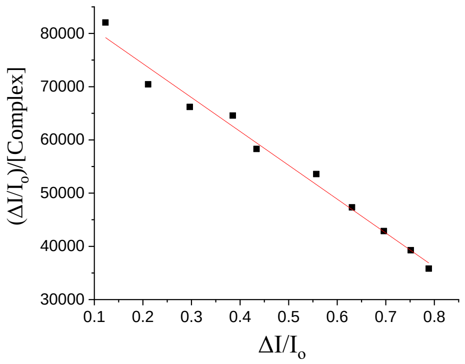

Complex **5**

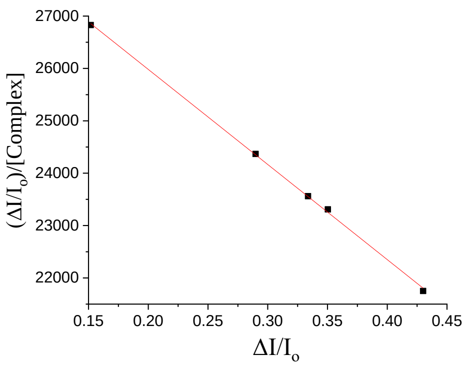

Complex **6**

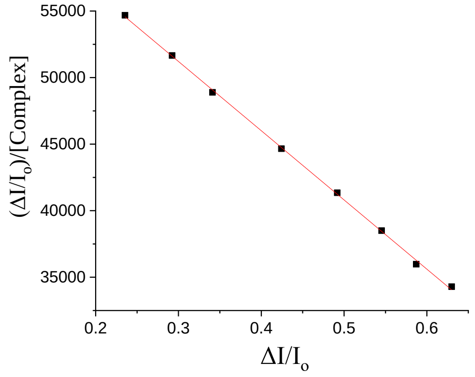

Complex **7**

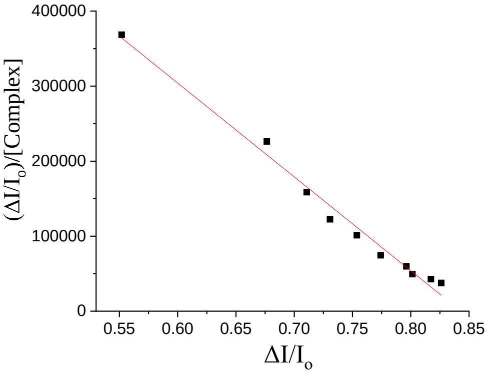

Complex **8**
